# MicroRNA transcriptome analysis of porcine vital organ responses to immunosuppressive porcine cytomegalovirus infection

**DOI:** 10.1186/s12985-018-0922-x

**Published:** 2018-01-18

**Authors:** Xiao Liu, Haoche Wei, Shan Liao, Jianheng Ye, Ling Zhu, Zhiwen Xu

**Affiliations:** 1 Southwest University, College of Animal Science and technology, Chongqing, 400715 China; 2 Key Laboratory of Animal Disease and Human Health of Sichuan Province and Animal Biotechnology Center, College of Veterinary Medicine of Sichuan Agricultural University, 211#Huimin Road, Wenjiang District, Chengdu, Sichuan Province 610000 China; 3 College of Life Sciences, Sichuan University, Chengdu, 610000 China; 4 Department of Urology, Guangdong Key Laboratory of Clinical Molecular Medicine and Diagnostics, Guangzhou First People’s Hospital, Guangzhou Medical University, Guangzhou, 510180 China

**Keywords:** Porcine cytomegalovirus, microRNA transcriptome, Porcine organ, High-throughput sequencing, Integrated expression analysis

## Abstract

**Background:**

Porcine cytomegalovirus (PCMV) is an immunosuppressive virus that mainly inhibits T-lymphocyte and macrophage immune functions; it has significantly damaged the farming industry. Although recent studies have shown that miRNAs play important roles in immune responses, the regulatory mechanisms of miRNAs during immunosuppressive virus infection remain unclear.

**Methods:**

In this study, porcine small-RNA transcriptomes of PCMV-infected and uninfected vital organs were first characterised by high-throughput sequencing. miRDeep2 software was used to predict novel pig-encoded miRNAs. To verify the accuracy of the high-throughput sequencing results, stem-loop qRT-PCR was performed on 12 significantly DE miRNAs.

The physical and functional interactions between the immune-related target genes of the DE miRNAs in PCMV-infected organs were analysed using the STRING database.

**Results:**

In total, 306 annotated and 295 novel miRNAs were identified from PCMV-infected and uninfected porcine organs, respectively, through alignment with known *Sus scrofa* pre-miRNAs. Overall, 92, 107, 95, 77 and 111 miRNAs were significantly differentially expressed in lung, liver, spleen, kidney and thymus after PCMV infection, respectively.

According to Gene Ontology enrichment analysis, target genes of the differentially expressed miRNAs associated with immune system processes, regulation of biological processes and metabolic processes were enriched in every sample. Integrated expression analysis of the differentially expressed miRNAs and their target mRNAs in PCMV-infected thymus showed that the significant differential expression of specific miRNAs under the pressure of PCMV infection in central immune organs interfered with the expression of genes involved in important immune-related signalling pathways, thus promoting the viral infection.

**Conclusions:**

This is the first comprehensive analysis of the responses of host small-RNA transcriptomes to PCMV infection in vital porcine organs. It provides new insights into the regulatory mechanisms of miRNAs during infection by immunosuppressive viruses.

**Electronic supplementary material:**

The online version of this article (10.1186/s12985-018-0922-x) contains supplementary material, which is available to authorized users.

## Background

Porcine cytomegalovirus (PCMV) belongs to the genus *Cytomegalovirus*, subfamily Betaherpesvirinae and family Herpesvirus. It has a 128,367-base-pair (bp) double-stranded DNA genome containing 79 open reading frames. This virus is distributed all over the world, and it mainly spreads through the respiratory tract or placenta [1].

PCMV is an immunosuppressive virus. Clinical observations have shown that PCMV infection is typically accompanied by porcine reproductive and respiratory syndrome virus (PRRSV) infection in immunosuppressed swine, and a series of secondary bacterial infections frequently occur after viral infection. PCMV infection causes reproductive failure in sows, and PCMV-infected piglets less than 3 weeks old often show lethal systemic infections and serious respiratory symptoms, along with high mortality. In addition, the pigs that recover remain latently infected and require long-term detoxification [2, 3].

PCMV has strong antigenic specificity, and no strains with significantly different antigenicity have been identified. In addition, there is no cross-reactivity between PCMV and other herpesviruses [4]. Currently, PCMV is a source of major concern to the breeding industry; however, to the best of our knowledge, there has been little research on the molecular mechanisms behind the immunosuppression induced by PCMV and the prevention of its infection.

These issues have emerged against the background of the development of xenotransplantation technology. This new technology has raised expectations that pig organs could gradually solve the problem of a shortage of organ transplants from human donors, but the high rate of PCMV infection makes this virus a major threat to recipients of xenotransplants [5, 6].

MicroRNAs (miRNAs) are a class of endogenous 18–24-nucleotide (nt) noncoding RNAs. They have post-transcriptional regulatory functions and play important roles in immune responses. miRNAs are ubiquitous among plants and mammals [7, 8], and a large number of studies have shown that they are involved in the regulation of multiple physiological processes, including development, apoptosis and fat metabolism. They have also been shown to be associated with the establishment and development of tumours and viral infections [9, 10].

The first miRNA to be discovered was let-7, which was identified in 2001 in *C. elegans*. It functions in embryo development by regulating the translation of its target genes. Thousands of miRNAs in human, fruit fly, mouse, pig and other organisms have since been identified [11–13]. Since the Epstein–Barr virus (EBV) was first revealed to encode miRNAs in 2004, this phenomenon has also been confirmed in a series of viruses belonging to the Herpesviridae, Polyomaviridae and Retroviridae families. However, no PCMV-encoded miRNAs have been reported [14–16].

A recent report showed that virus-encoded miRNAs manipulate cellular and viral gene expression, and viral infection also has a significant impact on the expression profile of cellular miRNAs. Virus-encoded miRNAs also interfere with host immune-related signalling pathways, thus promoting the viral infection [17–21]. In-depth research on the modulation of host–virus interactions by miRNAs should contribute to a deeper understanding of the molecular mechanisms behind viral latent infection and reactivation, and lead to novel approaches for the treatment and prevention of viral diseases.

Transcriptome analysis of PCMV-infected porcine central immune organs in our previous study showed that PCMV infection suppresses the major immune-related signalling pathways in these organs [22]. However, to the best of our knowledge, the miRNA transcriptomes of PCMV-infected porcine organs and the regulatory roles of miRNAs in immune responses to PCMV infection in different vital porcine organs have not been investigated.

To further investigate the immunosuppressive mechanisms associated with PCMV infection, we obtained global small-RNA expression profiles of PCMV-infected and uninfected porcine organs. We then identified the annotated and novel miRNAs from the lungs, liver, kidneys, spleen and thymus of PCMV-infected and uninfected pigs.

The putative target genes of the differentially expressed (DE) miRNAs were predicted. We also screened host-encoded miRNAs that may play regulatory roles during viral infection. The results of the integrated expression analysis of DE miRNAs and their target mRNAs in PCMV-infected central immune organs should contribute to our understanding of the mechanisms of viral immunosuppression.

## Methods

### Ethics

The animal welfare standards in this study were established on the basis of internationally agreed and science-based principles within the World Organisation for Animal Health. All experiments were carried out in accordance with Chinese animal welfare legislation and were approved by Sichuan Agricultural University Committee on Ethics in the Care and Use of Laboratory Animals.

### Data sources

The NCBI GEO accession number for the high-throughput sequencing data reported in this paper is GSE76156. Transcriptome data of PCMV-infected thymus were obtained from the NCBI (NCBI GEO accession: GSE59115, ID: 200,059,115).

The *S. scrofa* genomic sequence and gene annotation data were obtained from the University of California Santa Cruz (UCSC) Genome browser (http://genome.ucsc.edu/index.html). The PCMV complete genomic sequence was obtained from the NCBI GenBank Genome database (http://www.ncbi.nlm.nih.gov/nuccore/KF017583.1) (accession no.: KF017583.1). The annotated pig-encoded miRNAs are available in miRBase (http://www.mirbase.org/).

### Sample collection and RNA extraction

Ten 3-week-old PCMV antibody- and antigen-negative Yorkshire piglets (provided by Sichuan Agricultural University) were divided into infected and uninfected control groups. Each piglet in the PCMV-infected group was inoculated with 5 ml of 10^9^ plaque-forming units/ml PCMV SC strain, and each piglet in the uninfected group was injected with 5 ml of RPMI-1640 medium (Thermo Fisher Scientific, Waltham, UK). Liver, kidney, thymus, spleen and lung samples were obtained from PCMV-infected (*n* = 5) and uninfected (*n* = 5) piglets at 14 days postinfection.

Total RNA was extracted from PCMV-infected and uninfected samples using Trizol reagent (Invitrogen, Carlsbad, CA, USA), in accordance with the manufacturer’s instructions. The NanoDrop ND-1000 instrument (Nano Drop Inc., Wilmington, DE, USA) was used for accurate measurement of the concentration (OD_260_) of the total RNA samples.

This study study used pooled samples and experiments were performed in triplicate. Namely, all liver, kidney, thymus, spleen and lung samples from PCMV-infected and uninfected pigs were combined into single samples representing each of the PCMV-infected and uninfected lung, liver, spleen, kidney and thymus groups. The presence of PCMV infection in every infected and uninfected porcine organ was determined by PCR.

### miRNA expression profiling

The total RNA of PCMV-infected and uninfected lungs, liver, spleen, kidneys and thymus was isolated using Trizol reagent (Invitrogen, Waltham, MA, USA). After 3′- and 5′-adapter ligation with T4 RNA ligase, cDNA was synthesised using RT primer, and PCR products with a length of 120–140 bp were purified using polyacrylamide gel. The high-quality samples from PCMV-infected and uninfected groups were diluted to a concentration of 8 pM for cluster generation using TruSeq Rapid SR cluster kit (Illumina, San Diego, CA, USA), following the manufacturer’s instructions. miRNA high-throughput sequencing was performed using Illumina HiSeq 2000 (Illumina, San Diego, CA, USA) for 36 cycles.

### Sequencing data analysis

Basecaller software (OLB v1.8.0) was used to analyse sequencing images. Subsequently, the raw data were filtered using a Solexa CHASTITY quality filter, reads with an average quality of less than 15 nt were discarded and miRNAs were distinguished from other small RNAs. All 3′-adapter-trimmed reads (≥15 nt) were used as queries for a Blast search against known porcine reference miRNA precursor sequences (miRBase 19) using Novoalign (v2.07.11) software. The variability of mature miRNA isomiRs was then analysed. The significantly differentially expressed miRNAs in the infected samples (vs. the uninfected ones) were determined by their fold change; miRNA with a fold change value (log_2_) ≥ 2.0 was considered to be significantly upregulated, while that with a fold change (log_2_) ≤ 0.5 was considered to be significantly downregulated in PCMV-infected samples. (DE miRNAs were identified based on the normalised most abundant tag counts).

### Analysis of novel miRNAs

miRDeep2 software was used to predict novel miRNAs in 10 high-throughput sequencing profiles. The secondary structures of the novel miRNAs were predicted using Mfold software and the Vienna RNAfold web server.

### Target prediction and functional enrichment of miRNAs

miRNA target genes of the DE miRNAs in PCMV-infected samples were predicted using the database miRGen 3.0 (http://www.diana.pcbi.upenn.edu/miRGen.html) (combining PicTar, miRnada and TargetScanS online tools). The positional relationships between miRNAs and genomic annotation sets were also analysed.

The filtered target genes of the DE miRNAs were subjected to Gene Ontology (GO) analysis using the WEGO software (http://wego.genomics.org.cn/cgi-bin/wego/index.pl). GO terms with a *p*-value ≤ 0.05 were assumed to be significant.

The STRING online prediction tool was used to predict the interactions between target genes of the DE miRNAs in each sample. The interactions of DE miRNAs and their predicted target mRNAs in PCMV-infected thymus were also analysed.

### qRT-PCR validation of DE miRNAs

Stem-loop RT-PCR was used to confirm the expression level of 12 selected DE miRNAs in PCMV-infected and control organs. Stem-loop RT-PCR was performed in triplicate. Reactions were performed for each miRNA using the transcription product and the SYBR Green PCR Core Reagents Kit (Applied Biosystems, Foster City, CA, USA) on an ABI Prism 7900 Sequence Detection System (Applied Biosystems), in accordance with the manufacturer’s instructions. The miRNA-specific forward primers and universal reverse primers are shown in Additional file 1: Table S7. In this study, a relative quantitation PCR method was used to evaluate the expression level of selected DE miRNAs with the U6 gene as an internal control. Specifically, the 2^−ΔΔCt^ method was used to analyse the data.

## Results

### Overview of the high-throughput sequencing data

**Fig. 1:**
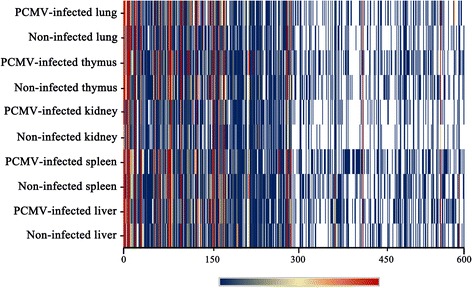
Heat map and hierarchical clustering of the miRNA high-throughput sequencing data. Heat map of the expression level of miRNAs in PCMV-infected and uninfected lung, thymus, kidney, spleen and liver samples. A red line indicates a higher expression level and a blue line indicates a lower expression level

The results of polymerase chain reaction (PCR) confirmed that all of the investigated organs had been successfully infected with PCMV. To investigate the miRNA transcriptome of PCMV-infected and uninfected porcine organs, we generated 10 miRNA expression profiles of liver, kidney, thymus, spleen and lung samples from PCMV-infected and uninfected Yorkshire piglets based on the Illumina HiSeq 2000 platform (Fig. 1).

The generated clean reads, adapter-trimmed reads [length ≥ 15 nt] and the reads aligned to known *Sus scrofa* pre-miRNA in the database of uninfected and PCMV-infected samples are shown in Additional file 1: Table S1. Most of the 38,988,425 adapter-trimmed reads of the uninfected and PCMV-infected groups had a length of 22 nt (Additional file 1: Figure S1). This indicates the successful enrichment of mature miRNAs in the PCMV-infected and uninfected sequencing libraries. Next, we aligned the reads to the latest known pig genome (Ensembl) using Novoalign software (v2.07.11); most of the reads mapped to the pig genome.

Among the annotated small RNAs [ribosomal RNA (rRNA), transfer RNA (tRNA), small RNA (sRNA), miRNA, small nuclear RNA (snRNA) and other non-coding RNA (ncRNA)] in the expression profiles, > 84% were categorised as miRNAs (Additional file 1: Figure S2). The expression levels of the top 10 miRNAs in each expression profile are shown in Additional file 1: Figure S3.

### Identification of annotated and novel miRNAs

miRDeep2 software was used to predict novel pig-encoded miRNAs. Data analyses suggested that 430 (269 annotated and 161 novel) miRNAs were transcribed in PCMV-infected and control lung samples, 382 (253 annotated and 129 novel) miRNAs were transcribed in PCMV-infected and control liver samples, 433 (272 annotated and 161 novel) miRNAs were transcribed in PCMV-infected and control spleen samples, 324 (242 annotated and 82 novel) miRNAs were transcribed in PCMV-infected and control kidney samples, and 477 (276 annotated and 201 novel) miRNAs were transcribed in PCMV-infected and control thymus samples. In an analysis combining data from all 10 miRNA expression libraries, 306 annotated miRNAs were obtained and the precursors of 295 unannotated miRNAs were identified (Additional file 1: Table S2). In addition, 232 miRNAs were found to be expressed in both PCMV-infected and healthy control samples.

### miRNA expression profiles of different tissues after PCMV infection

**Fig. 2:**
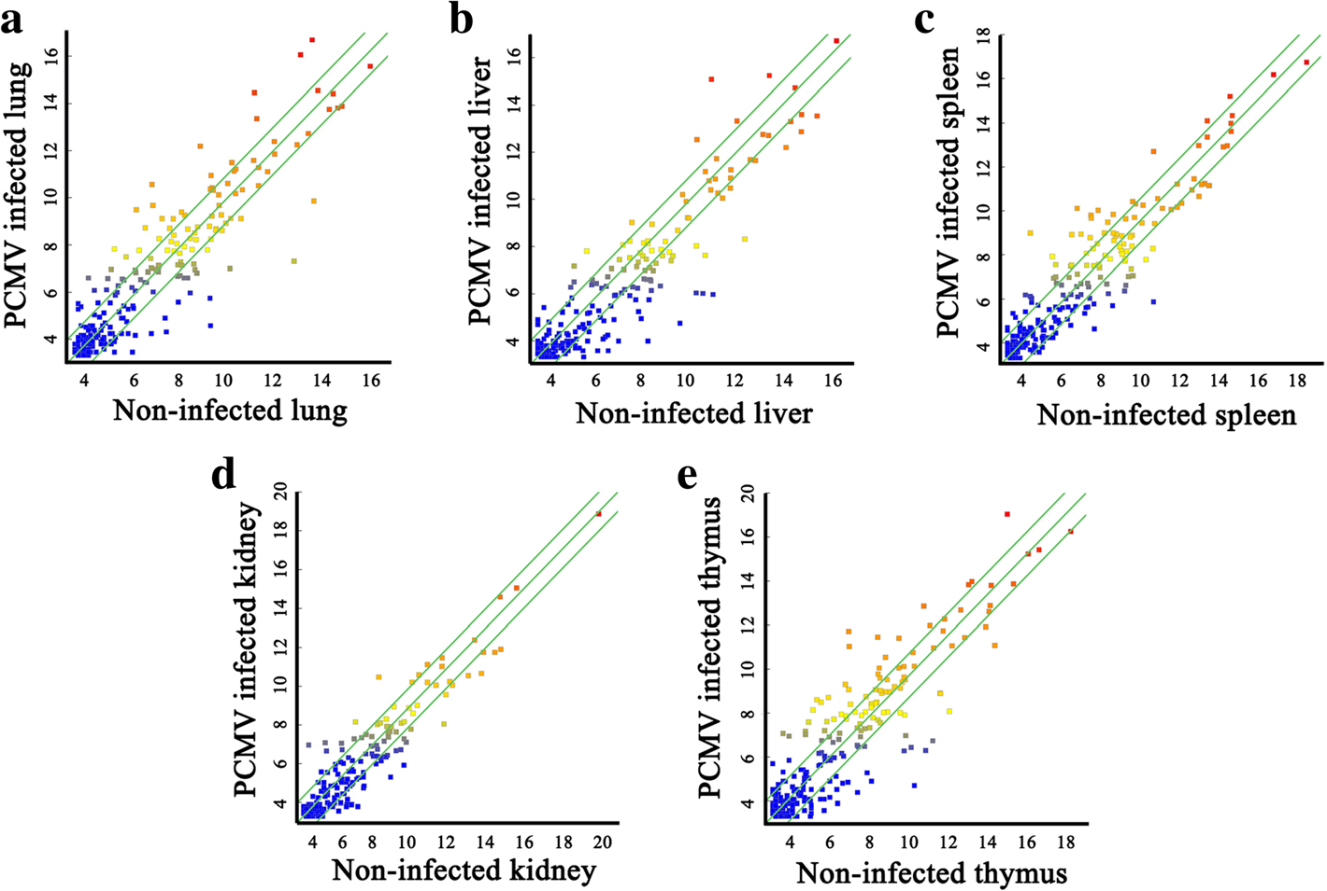
Scatter plot of the high-throughput sequencing data. **a**–**e** The scatter plot shows the variations in miRNA expression profiles between PCMV-infected and uninfected lung, liver, spleen, kidney and thymus. The default fold change value is 2.0 (top and bottom green lines)

The data showed that a small number of miRNA genes were the source of most of the mature miRNAs. For example, 3.6% (14/394) of the total number of different miRNAs accounted for 76.2% of the total miRNA expression in uninfected thymus, while the corresponding values were 3.6% (14/391) and 81.4% in PCMV-infected thymus (Additional file 1: Table S2). Notably, although the miRNA expression profiles of uninfected control and infected groups changed significantly after PCMV infection, some of the miRNA expression levels remained consistent in the expression profile of all samples. For example, ssc-let-7a, ssc-let-7c, ssc-let-7f, ssc-let-7 g and ssc-let-7i from the let-7 family and ssc-miR-21 maintained high transcription levels in all samples, which is consistent with previous findings [23] (Additional file 1: Figure S3). The data on the DE miRNAs in PCMV-infected samples are shown in Fig. 2.

**Fig. 3:**
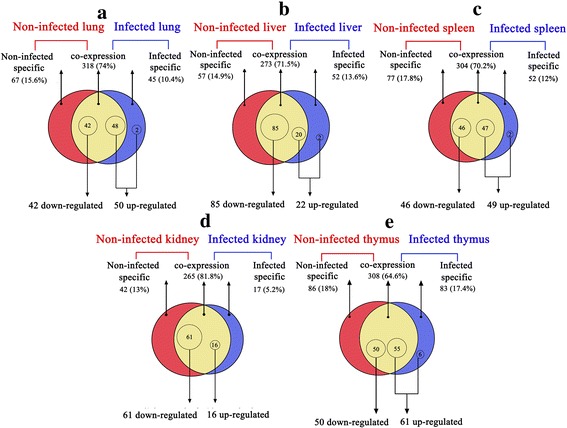
DE miRNAs between uninfected and PCMV-infected tissue samples. **a**–**e** Venn diagrams of the distribution of miRNAs between uninfected and PCMV-infected lung, liver, spleen, kidney and thymus samples. In total, 92, 107, 95, 77 and 111 miRNAs were identified as being differentially expressed in PCMV-infected lung, liver, spleen, kidney and thymus samples, compared with their levels in the uninfected controls (*p* < 0.0001)

In total, 92, 107, 95, 77 and 111 miRNAs were found to be significantly differentially expressed in PCMV-infected lung, liver, spleen, kidney and thymus samples, respectively, compared with their levels in uninfected samples. Of these, 50, 22, 49, 16 and 61 miRNAs were upregulated (≥ 100% increase) and 42, 85, 46, 61 and 50 miRNAs were downregulated (decrease to ≤ 50%) in PCMV-infected lung, liver, spleen, kidney and thymus samples, respectively, compared with their levels in the uninfected samples (Fig. 3) (Additional file 1: Table S3).

ssc-miR-24-3p (172% increase), ssc-miR-199a-5p (1000% increase), ssc-miR-214 (3012% increase), ssc-miR-425-5p (1750% increase) and ssc-miR-199a-5p (2270% increase) were the most upregulated in PCMV-infected lung, liver, spleen, kidney and thymus samples, respectively, compared with their levels in the uninfected samples.

ssc-miR-106a (decrease of 97.1%), ssc-miR-214 (decrease of 97.8%), ssc-miR-425-5p (decrease of 96.2%), ssc-miR-126-3p (decrease of 84.7%) and ssc-miR-24-3p (decrease of 98.4%) were the most downregulated miRNAs in PCMV-infected lung, liver, spleen, kidney and thymus samples, respectively, compared with their levels in the uninfected samples (Additional file 1: Table S3).

### Stem-loop reverse-transcription (RT)-PCR validation of DE miRNAs

To verify the accuracy of the high-throughput sequencing results, stem-loop quantitative (q)RT-PCR was performed on 12 significantly DE miRNAs (ssc-miR-10b, ssc-miR-486, ssc-miR-24-3p, ssc-miR-195, ssc-miR-19b, ssc-let-7f, ssc-miR-146b, ssc-miR-novel-chr16_17559, ssc-miR-novel-GL892871-2_41708, ssc-miR-novel-chr2_21624, ssc-miR-novel-chr12_7961, and ssc-miR-26a). These were selected based on their biological significance and expression level. The stem-loop RT-PCR results were generally consistent with the high-throughput sequencing data (Table 1).

**Table 1:** Comparison of qRT-PCR and high-throughput sequencing results

MicroRNAs	Organs	qRT-PCR fold-change (Infected/control)	High-throughput sequencing fold-change (Infected/control)
miR-10b	Lung	+2.78	+8.04
miR-novel-chr16_17559	Lung	+2.13	+2.50
miR-novel-GL892871-2_41708	Lung	+2.72	+4.27
miR-26a	Lung	+2.73	+7.36
miR-486	Lung	+2.40	+2.41
miR-24-3p	Lung	−0.55	−0.02
miR-195	Lung	−0.29	−0.02
miR-486	Thymus	+3.30	+2.74
miR-novel-chr16_17559	Thymus	+12.76	+9.35
miR-novel-chr2_21624	Thymus	+4.59	+6.68
miR-19b	Thymus	−0.48	−0.18
miR-486	Kidney	+2.15	+2.59
miR-novel-chr2_21624	Kidney	−0.13	−0.27
let-7f	Spleen	+3.97	+2.77
miR-24-3p	Spleen	+14.66	+17.77
miR-146b	Spleen	−0.38	−0.38
miR-19b	liver	−0.13	−0.044
miR-novel-chr12_7961	liver	−0.18	−0.48
miR-novel-chr2_21624	liver	−0.47	−0.43

“+” and “–” indicate upregulated and downregulated miRNAs, respectively. The fold change cutoffs of the upregulated miRNAs and the downregulated miRNAs were +2 and −0.5, respectively. The qRT-PCR Ct threshold is 0.015

### Functional annotation of the target genes of DE miRNAs

**Fig. 4:**
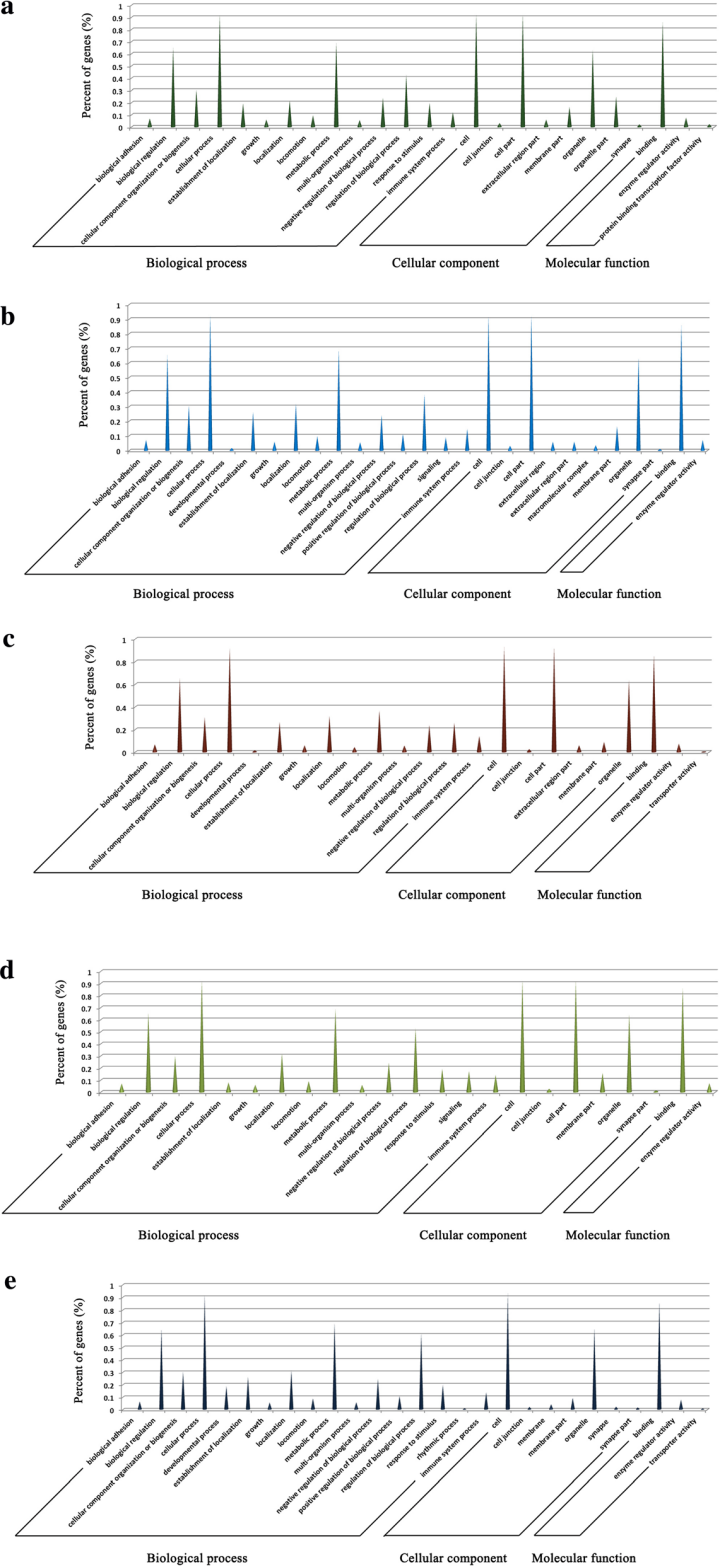
GO annotation of target genes of DE miRNAs. GO functional analysis indicating the regulatory role of DE miRNAs in different organs during PCMV infection. **a**–**e** Target genes of DE miRNAs in PCMV-infected lung, liver, spleen, kidney and thymus

To obtain a deeper understanding of the function of miRNAs in PCMV-infected tissues, the target genes of DE miRNAs in PCMV-infected and uninfected organs were predicted using databases of miRNA targets for which the findings had been confirmed experimentally. The target genes and target sites of the miRNAs are shown in Additional file 1: Table S4. GO analysis was used to provide functional annotations of the DE miRNA targets. The GO results showed that the target genes of DE miRNAs specific to PCMV-infected porcine organs were significantly associated with immune system processes, regulation of biological processes, cellular processes, metabolic processes and nucleic acid binding (Fig. 4, Additional file 1: Figure S4). This demonstrated that these DE miRNAs mainly regulate the immune response of the host during viral infection.

### STRING analysis

**Fig. 5:**
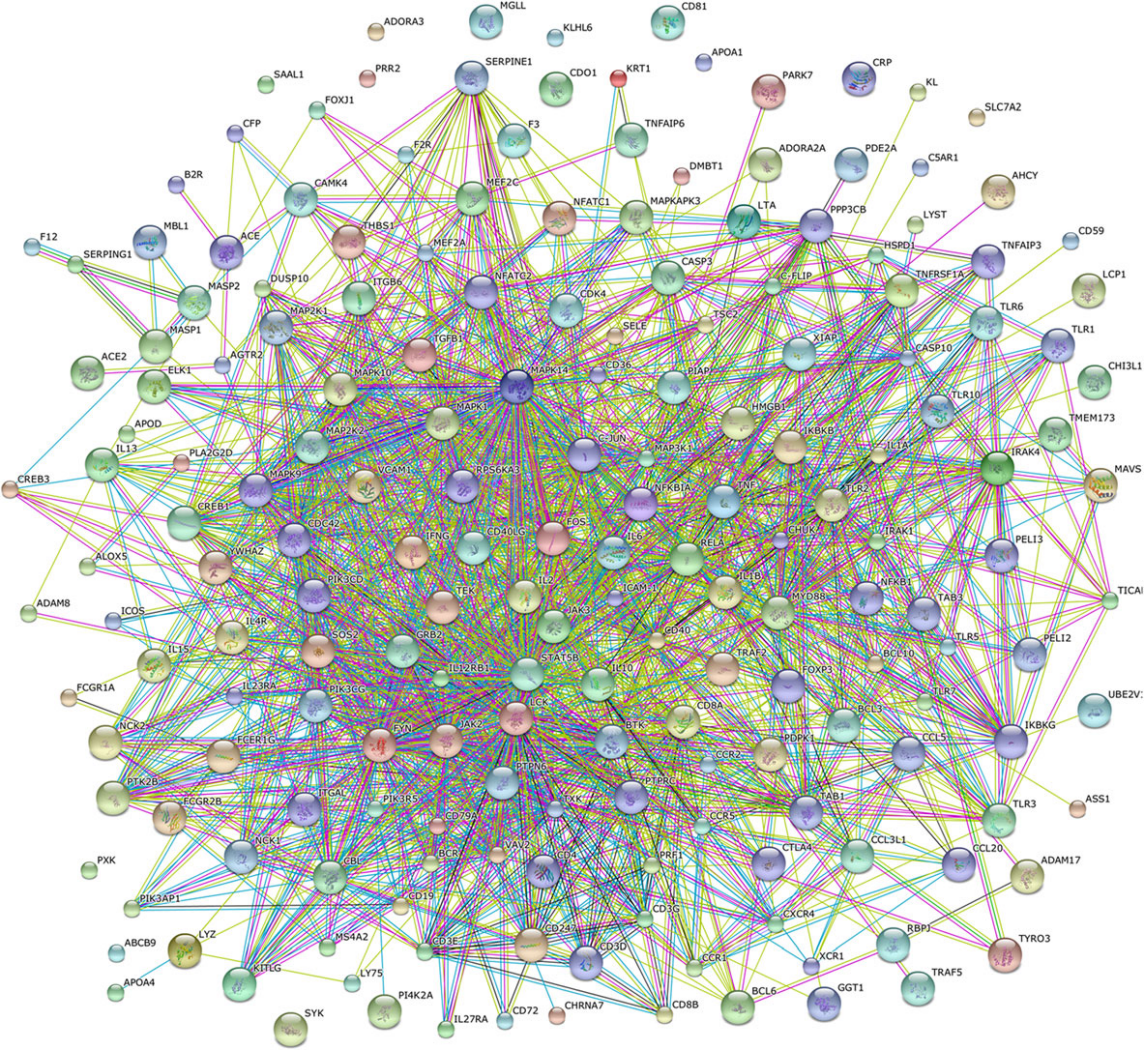
STRING analysis of immune-related target genes of DE miRNAs. Green, red, blue, black, purple, cyan and yellow lines represent neighbourhood evidence, gene fusion evidence, co-occurrence evidence, coexpression evidence, database evidence, text-mining evidence and homology evidence, respectively

The physical and functional interactions between the immune-related target genes of the DE miRNAs in PCMV-infected organs were analysed using the STRING (Search Tool for the Retrieval of Interacting Genes/Proteins) database. According to the functional protein association networks, most of the immune-related target genes (mainly related to immune responses, cytokine signalling pathways and inflammatory responses) of DE miRNAs are interrelated (Fig. 5). For example, the target genes encoding toll-like receptor 1 (TLR1), TLR3, chemokine (C-X-C motif) receptor 4 (CXCR4), chemokine (C-C motif) receptor 1(CCR1), T-cell surface glycoprotein CD4, interleukin-1 receptor-associated kinase 4 (IRAK4), interleukin-1α (IL1α), IL6, tumor necrosis factor (TNF), Janus Kinase 2 (JAK2), Janus Kinase 3 (JAK3), Tyrosine-protein kinase Fyn (FYN) and signal transducer and activator of transcription 5B (STAT5B) were interrelated. However, CD81, apolipoprotein A-I (APOA1), Kelch-like protein6 (KLHL6) and pinoresinol reductase 2 (PRR2) were not linked to the association network.

### Integrated expression analysis of DE miRNAs and their target mRNAs during PCMV infection

Because miRNAs regulate the expression of their target genes by inhibiting mRNA translation and degrading the target transcript, significant changes in the expression level of specific miRNAs will alter the expression of mRNA. Since the thymus is the central immune organ in mammals and is the dominant site for the differentiation and function of T cells, significant differential expression of miRNAs and changes in the transcriptome of the thymus will affect the host antiviral response [24]. As such, in this study, integrated expression analysis of DE miRNAs and their target mRNAs in PCMV-infected thymus was performed. The results showed that the expression levels of DE miRNAs (top 6 upregulated and top 6 downregulated) were negatively correlated with their target DE mRNAs (Additional file 1: Table S6) in response to PCMV infection in the thymus. In total, 548 downregulated and 461 upregulated mRNAs were identified to be the target mRNAs of the top 6 upregulated and top 6 downregulated miRNAs in PCMV-infected thymus. For example, miR-143-3p (568% increase) was significantly upregulated in PCMV-infected thymus, but its target mRNAs DDX58 (decrease of 80.4%), CD40 (decrease of 82.5%) and IL2RG (decrease of 83.4%) were downregulated in the same sample. Pairs of interacting DE miRNAs and predicted target mRNAs belonging to the Toll-like receptor (TLR) and RIG-I-like receptor (RLR) signalling pathways in PCMV-infected thymus are shown in Table 2.

**Table 2:** Pairs of interacting DE miRNAs and predicted target mRNAs (TLR and RLR signalling pathways) in PCMV-infected thymus

miRNAs	miRNA expression level PCMV infected vs. control Log2 (Fold change)	miRNA target Genes	Target mRNA expression level PCMV infected vs. control Log2 (Fold change value)	Target gene related signaling pathways
miR-10a-5p	+2.53	TLR6	−2.88	Toll-like receptor signaling pathway
miR-10a-5p	+2.53	TLR7	−2.01	Toll-like receptor signaling pathway
miR-27b-3p	+4.84	PIK3CG	−2.77	Toll-like receptor signaling pathway
miR-27b-3p	+4.84	JAK2	−2.15	Toll-like receptor signaling pathway
miR-27b-3p	+4.84	DHX58	−4.95	RIG-I-like receptor signaling pathway
miR-30a-5p	+3.99	CXCL9	−3.93	Toll-like receptor signaling pathway
miR-30a-5p	+3.99	IL12B	−4.2	Toll-like receptor signaling pathway
miR-30a-5p	+3.99	IL12B	−4.2	RIG-I-like receptor signaling pathway
miR-30a-5p	+3.99	MAP3K1	−3.03	RIG-I-like receptor signaling pathway
miR-30a-5p	+3.99	TANK	−2.09	RIG-I-like receptor signaling pathway
miR-143-3p	+6.68	CD40	−4.97	Toll-like receptor signaling pathway
miR-143-3p	+6.68	JAK3	−2.69	Toll-like receptor signaling pathway
miR-143-3p	+6.68	DDX58	−4.44	RIG-I-like receptor signaling pathway
miR-143-3p	+6.68	IKBKB	−2.16	RIG-I-like receptor signaling pathway
miR-148a-3p	+2.46	MAPK14	−3.77	Toll-like receptor signaling pathway
miR-148a-3p	+2.46	STAT1	−2.67	Toll-like receptor signaling pathway
miR-148a-3p	+2.46	STAT5B	−2.55	Toll-like receptor signaling pathway
miR-148a-3p	+2.46	DDX3X	−2.94	RIG-I-like receptor signaling pathway
miR-novel-chr2_21624	+6.68	IKBKB	−2.16	Toll-like receptor signaling pathway
miR-novel-chr2_21624	+6.68	MAP3K7	−3.96	Toll-like receptor signaling pathway
miR-novel-chr2_21624	+6.68	MAP3K7	−3.96	RIG-I-like receptor signaling pathway
miR-novel-chr2_21624	+6.68	MAPK14	−3.77	RIG-I-like receptor signaling pathway

“+” and “–” indicate upregulated and downregulated miRNAs or mRNAs, respectively. The fold change cutoffs of the upregulated miRNAs and the downregulated miRNAs or mRNAs were +2 and −0.5, respectively

## Discussion

There are a range of immunosuppressive viruses that seriously damage human health and the aquaculture industry, including human immunodeficiency virus (HIV), PRRSV, porcine circovirus (PCV) and PCMV. Immunosuppressive viruses interfere with the immune processes of their animal hosts in multiple ways; for example, HIV causes immune dysfunction of the host by interfering with the function of CD4^+^ cytotoxic T lymphocytes, thus facilitating pathogen infection. Infection with human cytomegalovirus (HCMV) can then occur, which further harms the immunosuppressed host [25–29].

Similar to the coinfection of humans with immunosuppressive viruses, the infection of animals with immunosuppressive viruses, followed by secondary viral and bacterial infections, can cause serious harm and inhibit disease prevention and treatment. In a previous study in Sichuan Province, China, we observed that the PCMV and PRRSV coinfection rate in pig was 34.7%, while the PCMV/PRRSV/*Thermoproteus tenax* virus 1/pseudorabies virus coinfection rate was 14.9% [30].

Therefore, we hypothesised that the coinfection of PRRSV/PCMV has an immunosuppressive mechanism similar to that of HIV/HCMV coinfection. Although our previous study confirmed that PCMV suppresses the immune function of porcine central immune organs, there has been little research on the molecular mechanism behind PCMV immunosuppression. The miRNA transcriptome of porcine continuous cell lines was previously reported, but there have been no reports on the miRNA transcriptome of PCMV-infected vital organs [22, 30, 31].

In this study, 430, 382, 433, 324 and 477 annotated and novel miRNAs were identified in PCMV-infected and uninfected porcine lungs, liver, spleen, kidneys and thymus, respectively, using small-RNA transcriptome high-throughput sequencing technology. The potential regulatory roles of DE miRNAs on their target genes were also predicted. Unfortunately, no PCMV-encoded miRNAs were found in the 10 small-RNA expression profiles of porcine organs.

From the high-throughput sequencing data, the length distribution of small-RNA reads in the 10 expression profiles was consistent with the results obtained in other mammals [32, 33], which suggests that small-RNA expression is conserved among different mammals. The percentages of small-RNA (rRNA, tRNA, sRNA, miRNA and snRNA) expression were also significantly changed in PCMV-infected organs compared with those in uninfected ones; however, the biological significance of these changes of small RNAs under the pressure of viral infection requires further study (Additional file 1: Figure S2).

According to the high-throughput sequencing data, the let-7 family (let-7a, let-7f, let-7 g), the miR-10 family (miR-10b, miR-10a-3p, miR-10a-5p), miR-21, miR-143-3p, miR-30a-5p, miR-16 and miR-192 had the highest expression levels among the 10 expression profiles (Additional file 1: Figure S3), which suggests that these miRNAs are highly conserved among different organs in the same species. These miRNAs also exhibit a high expression level in other mammals, which indicates that they are likely to play an important role in biological processes in these animals [34–36].

By a comparison with previous studies [37, 38], we observed that a series of specific host miRNAs were generally differentially expressed under the pressure of viral infection in vivo and in vitro (Additional file 1: Table S6). As such, we assume that PCMV interferes with the function of the immune system by altering the expression levels of specific miRNAs, thereby facilitating viral infection.

In this study, miR-10a-3p was generally significantly upregulated in PCMV-infected lung (278% increase), liver (200% increase), thymus (153% increase) and kidney (2.53% increase). miR-155-5p was also upregulated in PCMV-infected lung (526% increase), liver (239% increase) and spleen (493% increase) compared with that in the uninfected control. Recent reports have shown that miR-10a inhibits the immune responses of Th1/Th17 cells and dendritic cell activation by targeting IL-12/IL-23p40. miR-10a has also been determined to be negatively regulated by microbes, thereby promoting the maintenance of intestinal homeostasis in the host [39, 40]. In addition, a previous study showed that miR-155 plays an important role in antimicrobial infection. The expression level of miR-155 was also found to be increased during host antiviral responses; for example, it was significantly upregulated in EBV-infected lymphocytes and interfered with the balance of gene expression by modulating transcriptional regulatory factors [41–45].

Our recent studies showed that miR-10a-3p is upregulated in PCMV-infected porcine macrophages and Japanese encephalitis virus-infected porcine kidney epithelial cells (PK-15). Meanwhile, miR-155 was found to be significantly upregulated in PCMV-infected porcine macrophages and porcine parvovirus-infected PK-15 cells (Additional file 1: Table S5). Therefore, we hypothesised that these miRNAs play similar roles in the response of organisms to infection with multiple pathogens [37, 46].

As other examples of miRNAs that miR-7d-5p, miR-301 and miR-421-3p were significantly downregulated in PCMV-infected lungs and liver compared with their levels in uninfected samples, suggesting that they are involved in the response to viral infection in these organs. However, the regulatory mechanisms of these miRNAs require further study.

miRNAs regulate target genes by inhibiting mRNA translation or degrading their target mRNAs. The DE miRNAs and target mRNAs identified in the integrated expression analysis of PCMV-infected porcine immune organ in the present study revealed the interactions of these miRNAs and the biological processes with which the target genes are involved (Additional file 1: Table S5). The results showed that the expression levels of DE miRNAs and those of their predicted target genes were strongly correlated; specifically, increased expression of DE miRNAs led to lower levels of expression of their target genes.

Studies have shown that TLRs and RLRs are the major pattern recognition receptors of hosts. Host cells regulate TLR and RLR signalling pathways in multiple ways, thus ensuring the homeostasis of signal transduction. Previous studies showed that hepatitis C virus (HCV), poliovirus and rotavirus inhibit TLR and RLR signalling pathways by escaping recognition by TLRs and RLRs, degrading key signalling molecules, regulating the modification of signal molecules and blocking signal transduction, thereby facilitating viral infection [47–55].

The results of our recent study also showed that the TLR and RLR signalling pathways were significantly inhibited in PCMV-infected thymus [22]. In this study, the results of integrated expression analysis showed that miR-143-3p, miR-30a-5p, miR-novel-chr2_21624, miR-148a-3p, miR-27b-3p and miR-10a-5p were significantly upregulated in PCMV-infected thymus, while the expression of their target genes, which are related to the TLR and RLR signalling pathways, were significantly downregulated (Table 2). This suggests that PCMV infection inhibits the expression of genes that are crucial for immune-related signalling pathways by promoting the expression of specific miRNAs, thus promoting to viral infection.

This is the first comprehensive analysis of the responses of host small-RNA transcriptomes to PCMV infection in vital porcine organs. It provides new insights into the regulatory mechanisms of miRNAs during infection by immunosuppressive viruses.

## Conclusion

This is the first report on the expression profiles of small RNA in response to PCMV infection in vital porcine organs. A total of 306 annotated and 295 novel miRNAs were identified from PCMV-infected and uninfected porcine lungs, liver, spleen, kidneys and thymus. GO annotation analysis of the target genes of DE miRNAs showed that the DE miRNAs in PCMV-infected samples mainly participate in immune system processes, regulation of biological processes, cellular processes and metabolic processes, among others. The results of integrated expression analysis of DE miRNAs and their target mRNAs in PCMV-infected central immune organs indicated that PCMV interferes with crucial immune-related signalling pathways by regulating the expression of specific miRNAs, thus promoting the viral infection.

## Additional file

Additional file 1: **Figure S1.** Length distribution of total sRNAs. **Figure S2.** Pie charts of sRNA percentages. **Figure S3.** Top 10 miRNA reads. **Figure S4.** GO functional enrichment annotations for the target genes of DE miRNAs. **Table S1.** Overview of miRNA high-throughput sequencing data. **Table S2.** Expression profiles of miRNAs. **Table S3.** Differentially expressed miRNAs. **Table S4.** Target prediction for DE miRNAs. **Table S5.** Integrated expression analysis of DE miRNAs and their target mRNA. **Table S6.** DE miRNAs in vivo and vitro under viral infection. **Table S7.** Primers used for stem-loop qRT-PCR. (DOCX 2983 kb)

## Abbreviations

APOA1: Apolipoprotein A-I
CCR1: Chemokine (C-C motif) receptor 1
CXCR4: Chemokine (C-X-C motif) receptor 4
DE: Differentially expressed
EBV: Epstein–Barr viru
GO: Gene Ontology
HCMV: Human cytomegalovirus
HIV: Human immunodeficiency virus
IL1α: Interleukin-1α
IRAK4: Interleukin-1 receptor-associated kinase 4
KLHL6: Kelch-like protein6
miRNAs: MicroRNAs
ncRNA: Non-coding RNA
Nt: Nucleotide
PCMV: Porcine cytomegalovirus
PCR: Polymerase chain reaction
PK-15: Porcine kidney epithelial cells
PRR2: Pinoresinol reductase 2
PRRSV: Porcine reproductive and respiratory syndrome virus
RLR: RIG-I-like receptor
rRNA: Ribosomal RN
snRNA: Small nuclear RNA
sRNA: Small RNA
STAT5B: Signal transducer and activator of transcription 5B
STRING: Search tool for the retrieval of interacting genes/proteins
TLR1: Toll-like receptor 1
TNF: Tumor necrosis factor
tRNA: Transfer RNA
UCSC: University of California Santa Cruz
