# Supplementary material for: MicroRNA transcriptome analysis of porcine vital organ responses to immunosuppressive porcine cytomegalovirus infection
Source: Virol J. 2018 Jan 18;15:16. doi: 10.1186/s12985-018-0922-x (PMC5774105; doi:10.1186/s12985-018-0922-x)
Supplement: Additional file 1: — Figure S1. Length distribution of total sRNAs. Figure S2. Pie charts of sRNA percentages. Figure S3. Top 10 miRNA reads. Figure S4. GO functional enrichment annotations for the target genes of DE miRNAs. Table S1. Overview of miRNA high-throughput sequencing data. Table S2. Expression profiles of miRNAs. Table S3. Differentially expressed miRNAs. Table S4. Target prediction for DE miRNAs. Table S5. Integrated expression analysis of DE miRNAs and their target mRNA. Table S6. DE miRNAs in vivo and vitro under viral infection. Table S7. Primers used for stem-loop qRT-PCR. (DOCX 2983 kb) [file 12985_2018_922_MOESM1_ESM.docx]

**MicroRNA transcriptome analysis of porcine vital organ responses to immunosuppressive porcine cytomegalovirus infection**

**Xiao** **Liu** ^1,2^**, Haoche Wei** ^3^**, Shan Liao** ^1,2^**, Jianheng Ye** ^4^**, Ling Zhu** ^2^**, Zhiwen Xu** ^2 *^

^1^Southwest University, College of Animal Science and technology, 400715, Chongqing, China

^2^ Animal Biotechnology Center, College of Veterinary Medicine, Sichuan Agricultural University, Chengdu, 610000, China

^3^ College of Life Sciences, Sichuan University, Chengdu, 610000, China

^4^ Department of Urology, Guangdong Key Laboratory of Clinical Molecular Medicine and Diagnostics, Guangzhou First People's Hospital, Guangzhou Medical University, Guangzhou 510180, China

^*^Corresponding author at: Key Laboratory of Animal Disease and Human Health of Sichuan Province and Animal Biotechnology Center, College of Veterinary Medicine of Sichuan Agricultural University, 211#Huimin Road, Wenjiang District, Chengdu 610000, Sichuan Province, China. Tel.: +86 835 2885846; fax: +86 835 2885302; E-mail address: [abtcxzw@126.com](mailto:abtcxzw@126.com) (Zhiwen Xu).

**Additiona file**

**Figure S1. Length distribution of total sRNAs.**

**Figure S2. Pie charts of sRNA percentages.**

**Figure S3. Top 10 miRNA reads.**

**Figure S4. GO functional enrichment annotations for the target genes of DE miRNAs.**

**Table S1. Overview of miRNA high-throughput sequencing data**

**Table S2. Expression profiles of miRNAs.**

**Table S3. Differentially expressed miRNAs.**

**Table S4. Target prediction for DE miRNAs.**

**Table S5. Integrated expression analysis of DE miRNAs and their target mRNA**

**Table S6. DE miRNAs in vivo and vitro under viral infection**

**Table S7. Primers used for stem-loop qRT-PCR.**

**Figure S1. Length distribution of total sRNAs.**

(a–e) Read counts against the read lengths for the complete adapter-trimmed read set in uninfected lung, liver, spleen, kidney and thymus samples.

(f–j) Read counts against the read lengths for the complete adapter-trimmed read set in PCMV-infected lung, liver, spleen, kidney and thymus samples.

**
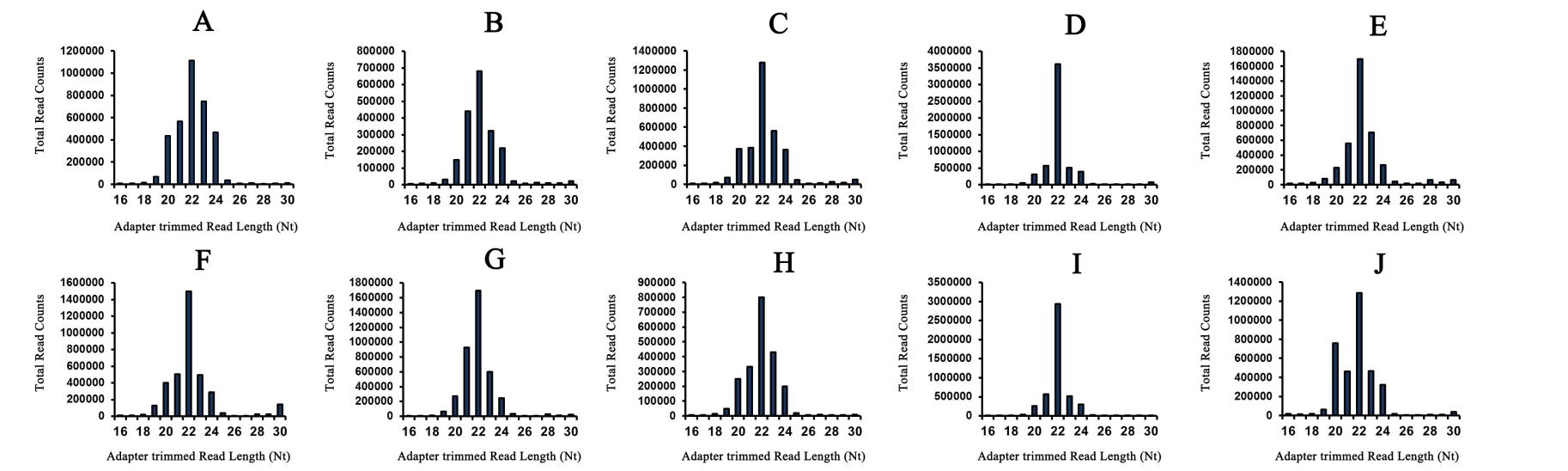
**

**Figure S2. Pie charts of sRNA percentages.** (A–E) Different classes of sRNAs (miRNA, tRNA, snoRNA, snRNA and rRNA) in uninfected lung, liver, spleen, kidney and thymus samples. (F–J) Different classes of sRNAs (miRNA, tRNA, snoRNA, snRNA and rRNA) in PCMV-infected lung, liver, spleen, kidney and thymus samples.

**
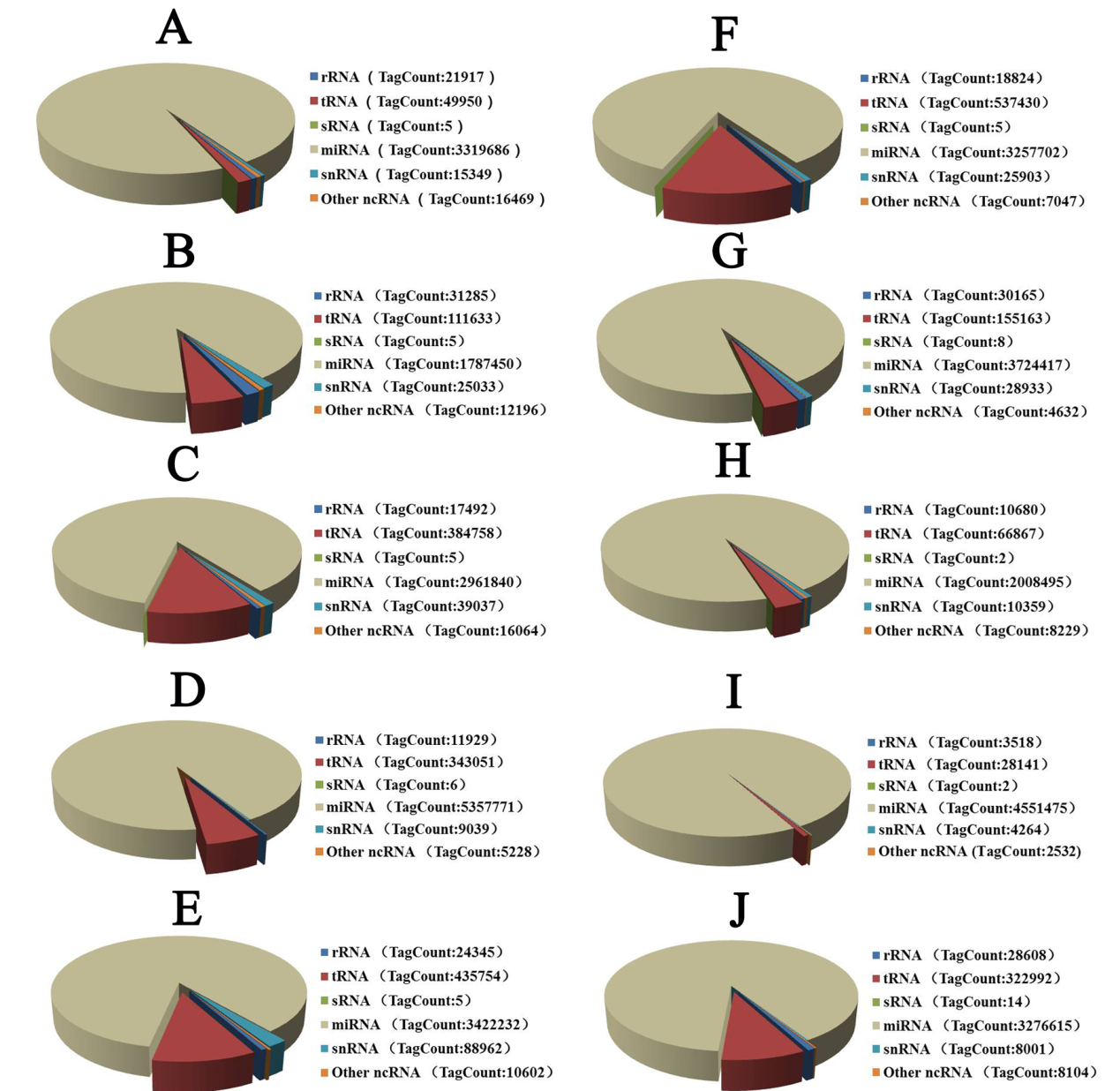
**

**Figure S3. Top 10 miRNA reads.** (A)-(E) Top 10 miRNA reads of non-infected lung, liver, spleen, kidney and thymus samples. (F)-(J) Top 10 miRNA reads of PCMV infected lung, liver, spleen, kidney and thymus samples

**A F**


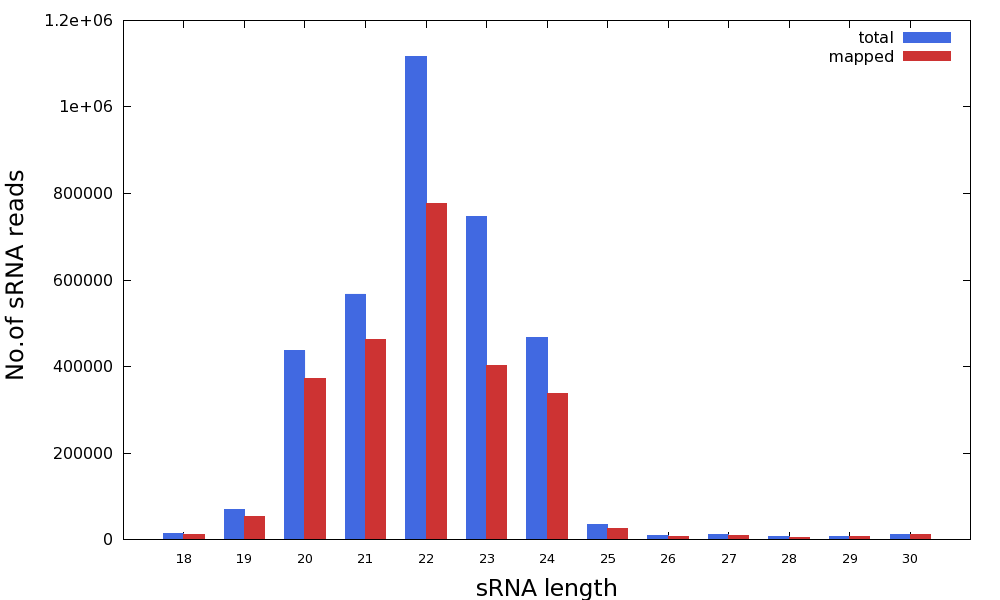

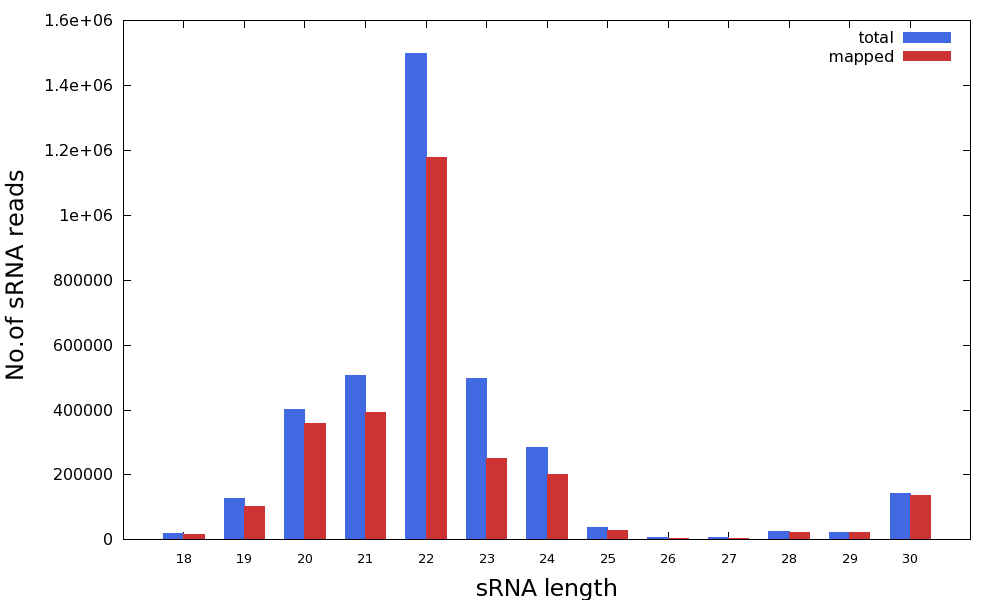


**B I**


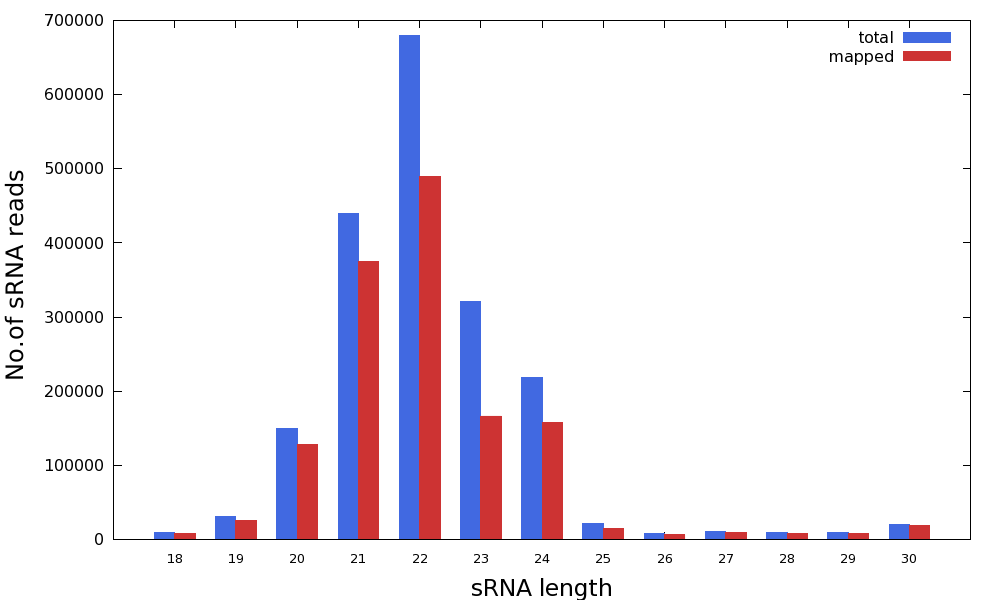

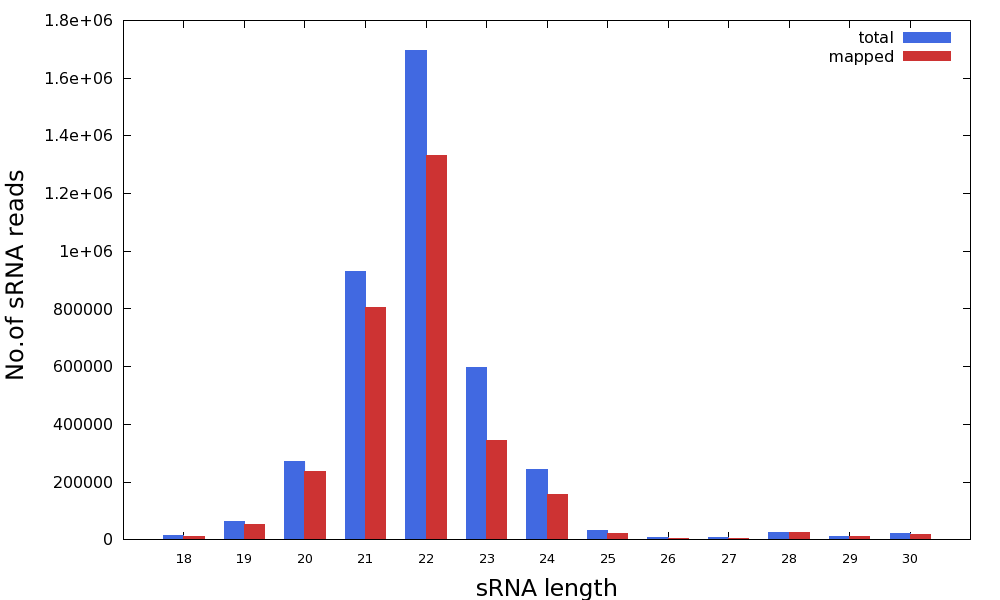


**C J**


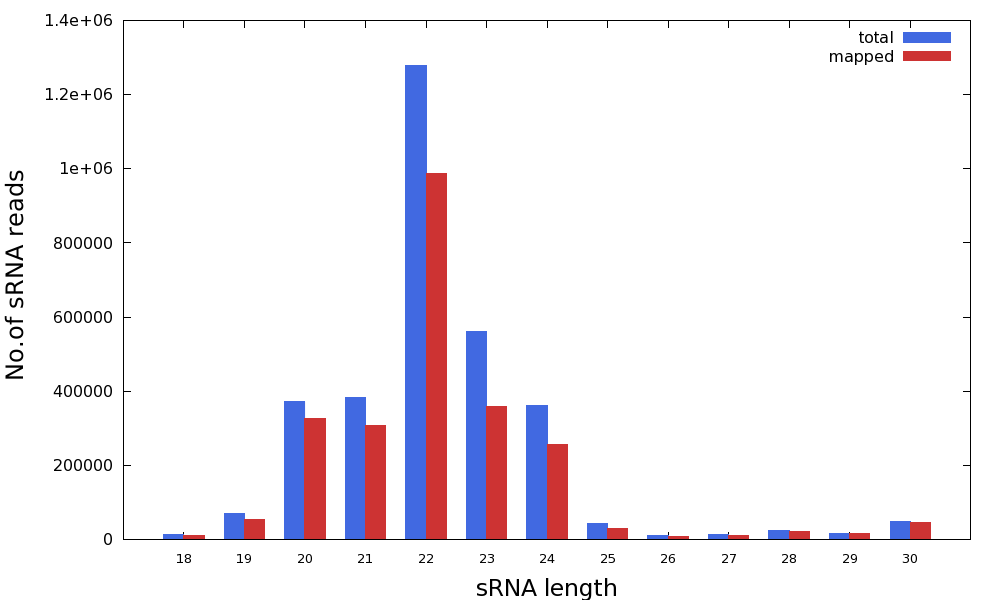

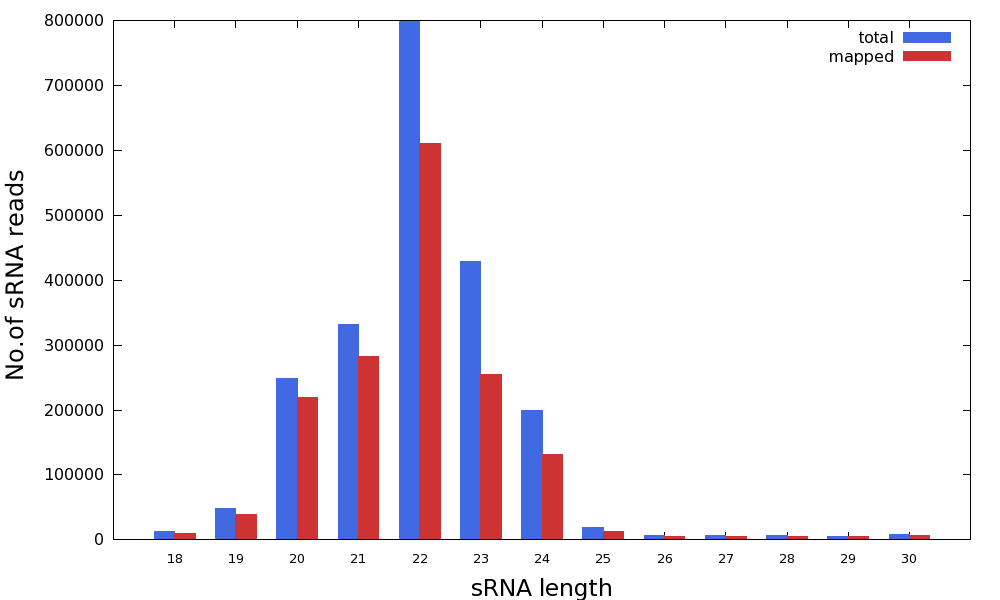


**D K**


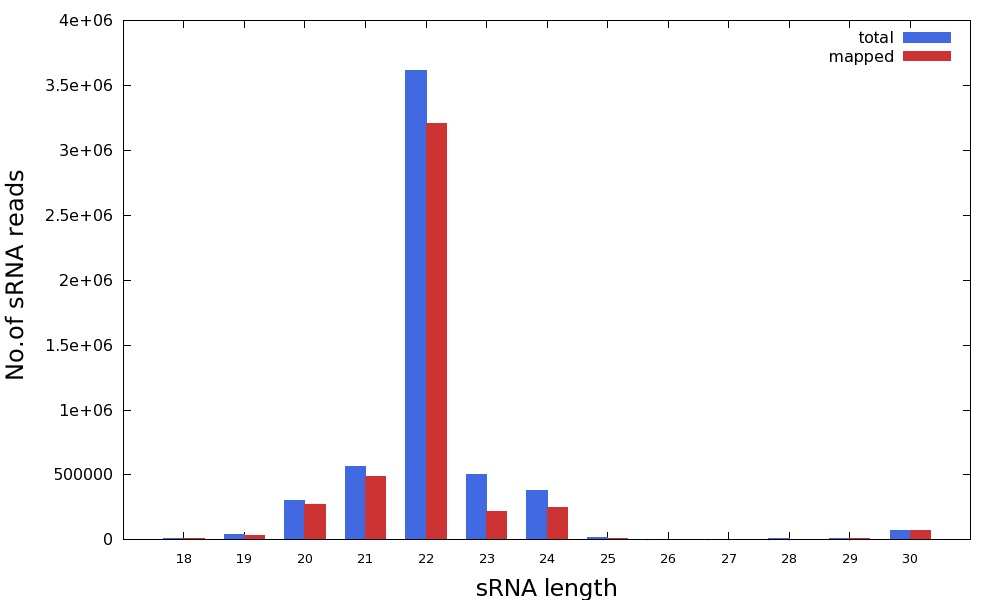

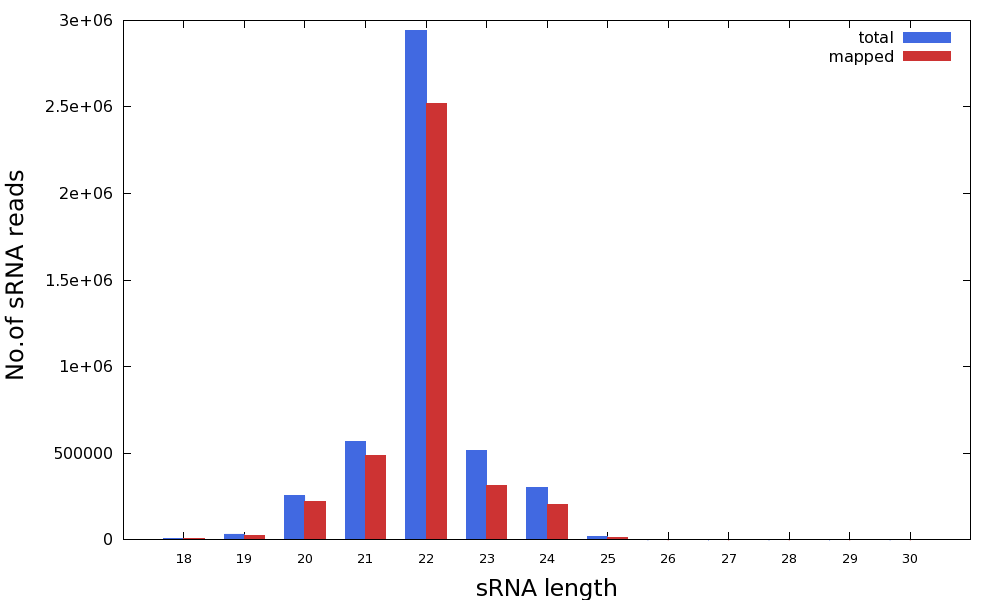


**E L**


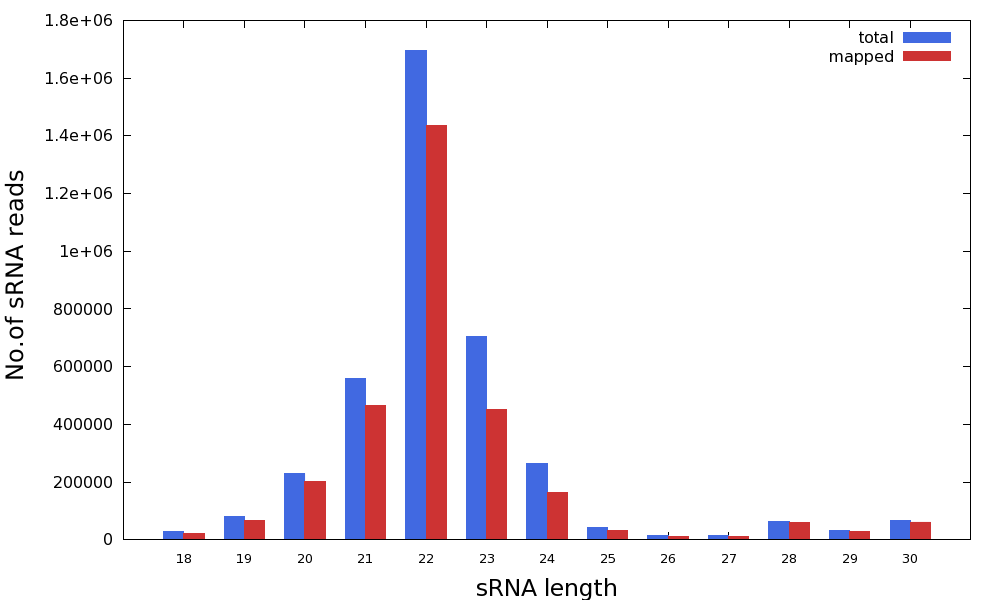

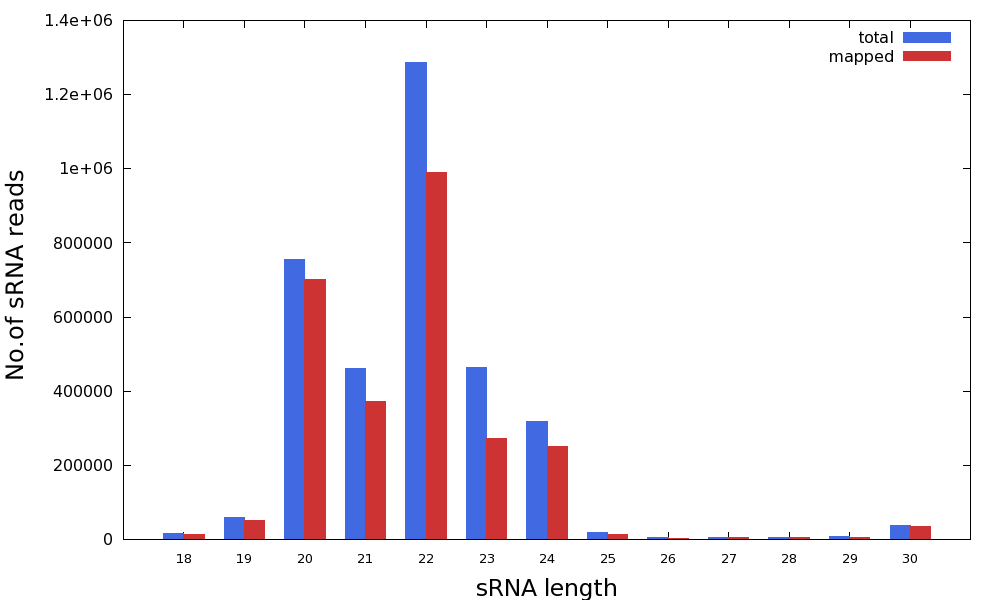


**Figure S4. GO functional enrichment annotations for the target genes of DE miRNAs.**

GO terms of up-regulated miRNAs target genes in PCMV infected lung (Biological Process)


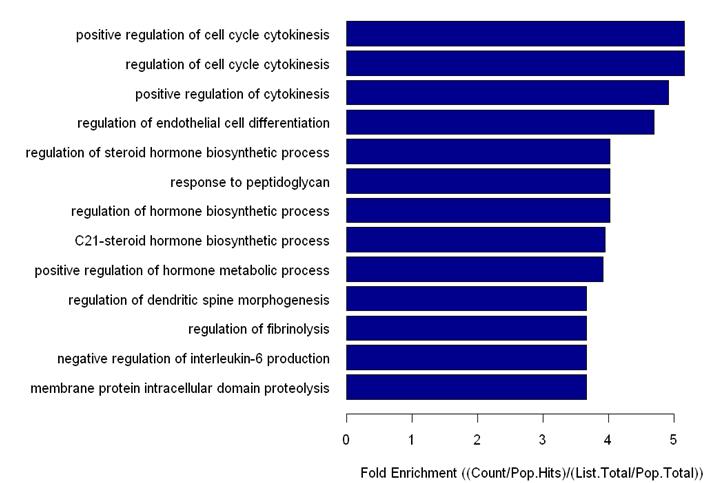


GO terms of up-regulated miRNAs target genes in PCMV infected lung (Cellular Component)


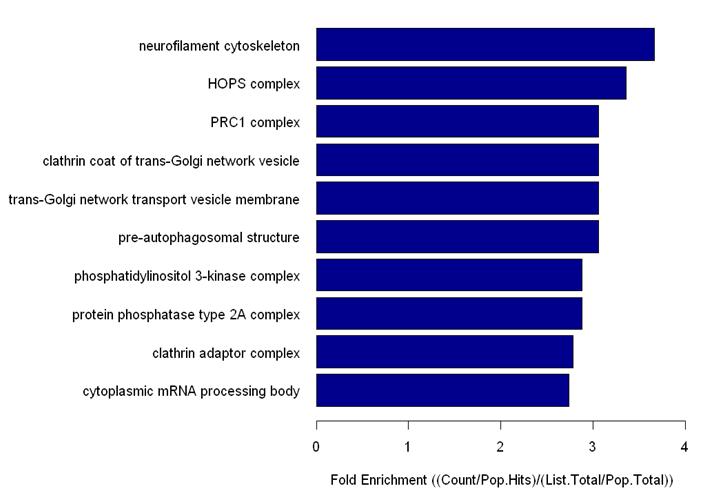


GO terms of up-regulated miRNAs target genes in PCMV infected lung (Molecular Function)

**
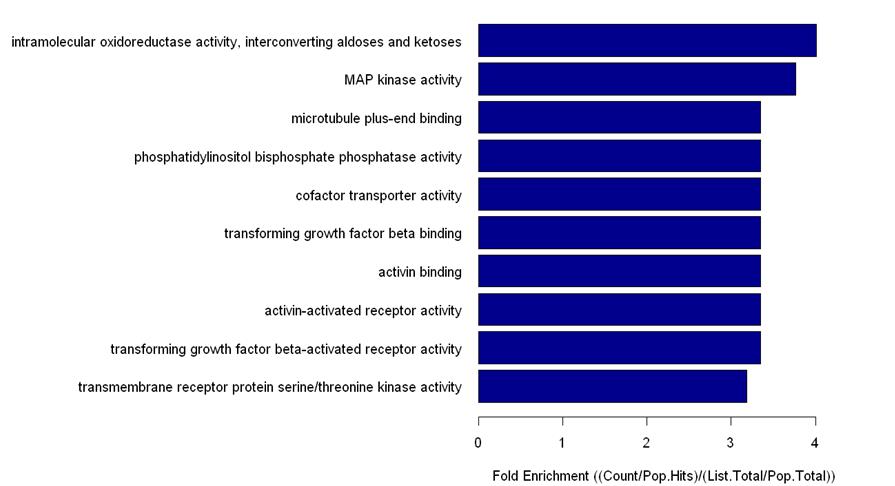
**

GO terms of down-regulated miRNAs target genes in PCMV infected lung (Biological Process)

**
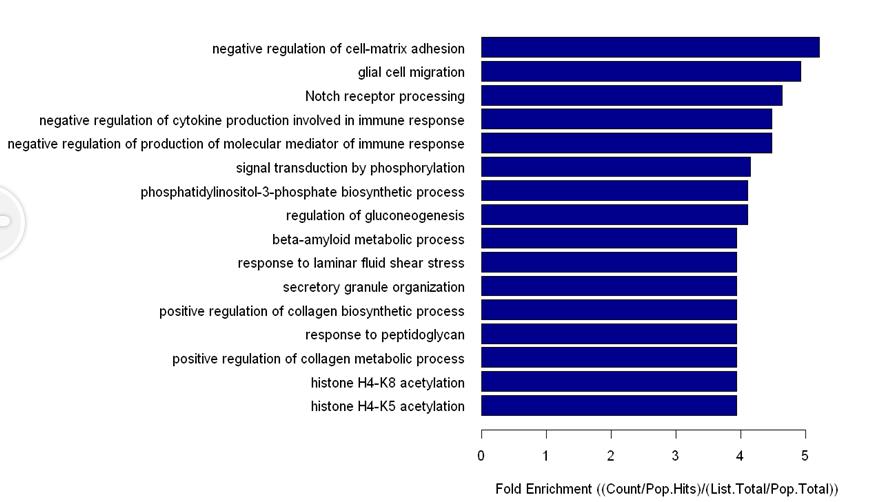
**

GO terms of down-regulated miRNAs target genes in PCMV infected lung (Cellular Component)

**
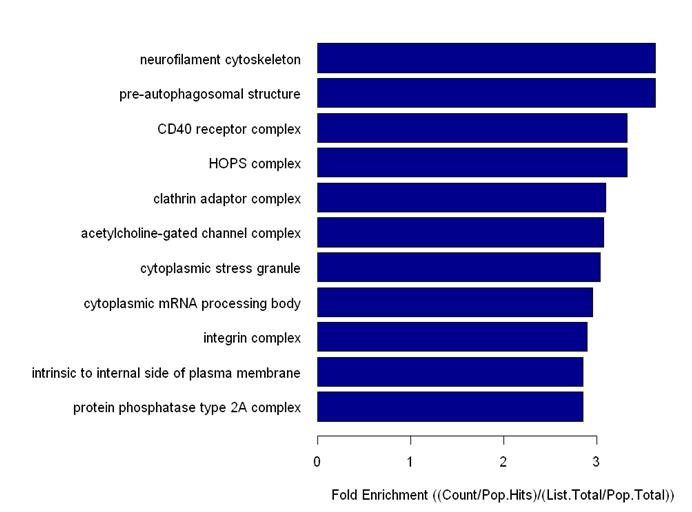
**

GO terms of down-regulated miRNAs target genes in PCMV infected lung (Molecular Function)

**
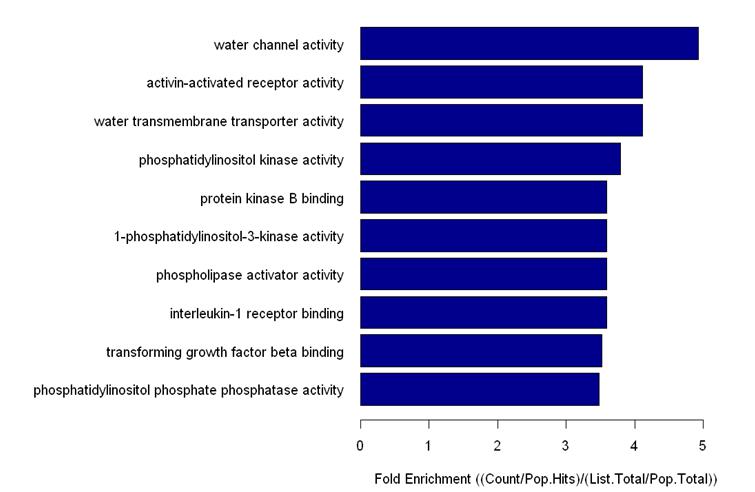
**

GO terms of up-regulated miRNAs target genes in PCMV infected liver (Biological Process)

**
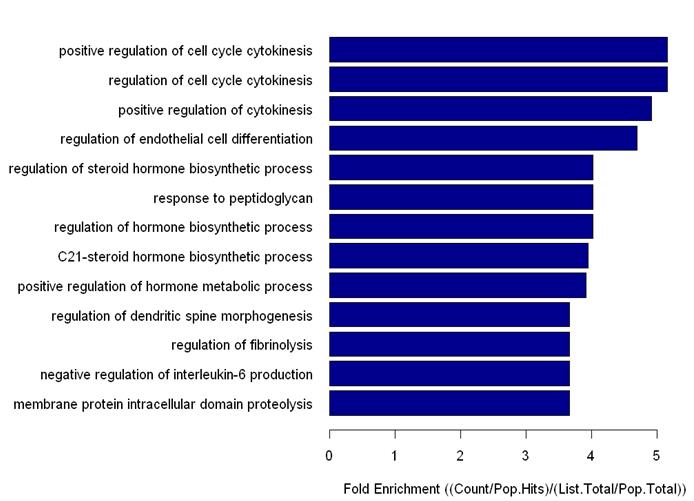
**

GO terms of up-regulated miRNAs target genes in PCMV infected liver (Cellular Component)

**
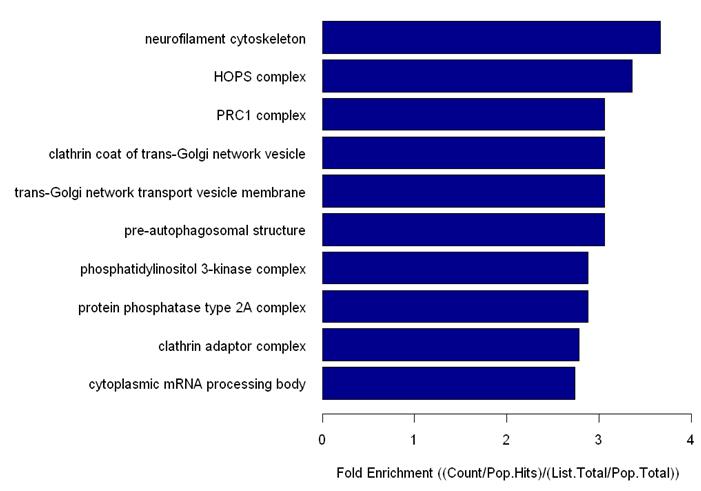
**

GO terms of up-regulated miRNAs target genes in PCMV infected liver (Molecular Function)

**
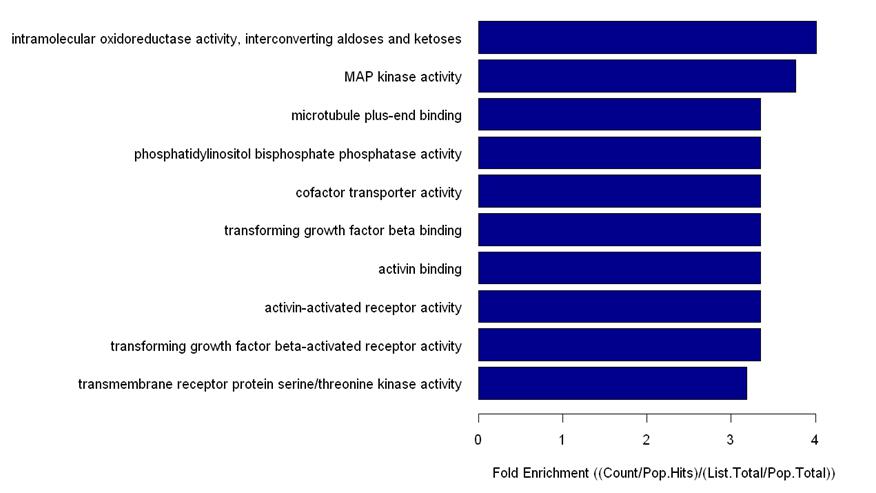
**

GO terms of down-regulated miRNAs target genes in PCMV infected liver (Biological Process)

**
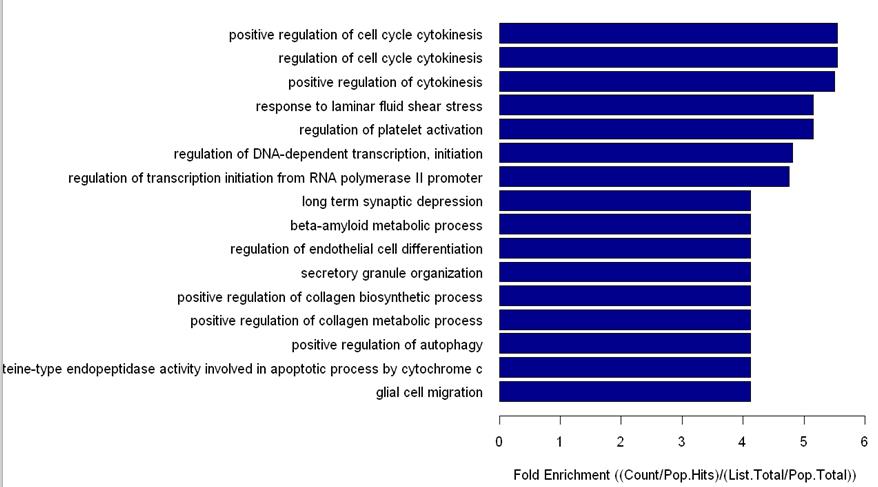
**

GO terms of down-regulated miRNAs target genes in PCMV infected liver (Cellular Component)

**
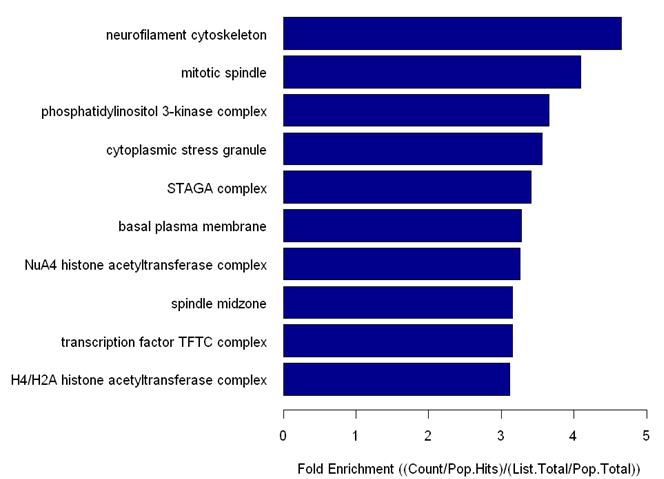
**

GO terms of down-regulated miRNAs target genes in PCMV infected liver (Molecular Function)

**
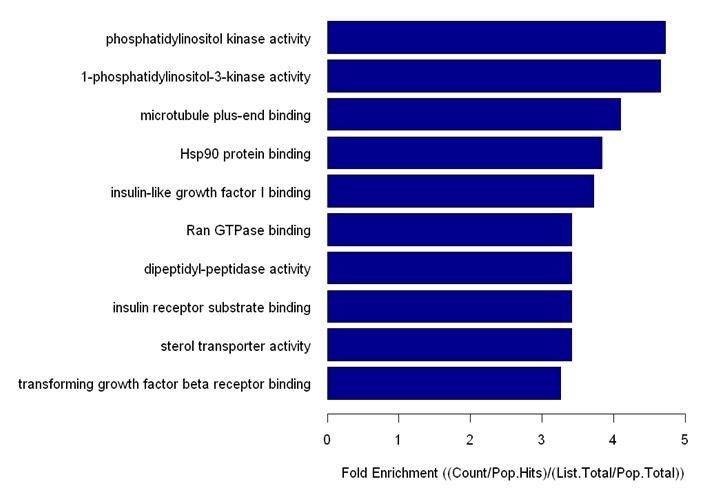
**

GO terms of up-regulated miRNAs target genes in PCMV infected spleen (Biological Process)

**
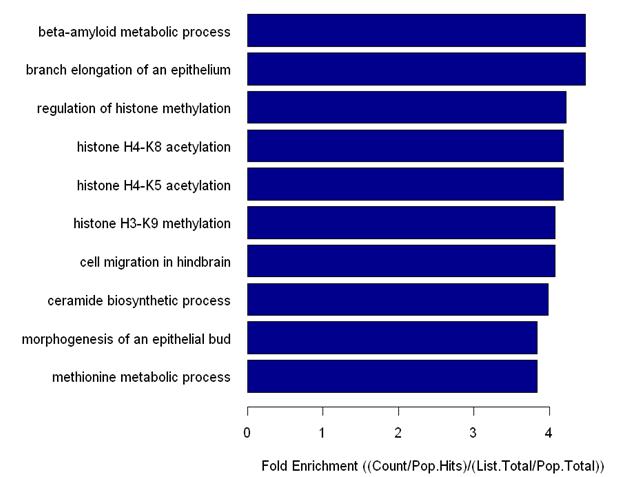
**

GO terms of up-regulated miRNAs target genes in PCMV infected spleen (Cellular Component)

**
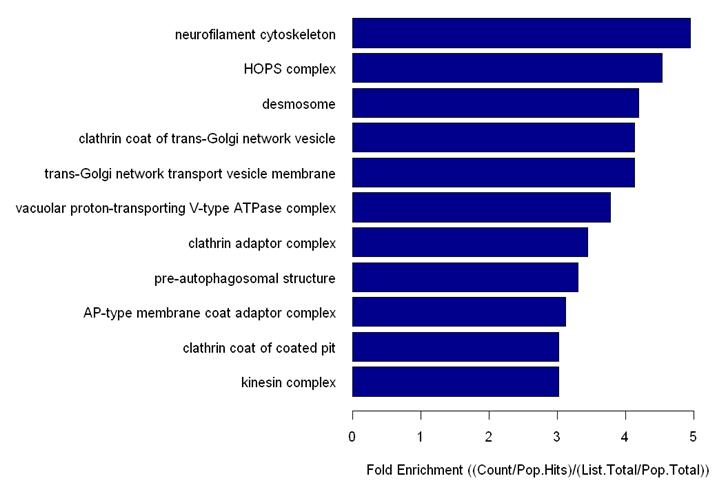
**

GO terms of up-regulated miRNAs target genes in PCMV infected spleen (Molecular Function)

**
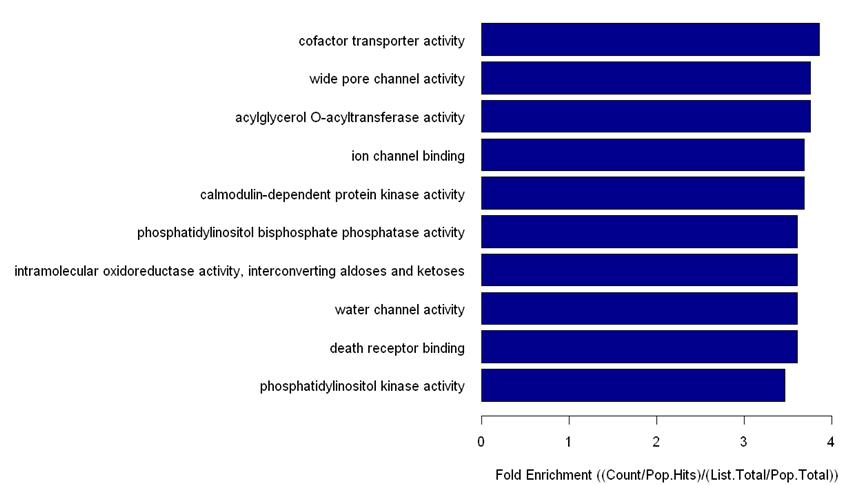
**

GO terms of down-regulated miRNAs target genes in PCMV infected spleen (Biological Process)

**
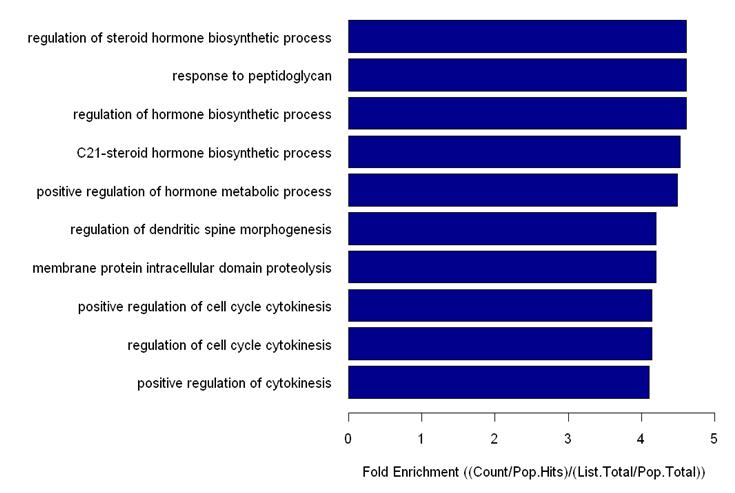
**

GO terms of down-regulated miRNAs target genes in PCMV infected spleen (Cellular Component)

**
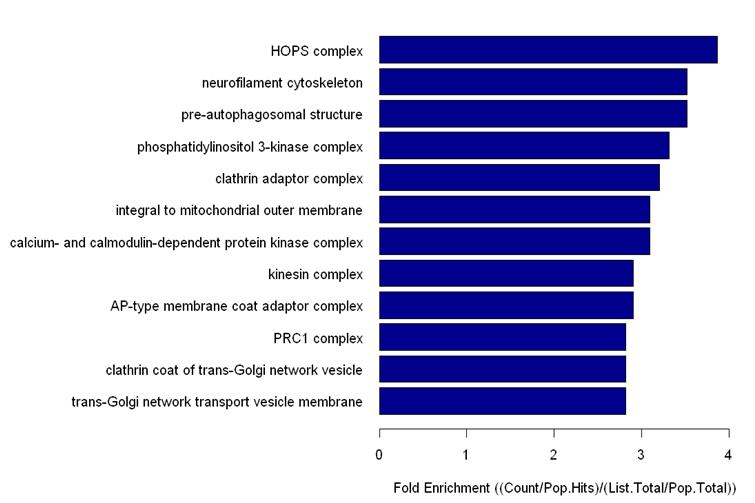
**

GO terms of down-regulated miRNAs target genes in PCMV infected spleen (Molecular Function)

**
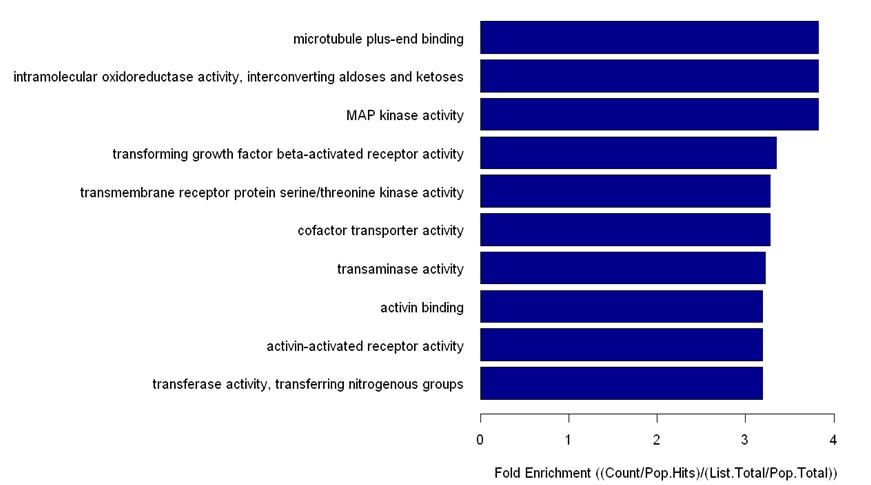
**

GO terms of up-regulated miRNAs target genes in PCMV infected kidney (Biological Process)

**
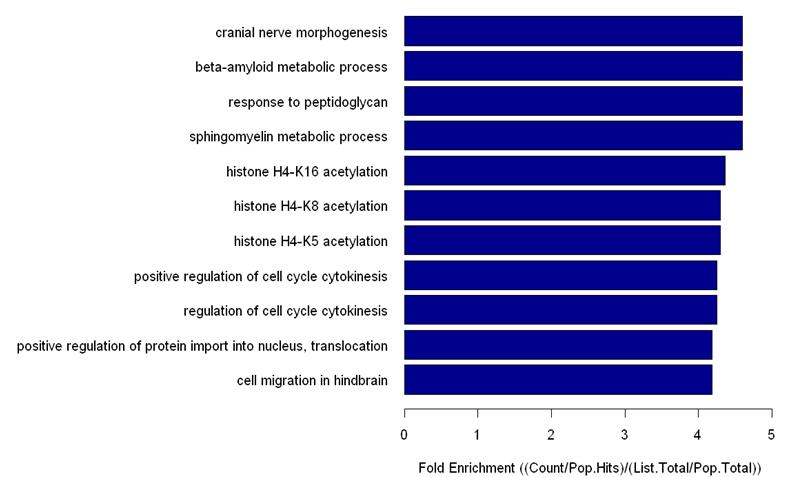
**

GO terms of up-regulated miRNAs target genes in PCMV infected kidney (Cellular Component)

**
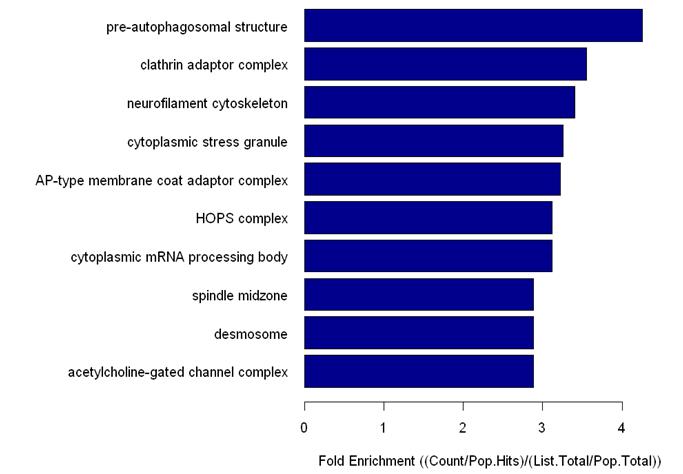
**

GO terms of up-regulated miRNAs target genes in PCMV infected kidney (Molecular Function)

**
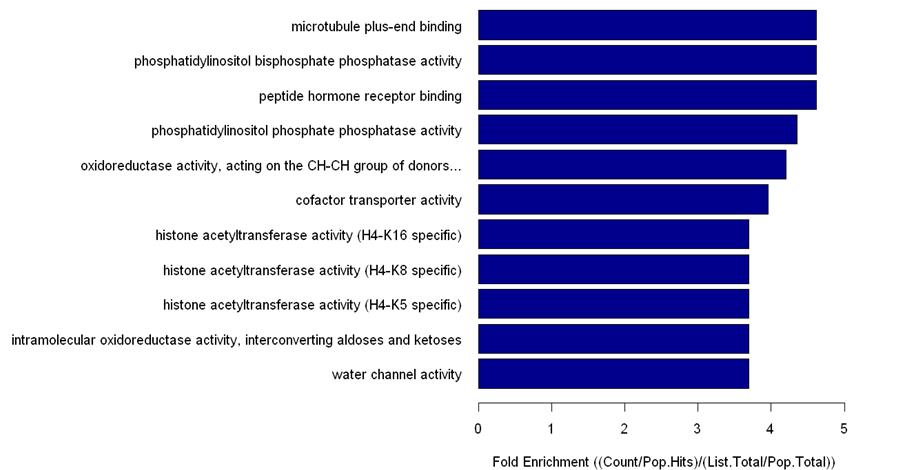
**

GO terms of down-regulated miRNAs target genes in PCMV infected kidney (Biological Process)

**
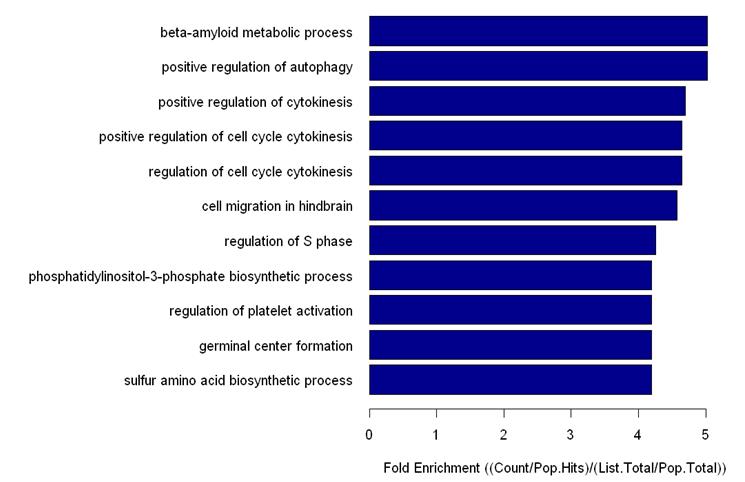
**

GO terms of down-regulated miRNAs target genes in PCMV infected kidney (Cellular Component)

**
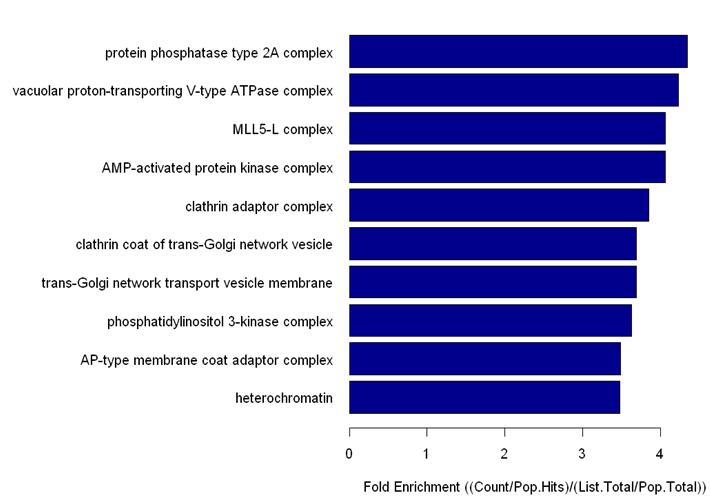
**

GO terms of down-regulated miRNAs target genes in PCMV infected kidney (Molecular Function)

**
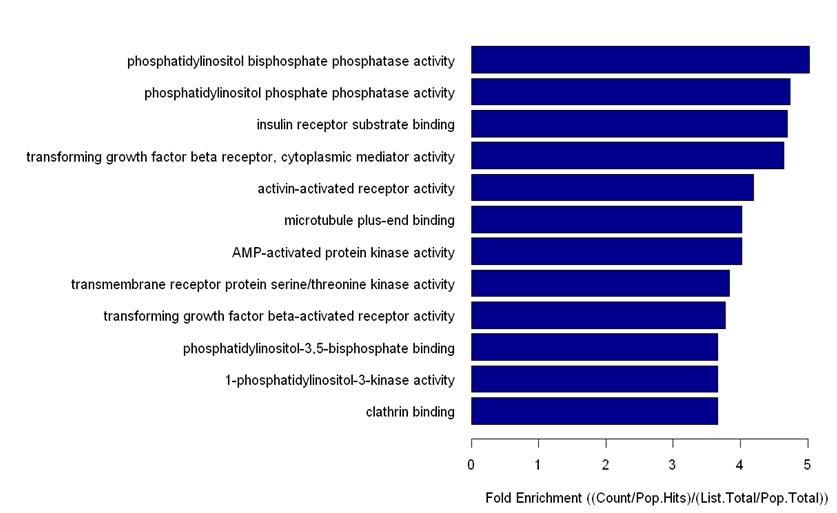
**

GO terms of up-regulated miRNAs target genes in PCMV infected thymus (Biological Process)

**
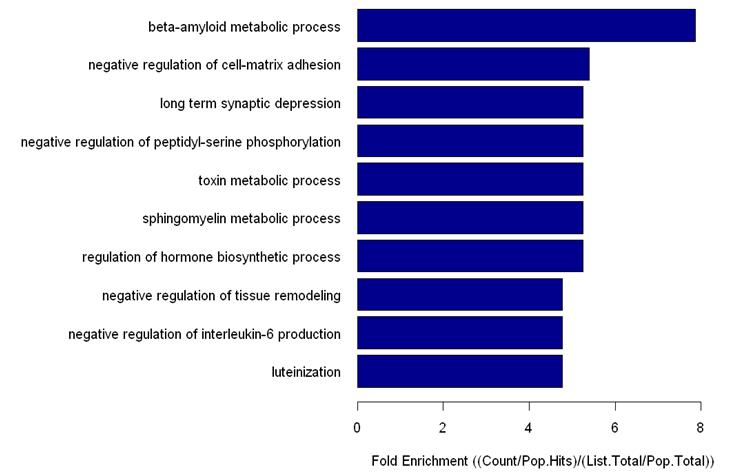
**

GO terms of up-regulated miRNAs target genes in PCMV infected thymus (Cellular Component)

**
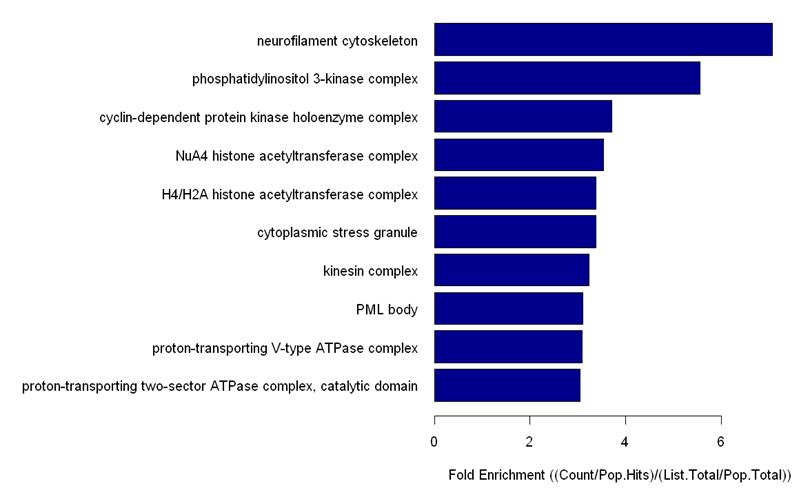
**

GO terms of up-regulated miRNAs target genes in PCMV infected thymus (Molecular Function)

**
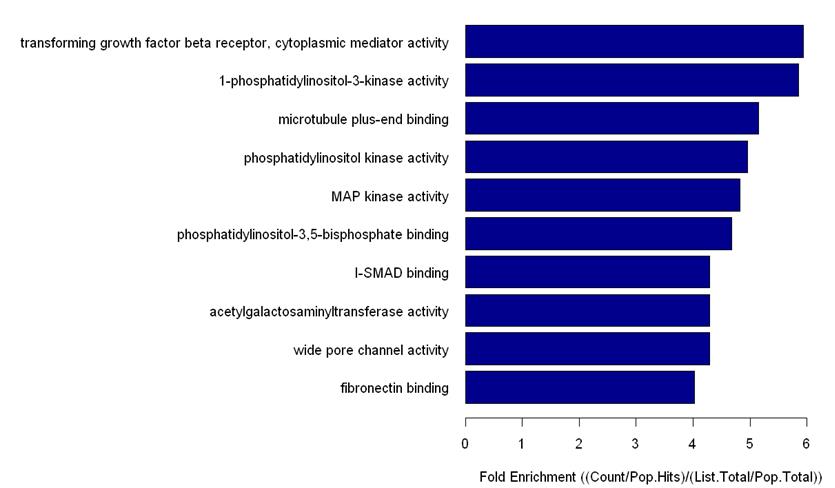
**

GO terms of down-regulated miRNAs target genes in PCMV infected thymus (Biological Process)

**
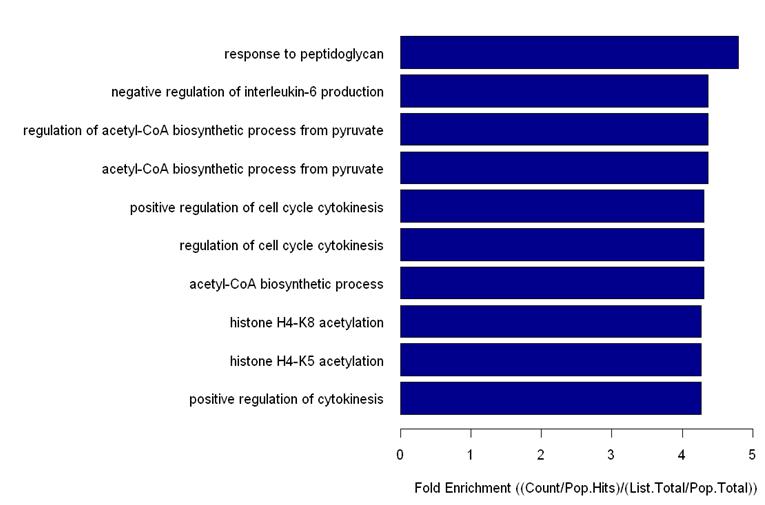
**

GO terms of down-regulated miRNAs target genes in PCMV infected thymus (Cellular Component)

**
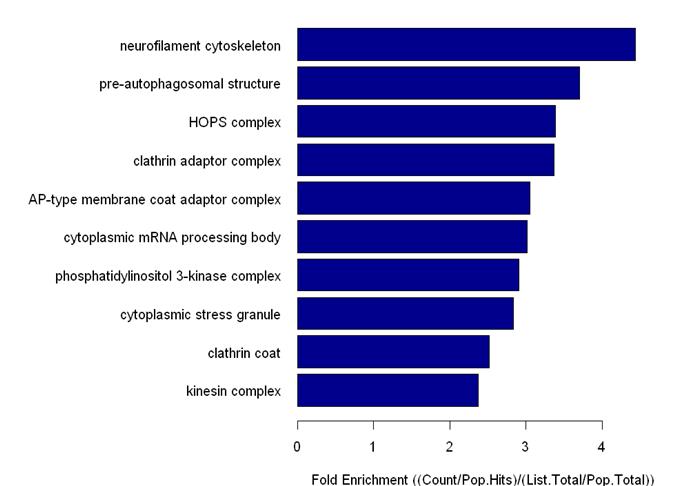
**

GO terms of down-regulated miRNAs target genes in PCMV infected thymus (Molecular Function)

**
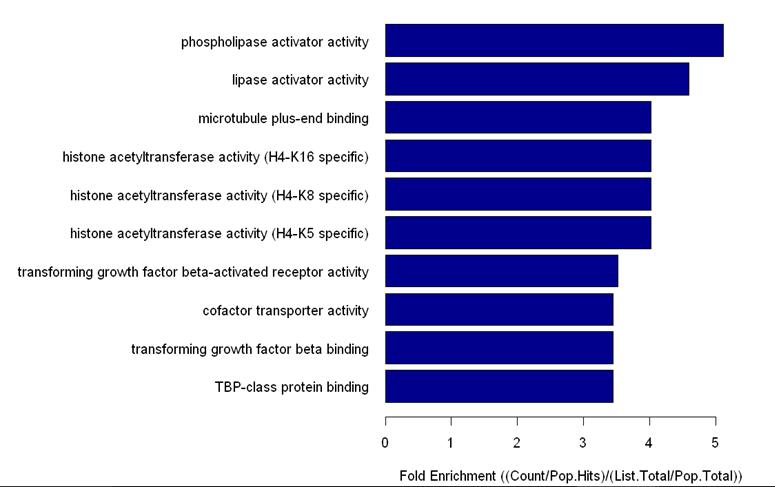
**

**Table S1. Overview of miRNA high-throughput sequencing data**

The number of reads aligned to the known *Sus scrofa* pre-miRNAs in miRBase 21 with ≤1 mismatch.

| **Sample Name** | **Clean Reads** | **Adapter-trimmed Reads (length >= 15nt)** | **Reads aligned to known *Sus scrofa* pre-miRNA in miRBase 19** |
| --- | --- | --- | --- |
| Lung-Control | 3,832,388 | 3,707,104 | 3,269,785 |
| Thymus-Control | 4,466,024 | 4,354,092 | 3,358,408 |
| Kidney-Control | 5,956,114 | 5,919,279 | 5,328,931 |
| Spleen-Control | 3,769,006 | 3,678,250 | 2,923,738 |
| Liver-Control | 2,238,583 | 2,153,420 | 1,766,005 |
| PCMV infected Lung | 4,114,858 | 4,064,287 | 3,189,473 |
| PCMV infected Thymus | 3,880,070 | 3,822,201 | 3,214,411 |
| PCMV infected Kidney | 4,813,485 | 4,775,955 | 4,526,915 |
| PCMV infected Spleen | 2,326,036 | 2,266,938 | 1,977,364 |
| PCMV infected Liver | 4,291,107 | 4,246,899 | 3,694,737 |

The number of reads aligned to the known Sus scrofa pre-miRNAs in miRBase 21 with <1 mismatch, respectively.

**Table S2. Expression profiles of miRNAs.**

| **MATURE-ID** | **Non-infected thymus** | **PCMV infected thymus** | **Non-infected kidney** | **PCMV infected kidney** | **Non-infected liver** | **PCMV infected liver** | **Non-infected lung** | **PCMV infected lung** | **Non-infected spleen** | **PCMV infected spleen** |
| --- | --- | --- | --- | --- | --- | --- | --- | --- | --- | --- |
| **ssc-let-7a** | **12010** | **14235** | **4242** | **1495** | **29274** | **7513** | **28635** | **14467** | **6553** | **17627** |
| **ssc-let-7a** | **12010** | **14235** | **4242** | **1495** | **29274** | **7513** | **28635** | **14467** | **6553** | **17627** |
| **ssc-let-7c** | **1545** | **2549** | **489** | **144** | **2226** | **942** | **6199** | **3504** | **918** | **2388** |
| **ssc-let-7d-5p** | **163** | **112** | **49** | **42** | **1194** | **56** | **1084** | **118** | **141** | **885** |
| **ssc-let-7d-3p** |  | **1** | **1** |  |  |  | **1** |  |  |  |
| **ssc-let-7e** | **315** | **769** | **346** | **96** | **527** | **207** | **832** | **609** | **309** | **392** |
| **ssc-let-7f** | **24801** | **14959** | **7432** | **3457** | **47250** | **11968** | **32534** | **15185** | **13734** | **38083** |
| **ssc-let-7f** | **24801** | **14959** | **7432** | **3457** | **47250** | **11968** | **32534** | **15185** | **13734** | **38083** |
| **ssc-let-7g** | **10112** | **3861** | **1611** | **2090** | **18351** | **4722** | **11773** | **6812** | **6551** | **10538** |
| **ssc-let-7i** | **6076** | **5433** | **1297** | **1114** | **3018** | **2764** | **5671** | **6367** | **4180** | **4295** |
| **ssc-miR-1** | **48** | **256** | **31** | **44** | **38** | **29** | **132** | **196** | **2225** | **126** |
| **ssc-miR-100** | **703** | **68** | **38** | **23** | **683** | **401** | **1109** | **549** | **71** | **476** |
| **ssc-miR-101** | **1355** | **910** | **380** | **208** | **1307** | **3967** | **262** | **1127** | **532** | **334** |
| **ssc-miR-101** | **1355** | **910** | **380** | **208** | **1307** | **3967** | **262** | **1127** | **532** | **334** |
| **ssc-miR-103** | **728** | **730** | **212** | **186** | **108** | **164** | **186** | **543** | **890** | **210** |
| **ssc-miR-103** | **728** | **730** | **212** | **186** | **108** | **164** | **186** | **543** | **890** | **210** |
| **ssc-miR-105-2** |  |  |  |  |  |  |  |  |  | **1** |
| **ssc-miR-106a** | **814** | **14** | **10** | **8** | **62** | **23** | **43** | **64** | **49** | **276** |
| **ssc-miR-107** | **46** | **77** | **21** | **18** | **15** | **42** | **9** | **45** | **108** | **13** |
| **ssc-miR-10a-5p** | **5778** | **14652** | **13722** | **24730** | **9115** | **6951** | **15722** | **24106** | **12615** | **8010** |
| **ssc-miR-10a-3p** | **17** | **16** | **9** | **38** |  | **20** | **8** | **58** | **22** | **8** |
| **ssc-miR-10b** | **159484** | **78293** | **518280** | **482767** | **24165** | **27055** | **13186** | **106052** | **165201** | **110485** |
| **ssc-miR-122** | **153** | **242** | **79** | **90** | **25749** | **182882** | **133** | **363** | **207** | **865** |
| **ssc-miR-1249** |  | **2** |  |  | **3** | **1** | **1** |  | **1** | **2** |
| **ssc-miR-1249** |  | **2** |  |  | **3** | **1** | **1** |  | **1** | **2** |
| **ssc-miR-125a** | **107** | **333** | **148** | **168** | **135** | **65** | **180** | **267** | **313** | **132** |
| **ssc-miR-125b** | **44** | **56** | **22** | **53** | **53** | **29** | **46** | **64** | **113** | **31** |
| **ssc-miR-125b** | **44** | **56** | **22** | **53** | **53** | **29** | **46** | **64** | **113** | **31** |
| **ssc-miR-126-5p** | **2636** | **4950** | **684** | **1536** | **2147** | **1861** | **4225** | **3706** | **5018** | **1614** |
| **ssc-miR-126-3p** | **11214** | **6242** | **1727** | **256** | **1225** | **5953** | **2288** | **22568** | **1185** | **677** |
| **ssc-miR-127** | **22** | **23** | **1** | **1** | **8** | **4** | **7** | **4** | **3** | **26** |
| **ssc-miR-1277** |  |  |  |  |  |  |  |  | **1** |  |
| **ssc-miR-128** | **886** | **77** | **47** | **5** | **20** | **134** | **96** | **812** | **38** | **40** |
| **ssc-miR-128** | **886** | **77** | **47** | **5** | **20** | **134** | **96** | **812** | **38** | **40** |
| **ssc-miR-129a** |  | **1** |  |  |  | **1** |  |  |  |  |
| **ssc-miR-129b** | **55** | **48** | **4** | **10** | **11** | **4** | **19** | **13** | **50** | **39** |
| **ssc-miR-1306-5p** | **1** | **2** |  |  |  |  | **1** | **1** | **1** |  |
| **ssc-miR-1306-3p** | **19** | **4** | **1** | **1** | **6** | **4** | **12** | **9** | **19** | **9** |
| **ssc-miR-1307** | **30** | **15** | **5** | **4** | **6** | **6** | **7** | **19** | **17** | **14** |
| **ssc-miR-130a** | **84** | **202** | **67** | **52** | **163** | **78** | **258** | **212** | **195** | **178** |
| **ssc-miR-130b** | **43** | **7** | **1** | **2** | **14** | **6** | **13** | **14** | **29** | **36** |
| **ssc-miR-132** | **9** | **2** | **2** | **1** |  |  | **2** | **1** | **2** | **7** |
| **ssc-miR-133a-3p** |  | **4** |  | **1** | **1** |  | **6** | **5** | **7** | **5** |
| **ssc-miR-133a-3p** |  | **4** |  | **1** | **1** |  | **6** | **5** | **7** | **5** |
| **ssc-miR-133b** | **2** | **4** |  |  | **2** | **1** | **7** | **8** | **4** | **4** |
| **ssc-miR-1343** | **1** | **2** | **2** | **1** |  |  | **1** |  | **1** | **3** |
| **ssc-miR-136** | **12** | **5** |  |  | **2** | **1** | **4** | **3** |  | **6** |
| **ssc-miR-138** | **71** | **5** | **4** |  | **2** | **6** | **5** | **68** | **1** | **4** |
| **ssc-miR-139-5p** | **64** | **4** | **6** | **4** | **29** | **168** | **3** | **22** | **7** | **13** |
| **ssc-miR-139-3p** | **1** | **1** |  |  | **8** |  |  | **2** |  | **3** |
| **ssc-miR-140-5p** | **8** | **124** | **62** | **3** |  | **5** |  | **12** | **37** | **4** |
| **ssc-miR-140-3p** | **711** | **517** | **184** | **141** | **513** | **289** | **518** | **440** | **677** | **852** |
| **ssc-miR-142-5p** | **2281** | **472** | **50** | **62** | **286** | **128** | **229** | **190** | **2857** | **1304** |
| **ssc-miR-142-3p** | **152** | **205** | **20** | **2** | **7** | **5** | **5** | **15** | **66** | **22** |
| **ssc-miR-143-5p** |  | **2** |  |  | **2** |  | **3** |  | **1** | **2** |
| **ssc-miR-143-3p** | **20224** | **135219** | **14104** | **3843** | **29398** | **12500** | **76752** | **48819** | **56605** | **74621** |
| **ssc-miR-144** | **21** | **73** | **17** | **22** | **98** | **50** | **72** | **75** | **223** | **257** |
| **ssc-miR-145-5p** | **196** | **188** | **55** | **27** | **336** | **87** | **541** | **247** | **453** | **615** |
| **ssc-miR-145-3p** | **6** | **134** | **19** | **3** | **9** | **8** | **8** | **18** | **47** | **21** |
| **ssc-miR-1468** | **27** | **319** | **36** | **16** | **11** | **9** | **147** | **174** | **92** | **54** |
| **ssc-miR-146a-5p** | **718** | **232** | **25** | **34** | **279** | **44** | **369** | **114** | **808** | **1222** |
| **ssc-miR-146b** | **3837** | **3030** | **237** | **161** | **880** | **281** | **1754** | **1651** | **12746** | **4802** |
| **ssc-miR-148a-5p** | **160** | **173** | **31** | **27** | **237** | **274** | **89** | **162** | **135** | **56** |
| **ssc-miR-148a-3p** | **1637** | **4046** | **641** | **508** | **10806** | **6725** | **3415** | **2202** | **2471** | **2380** |
| **ssc-miR-148b-5p** | **10** | **2** | **1** |  | **2** | **4** | **1** | **4** | **9** | **6** |
| **ssc-miR-148b-3p** | **297** | **872** | **206** | **77** | **270** | **269** | **402** | **425** | **406** | **251** |
| **ssc-miR-149** |  |  |  |  |  |  |  |  | **1** |  |
| **ssc-miR-150** | **102** | **18** | **2** | **6** | **14** | **2** | **25** | **11** | **201** | **58** |
| **ssc-miR-150** | **102** | **18** | **2** | **6** | **14** | **2** | **25** | **11** | **201** | **58** |
| **ssc-miR-151-5p** | **236** | **334** | **112** | **160** | **425** | **185** | **265** | **233** | **471** | **276** |
| **ssc-miR-151-3p** | **446** | **596** | **157** | **252** | **289** | **284** | **223** | **491** | **583** | **216** |
| **ssc-miR-152** | **115** | **330** | **138** | **74** | **305** | **217** | **236** | **145** | **155** | **172** |
| **ssc-miR-153** | **1** |  |  |  |  | **1** |  | **1** | **1** | **1** |
| **ssc-miR-155-5p** | **6344** | **51** | **33** | **7** | **123** | **441** | **47** | **347** | **63** | **122** |
| **ssc-miR-155-3p** |  |  |  |  |  |  | **2** |  |  |  |
| **ssc-miR-15a** | **108** | **3358** | **389** | **51** | **195** | **66** | **264** | **88** | **426** | **351** |
| **ssc-miR-15b** | **187** | **147** | **32** | **12** | **200** | **28** | **318** | **53** | **99** | **481** |
| **ssc-miR-16** | **13522** | **2159** | **530** | **1178** | **7253** | **3209** | **8341** | **4879** | **14282** | **12623** |
| **ssc-miR-16** | **13522** | **2159** | **530** | **1178** | **7253** | **3209** | **8341** | **4879** | **14282** | **12623** |
| **ssc-miR-17-5p** | **3086** | **263** | **133** | **54** | **790** | **1173** | **627** | **1363** | **301** | **905** |
| **ssc-miR-17-3p** | **12** | **1** | **1** |  | **33** | **10** | **16** | **6** | **2** | **38** |
| **ssc-miR-181a** | **9249** | **11730** | **3390** | **7673** | **8675** | **2402** | **15412** | **7119** | **31020** | **20543** |
| **ssc-miR-181a** | **9249** | **11730** | **3390** | **7673** | **8675** | **2402** | **15412** | **7119** | **31020** | **20543** |
| **ssc-miR-181b** | **932** | **1096** | **256** | **318** | **601** | **207** | **1051** | **780** | **1486** | **1187** |
| **ssc-miR-181b** | **932** | **1096** | **256** | **318** | **601** | **207** | **1051** | **780** | **1486** | **1187** |
| **ssc-miR-181c** | **197** | **422** | **203** | **234** | **166** | **69** | **383** | **289** | **373** | **321** |
| **ssc-miR-181d-5p** | **100** | **278** | **131** | **112** | **92** | **34** | **193** | **178** | **228** | **169** |
| **ssc-miR-182** | **108** | **529** | **80** | **37** | **260** | **88** | **319** | **299** | **335** | **484** |
| **ssc-miR-183** | **3** | **17** | **1** | **1** | **3** | **2** | **8** | **15** | **2** |  |
| **ssc-miR-1839-5p** | **4** | **2** | **1** | **3** | **4** | **2** | **4** | **2** | **3** | **7** |
| **ssc-miR-1839-3p** |  |  |  |  |  |  |  |  |  | **1** |
| **ssc-miR-184** | **43** | **2** | **3** | **2** | **2** |  | **1** | **5** | **14** | **3** |
| **ssc-miR-185** | **6** | **9** | **4** | **4** | **16** | **4** | **20** | **4** | **36** | **26** |
| **ssc-miR-186** | **504** | **293** | **103** | **432** | **644** | **189** | **404** | **187** | **2238** | **653** |
| **ssc-miR-187** | **1** |  |  |  |  |  |  |  |  |  |
| **ssc-miR-18a** | **140** | **7** | **3** |  | **11** | **27** | **15** | **34** | **4** | **22** |
| **ssc-miR-18b** | **153** | **5** | **3** | **1** |  | **6** |  | **13** | **2** | **2** |
| **ssc-miR-190a** | **7** | **7** | **15** | **9** | **5** | **4** | **6** | **7** | **9** | **6** |
| **ssc-miR-190b** | **5** | **20** | **2** | **1** | **1** | **1** | **40** | **29** | **3** | **2** |
| **ssc-miR-191** | **5090** | **2780** | **940** | **1169** | **1778** | **1765** | **1230** | **2246** | **7015** | **2265** |
| **ssc-miR-192** | **593** | **2720** | **25129** | **34060** | **85031** | **108251** | **435** | **4686** | **4972** | **8070** |
| **ssc-miR-193a-5p** | **3** | **3** | **1** | **1** | **2** | **1** | **1** | **1** | **3** | **5** |
| **ssc-miR-193a-3p** |  | **5** | **1** |  | **2** | **2** | **2** | **2** | **2** | **2** |
| **ssc-miR-194a** | **2** | **5** | **47** | **119** | **660** | **101** | **4** | **8** | **12** | **29** |
| **ssc-miR-194b-5p** | **1** |  |  |  | **10** |  |  |  |  |  |
| **ssc-miR-195** | **135** | **256** | **22** | **20** | **2000** | **53** | **7627** | **149** | **86** | **1101** |
| **ssc-miR-196a** | **3** | **2** | **34** | **12** | **1** | **2** | **2** | **8** | **7** | **11** |
| **ssc-miR-196a** | **3** | **2** | **34** | **12** | **1** | **2** | **2** | **8** | **7** | **11** |
| **ssc-miR-196b-5p** | **1** | **6** | **93** | **39** | **2** | **2** | **1** | **10** | **14** | **8** |
| **ssc-miR-196b** | **1** | **6** | **93** | **39** | **2** | **2** | **1** | **10** | **14** | **8** |
| **ssc-miR-199a-5p** | **414** | **32** | **27** | **3** | **27** | **397** | **9** | **440** | **10** | **8** |
| **ssc-miR-199a-3p** | **625** | **877** | **248** | **488** | **695** | **476** | **493** | **599** | **1911** | **432** |
| **ssc-miR-199a-5p** | **414** | **32** | **27** | **3** | **27** | **397** | **9** | **440** | **10** | **8** |
| **ssc-miR-199a-3p** | **625** | **877** | **248** | **488** | **695** | **476** | **493** | **599** | **1911** | **432** |
| **ssc-miR-199b-5p** | **70** | **13** | **7** |  | **2** | **33** | **5** | **88** | **2** | **1** |
| **ssc-miR-199b-3p** | **625** | **877** | **248** | **488** | **695** | **476** | **493** | **599** | **1911** | **432** |
| **ssc-miR-19a** | **54** | **8** | **9** | **3** | **271** | **6** | **149** | **10** | **33** | **233** |
| **ssc-miR-19b** | **205** | **29** | **45** | **19** | **1471** | **55** | **587** | **44** | **142** | **807** |
| **ssc-miR-19b** | **205** | **29** | **45** | **19** | **1471** | **55** | **587** | **44** | **142** | **807** |
| **ssc-miR-202-5p** |  |  |  |  | **10** |  |  |  |  | **74** |
| **ssc-miR-204** | **51** | **411** | **1855** | **751** | **398** | **71** | **190** | **331** | **189** | **163** |
| **ssc-miR-205** | **2** | **17** | **6** | **1** |  |  | **14** | **25** | **4** | **2** |
| **ssc-miR-206** | **1** |  |  | **4** |  | **1** | **1** | **1** | **21** | **4** |
| **ssc-miR-208b** |  |  |  |  |  |  |  | **1** |  |  |
| **ssc-miR-20a** | **2534** | **298** | **150** | **75** | **1750** | **1570** | **1046** | **1225** | **372** | **1493** |
| **ssc-miR-20b** | **986** | **16** | **7** | **3** | **42** | **35** | **34** | **82** | **68** | **169** |
| **ssc-miR-20b** | **986** | **16** | **7** | **3** | **42** | **35** | **34** | **82** | **68** | **169** |
| **ssc-miR-21** | **56800** | **43984** | **6856** | **1614** | **21162** | **10140** | **24936** | **21935** | **14799** | **20733** |
| **ssc-miR-210** | **53** | **111** | **14** | **11** | **3** | **3** | **32** | **85** | **22** | **10** |
| **ssc-miR-212** | **1** | **4** | **2** |  |  |  |  |  | **2** |  |
| **ssc-miR-214** | **32** | **20** | **11** | **8** | **2786** | **52** | **1842** | **20** | **33** | **1715** |
| **ssc-miR-217** |  |  |  |  |  | **1** |  |  |  |  |
| **ssc-miR-217** |  |  |  |  |  | **1** |  |  |  |  |
| **ssc-miR-218-5p** | **297** | **196** | **80** | **17** | **83** | **124** | **595** | **1314** | **39** | **96** |
| **ssc-miR-218-3p** | **1** | **125** | **18** | **2** |  | **2** | **5** | **6** | **14** |  |
| **ssc-miR-218** | **297** | **196** | **80** | **17** | **83** | **124** | **595** | **1314** | **39** | **96** |
| **ssc-miR-218b** | **297** | **196** | **80** | **17** | **83** | **124** | **595** | **1314** | **39** | **96** |
| **ssc-miR-219** | **2** | **2** | **1** |  | **6** | **1** | **2** | **1** | **1** | **2** |
| **ssc-miR-22-5p** | **5** | **6** | **1** |  | **9** | **9** | **3** | **5** |  | **2** |
| **ssc-miR-22-3p** | **512** | **581** | **266** | **487** | **3388** | **1420** | **1519** | **546** | **632** | **1272** |
| **ssc-miR-221-5p** |  | **1** |  |  |  |  | **2** | **1** | **1** |  |
| **ssc-miR-221-3p** | **235** | **296** | **74** | **153** | **408** | **224** | **107** | **115** | **578** | **124** |
| **ssc-miR-222** | **18** | **10** | **3** | **9** | **75** | **14** | **12** | **6** | **52** | **17** |
| **ssc-miR-224** |  |  |  |  |  | **1** |  |  |  |  |
| **ssc-miR-2320-3p** | **3** | **1** |  |  |  | **1** |  | **1** | **2** |  |
| **ssc-miR-2366** |  |  |  |  |  |  | **1** |  |  | **1** |
| **ssc-miR-2366** |  |  |  |  |  |  | **1** |  |  | **1** |
| **ssc-miR-23a** | **99** | **203** | **39** | **16** | **201** | **28** | **393** | **103** | **137** | **431** |
| **ssc-miR-23b** | **47** | **305** | **111** | **40** | **190** | **39** | **511** | **170** | **94** | **243** |
| **ssc-miR-24-1-5p** | **4** | **4** |  |  |  | **1** | **7** | **3** | **5** | **4** |
| **ssc-miR-24-3p** | **327** | **909** | **251** | **177** | **11321** | **335** | **23461** | **355** | **671** | **12094** |
| **ssc-miR-24-2-5p** | **2** | **3** |  | **1** | **6** |  | **4** | **1** | **2** | **5** |
| **ssc-miR-24-3p** | **327** | **909** | **251** | **177** | **11321** | **335** | **23461** | **355** | **671** | **12094** |
| **ssc-miR-2411** | **3** |  |  |  | **2** | **2** |  | **1** | **1** | **4** |
| **ssc-miR-2483** | **1** | **4** |  |  |  |  | **1** | **2** | **1** |  |
| **ssc-miR-26a** | **39983** | **38545** | **11328** | **3456** | **11065** | **38895** | **9285** | **68431** | **11055** | **7728** |
| **ssc-miR-27a** | **1668** | **1448** | **217** | **30** | **115** | **306** | **219** | **1389** | **241** | **165** |
| **ssc-miR-27b-5p** | **4** | **12** | **2** | **1** | **4** | **2** | **38** | **7** | **5** | **6** |
| **ssc-miR-27b-3p** | **2491** | **12089** | **2932** | **1916** | **1127** | **5155** | **1819** | **11381** | **4563** | **1070** |
| **ssc-miR-28-5p** | **35** | **89** | **19** | **16** | **234** | **22** | **431** | **48** | **80** | **233** |
| **ssc-miR-28-3p** | **986** | **1119** | **352** | **265** | **917** | **585** | **1282** | **1154** | **1302** | **1501** |
| **ssc-miR-296-3p** | **9** | **21** | **8** | **3** | **47** | **4** | **35** | **5** | **13** | **48** |
| **ssc-miR-29a** | **124** | **42** | **13** | **56** | **312** | **81** | **56** | **25** | **423** | **156** |
| **ssc-miR-29b** | **22** | **7** | **2** | **4** | **31** | **13** | **12** | **5** | **17** | **43** |
| **ssc-miR-29b** | **22** | **7** | **2** | **4** | **31** | **13** | **12** | **5** | **17** | **43** |
| **ssc-miR-29c** | **25** | **13** | **6** | **2** | **22** | **7** | **31** | **9** | **12** | **56** |
| **ssc-miR-301** |  |  |  |  | **10** |  | **18** |  | **1** | **12** |
| **ssc-miR-30a-5p** | **14956** | **59694** | **34910** | **32929** | **70405** | **31475** | **85717** | **46471** | **47699** | **43086** |
| **ssc-miR-30a-3p** | **96** | **624** | **391** | **146** | **347** | **146** | **959** | **475** | **175** | **356** |
| **ssc-miR-30b-5p** | **402** | **728** | **394** | **279** | **1208** | **250** | **702** | **384** | **456** | **787** |
| **ssc-miR-30b-3p** | **2** | **15** | **6** | **3** | **11** | **4** | **6** | **7** | **5** | **6** |
| **ssc-miR-30c-5p** | **471** | **889** | **670** | **443** | **1005** | **279** | **1047** | **563** | **786** | **868** |
| **ssc-miR-30c-1-3p** | **3** | **8** | **5** | **1** |  | **1** | **1** | **2** | **4** |  |
| **ssc-miR-30c-5p** | **471** | **889** | **670** | **443** | **1005** | **279** | **1047** | **563** | **786** | **868** |
| **ssc-miR-30c-3p** | **17** | **33** | **33** | **55** | **35** | **26** | **73** | **76** | **62** | **40** |
| **ssc-miR-30d** | **2523** | **3421** | **1618** | **2838** | **3421** | **1914** | **2592** | **2490** | **6036** | **2427** |
| **ssc-miR-30e-5p** | **1857** | **1990** | **939** | **2219** | **2328** | **1225** | **1583** | **1291** | **6821** | **2284** |
| **ssc-miR-30e-3p** | **335** | **472** | **288** | **92** | **586** | **183** | **604** | **293** | **250** | **709** |
| **ssc-miR-31** | **5** | **4** | **1** | **2** |  |  | **5** | **2** | **4** |  |
| **ssc-miR-32** |  | **2** | **1** | **1** | **1** | **1** | **1** | **1** | **1** |  |
| **ssc-miR-320** | **103** | **987** | **155** | **22** | **274** | **149** | **254** | **219** | **210** | **128** |
| **ssc-miR-323** | **4** |  |  | **4** |  | **1** |  | **1** | **5** |  |
| **ssc-miR-324** |  | **2** | **1** | **1** |  | **1** | **1** | **1** |  | **2** |
| **ssc-miR-326** |  | **2** |  |  |  |  | **2** | **2** | **2** | **2** |
| **ssc-miR-328** |  | **1** |  |  |  |  |  |  |  | **1** |
| **ssc-miR-331-5p** | **1** | **3** |  | **1** | **1** |  | **1** | **1** | **3** |  |
| **ssc-miR-331-3p** | **1** |  |  |  |  | **1** | **3** |  | **1** | **6** |
| **ssc-miR-335** | **7** | **48** | **22** | **42** | **35** | **6** | **218** | **38** | **20** | **18** |
| **ssc-miR-339-5p** | **39** | **98** | **21** | **6** | **29** | **16** | **19** | **37** | **44** | **20** |
| **ssc-miR-339-3p** |  | **1** |  |  |  |  |  |  | **1** | **1** |
| **ssc-miR-339** | **39** | **98** | **21** | **6** | **29** | **16** | **19** | **37** | **44** | **20** |
| **ssc-miR-340** | **408** | **257** | **88** | **81** | **246** | **117** | **338** | **214** | **400** | **455** |
| **ssc-miR-340** | **408** | **257** | **88** | **81** | **246** | **117** | **338** | **214** | **400** | **455** |
| **ssc-miR-342** | **245** | **30** | **7** | **7** | **49** | **11** | **32** | **22** | **146** | **172** |
| **ssc-miR-345-5p** | **15** | **6** | **1** | **1** | **2** | **3** | **2** | **2** | **4** | **4** |
| **ssc-miR-345-3p** |  | **8** | **1** |  | **1** |  | **1** | **2** | **1** |  |
| **ssc-miR-345-5p** | **15** | **6** | **1** | **1** | **2** | **3** | **2** | **2** | **4** | **4** |
| **ssc-miR-345-3p** |  | **8** | **1** |  | **1** |  | **1** | **2** | **1** |  |
| **ssc-miR-34a** | **444** | **492** | **82** | **19** | **211** | **202** | **768** | **1111** | **151** | **302** |
| **ssc-miR-34c** | **146** | **83** | **63** | **4** |  | **6** | **93** | **1512** | **21** | **9** |
| **ssc-miR-34c** | **146** | **83** | **63** | **4** |  | **6** | **93** | **1512** | **21** | **9** |
| **ssc-miR-361-5p** | **3** | **8** | **3** | **1** | **33** |  | **23** | **2** | **3** | **38** |
| **ssc-miR-361-3p** | **72** | **5** | **2** | **1** | **4** | **18** | **2** | **32** | **5** | **6** |
| **ssc-miR-3613** | **47** | **116** | **20** | **9** | **63** | **21** | **76** | **42** | **41** | **102** |
| **ssc-miR-362** | **4** | **4** | **2** | **1** |  | **1** | **5** | **3** | **3** | **3** |
| **ssc-miR-363** | **35** | **8** | **4** | **9** | **5** | **2** | **2** | **2** | **60** | **32** |
| **ssc-miR-363** | **35** | **8** | **4** | **9** | **5** | **2** | **2** | **2** | **60** | **32** |
| **ssc-miR-365-3p** | **1** | **4** | **3** | **3** | **7** | **2** | **3** | **1** | **1** | **2** |
| **ssc-miR-365-5p** | **1** |  |  |  |  |  |  |  | **1** | **2** |
| **ssc-miR-365-3p** | **1** | **4** | **3** | **3** | **7** | **2** | **3** | **1** | **1** | **2** |
| **ssc-miR-369** | **54** | **20** |  | **4** |  |  |  |  |  | **14** |
| **ssc-miR-370** | **1** |  |  |  |  |  |  |  |  |  |
| **ssc-miR-374a-5p** | **292** | **379** | **153** | **73** | **291** | **100** | **363** | **228** | **222** | **418** |
| **ssc-miR-374a-3p** | **705** | **787** | **215** | **156** | **962** | **580** | **625** | **588** | **608** | **824** |
| **ssc-miR-374b-5p** | **19** | **65** | **29** | **9** | **31** | **11** | **23** | **21** | **40** | **48** |
| **ssc-miR-374b-3p** | **15** | **11** | **4** | **1** | **29** | **2** | **20** | **5** | **8** | **33** |
| **ssc-miR-376a-5p** |  |  |  |  |  |  |  |  | **1** |  |
| **ssc-miR-376a-3p** |  | **1** |  |  |  |  |  |  |  |  |
| **ssc-miR-376c** | **1** |  |  |  |  |  |  |  |  |  |
| **ssc-miR-378** | **940** | **1729** | **545** | **380** | **466** | **286** | **779** | **810** | **2473** | **1141** |
| **ssc-miR-378** | **940** | **1729** | **545** | **380** | **466** | **286** | **779** | **810** | **2473** | **1141** |
| **ssc-miR-382** | **29** | **6** | **1** | **1** | **3** | **4** | **5** | **4** | **1** | **8** |
| **ssc-miR-411** | **6** | **2** | **1** | **7** | **2** | **2** | **1** | **1** | **40** | **3** |
| **ssc-miR-421-3p** | **4** | **3** | **2** | **2** | **30** |  | **47** | **1** | **8** | **63** |
| **ssc-miR-423-5p** | **169** | **229** | **79** | **30** | **288** | **88** | **231** | **95** | **120** | **259** |
| **ssc-miR-423-3p** | **166** | **137** | **51** | **23** | **152** | **51** | **91** | **120** | **87** | **131** |
| **ssc-miR-424-5p** | **8** | **35** | **180** | **87** | **69** | **16** | **28** | **37** | **18** | **17** |
| **ssc-miR-424-3p** | **35** | **5** | **13** | **27** | **4** | **30** | **3** | **51** | **7** | **7** |
| **ssc-miR-425-5p** | **62** | **60** | **6** | **286** | **84** | **61** | **53** | **22** | **4221** | **151** |
| **ssc-miR-425-3p** | **73** | **104** | **19** | **2** | **29** | **28** | **14** | **36** | **22** | **16** |
| **ssc-miR-429** | **1** | **10** | **14** | **15** |  | **1** | **4** | **7** | **4** | **5** |
| **ssc-miR-432-5p** | **4** |  |  |  |  |  | **1** | **2** |  |  |
| **ssc-miR-4334-3p** | **39** | **98** | **21** | **6** | **29** | **16** | **19** | **37** | **44** | **20** |
| **ssc-miR-450a** | **4** | **11** | **17** | **517** | **16** | **17** | **6** | **9** | **85** | **84** |
| **ssc-miR-450b-5p** |  | **3** | **12** | **10** | **2** | **1** | **5** | **5** | **3** | **4** |
| **ssc-miR-450b-3p** |  |  |  | **2** | **1** | **1** |  |  |  | **1** |
| **ssc-miR-450c-5p** | **55** | **52** | **148** | **1417** | **42** | **87** | **17** | **86** | **228** | **242** |
| **ssc-miR-450c-3p** | **4** | **3** | **7** | **9** | **12** | **2** | **14** | **2** | **3** | **4** |
| **ssc-miR-451** | **63** | **360** | **67** | **60** | **454** | **185** | **221** | **301** | **596** | **944** |
| **ssc-miR-452** | **1** | **3** |  |  | **2** | **1** | **3** | **2** | **2** | **3** |
| **ssc-miR-455-5p** | **35** | **39** | **8** | **2** | **4** | **10** | **26** | **54** | **13** | **10** |
| **ssc-miR-455-3p** | **17** | **12** | **3** | **1** |  | **3** | **15** | **65** | **1** | **2** |
| **ssc-miR-486** | **44** | **138** | **31** | **96** | **152** | **291** | **118** | **298** | **450** | **218** |
| **ssc-miR-486** | **44** | **138** | **31** | **96** | **152** | **291** | **118** | **298** | **450** | **218** |
| **ssc-miR-490-5p** |  | **42** | **4** |  |  |  |  |  | **3** |  |
| **ssc-miR-490-3p** |  | **3** | **2** | **1** |  |  | **2** | **2** |  |  |
| **ssc-miR-490** |  | **3** | **2** | **1** |  |  | **2** | **2** |  |  |
| **ssc-miR-493-5p** | **2** | **1** |  |  |  | **1** | **1** | **1** |  |  |
| **ssc-miR-493-3p** |  | **1** |  |  |  |  |  |  |  |  |
| **ssc-miR-497** | **274** | **341** | **39** | **5** | **20** | **81** | **74** | **413** | **38** | **12** |
| **ssc-miR-499-5p** |  |  |  |  | **4** | **2** | **4** | **2** | **1** | **5** |
| **ssc-miR-500** | **22** | **21** | **14** | **4** | **1** | **1** | **11** | **12** | **6** | **7** |
| **ssc-miR-503** | **2** | **2** | **4** | **2** | **11** | **2** | **9** | **2** | **1** |  |
| **ssc-miR-504** |  |  |  |  | **2** |  | **1** |  |  |  |
| **ssc-miR-505** | **3** | **15** | **9** | **1** | **12** | **2** | **5** | **5** | **11** | **9** |
| **ssc-miR-532-5p** | **322** | **515** | **189** | **73** | **92** | **51** | **209** | **256** | **234** | **242** |
| **ssc-miR-532-3p** | **2** | **1** | **1** |  | **2** |  |  | **1** | **3** | **1** |
| **ssc-miR-542-5p** |  | **7** | **12** | **9** |  | **1** |  | **2** | **1** | **2** |
| **ssc-miR-542-3p** | **35** | **62** | **199** | **309** | **300** | **61** | **294** | **88** | **64** | **117** |
| **ssc-miR-545-5p** | **14** | **17** | **10** | **2** | **1** | **3** | **3** | **6** | **10** | **4** |
| **ssc-miR-545-3p** | **2** | **2** | **2** | **1** |  |  | **1** | **2** |  | **2** |
| **ssc-miR-551a** |  | **2** |  |  |  |  | **1** |  |  |  |
| **ssc-miR-574** | **4** | **4** | **1** | **1** | **5** | **1** | **12** | **4** | **7** | **4** |
| **ssc-miR-582** | **1** | **13** | **1** | **1** | **4** | **2** | **6** | **6** | **23** | **14** |
| **ssc-miR-628** | **1** | **1** | **2** | **2** | **3** | **1** | **5** |  | **6** | **6** |
| **ssc-miR-652** |  | **4** |  |  |  |  |  |  |  | **2** |
| **ssc-miR-664-5p** | **19** | **27** | **10** | **1** | **10** | **16** | **8** | **23** | **9** | **13** |
| **ssc-miR-664-3p** | **5** | **10** | **3** | **2** | **5** | **4** | **5** | **3** | **5** | **5** |
| **ssc-miR-671-5p** | **1** |  |  |  |  |  |  |  |  |  |
| **ssc-miR-671-3p** | **3** |  | **1** | **1** |  |  |  | **1** | **1** | **3** |
| **ssc-miR-676-5p** |  |  |  |  |  |  |  | **1** |  |  |
| **ssc-miR-676-3p** | **37** | **222** | **30** | **14** | **27** | **27** | **91** | **104** | **54** | **29** |
| **ssc-miR-676-3p** | **37** | **222** | **30** | **14** | **27** | **27** | **91** | **104** | **54** | **29** |
| **ssc-miR-7** | **2303** | **466** | **135** | **74** | **307** | **484** | **242** | **663** | **561** | **326** |
| **ssc-miR-7** | **2303** | **466** | **135** | **74** | **307** | **484** | **242** | **663** | **561** | **326** |
| **ssc-miR-708-5p** | **6** | **1** |  | **2** |  |  | **1** | **1** | **3** | **2** |
| **ssc-miR-708-3p** | **9** | **2** | **2** | **1** |  |  | **1** |  | **1** | **1** |
| **ssc-miR-744** | **10** | **12** | **6** | **2** | **3** | **9** | **5** | **4** | **11** | **3** |
| **ssc-miR-758** | **2** |  |  |  |  |  |  | **1** |  |  |
| **ssc-miR-769-5p** | **106** | **88** | **21** | **13** | **87** | **62** | **90** | **72** | **117** | **110** |
| **ssc-miR-769-3p** | **4** | **7** | **1** | **1** | **3** | **1** | **5** | **4** | **3** | **8** |
| **ssc-miR-874** | **1** | **6** | **8** | **4** | **37** | **11** | **6** | **6** | **2** | **1** |
| **ssc-miR-885-5p** |  | **1** |  |  | **11** | **3** |  |  |  |  |
| **ssc-miR-885-3p** |  |  |  |  | **81** | **35** | **4** | **1** |  | **2** |
| **ssc-miR-9-1** | **319** | **180** | **196** | **72** | **38** | **27** | **52** | **112** | **271** | **215** |
| **ssc-miR-9-2** | **319** | **180** | **196** | **72** | **38** | **27** | **52** | **112** | **271** | **215** |
| **ssc-miR-9** | **319** | **180** | **196** | **72** | **38** | **27** | **52** | **112** | **271** | **215** |
| **ssc-miR-92a** | **4475** | **6560** | **1289** | **1055** | **1585** | **2314** | **2232** | **3079** | **3991** | **1979** |
| **ssc-miR-92a** | **4475** | **6560** | **1289** | **1055** | **1585** | **2314** | **2232** | **3079** | **3991** | **1979** |
| **ssc-miR-92b-5p** | **1** |  |  |  |  |  | **1** | **3** | **1** |  |
| **ssc-miR-92b-3p** | **187** | **190** | **17** | **13** | **153** | **20** | **621** | **167** | **129** | **327** |
| **ssc-miR-95** | **24** | **152** | **9** | **3** | **29** | **23** | **49** | **63** | **27** | **12** |
| **ssc-miR-98** | **3378** | **2137** | **468** | **462** | **882** | **957** | **1012** | **1557** | **1529** | **1101** |
| **ssc-miR-99a** | **1796** | **97** | **135** | **114** | **2226** | **3389** | **1301** | **2380** | **180** | **1037** |
| **ssc-miR-99b** | **99** | **189** | **69** | **139** | **35** | **36** | **99** | **173** | **279** | **68** |
| **ssc-miR-novel-chr10_5472** |  |  |  |  |  |  |  |  |  | **1** |
| **ssc-miR-novel-chr10_5602** | **10** | **3** | **1** |  |  | **28** | **1** | **13** | **1** |  |
| **ssc-miR-novel-chr10_5682** |  | **2** |  |  |  |  |  |  |  |  |
| **ssc-miR-novel-chr10_6138** |  |  |  |  | **2** | **2** |  |  |  |  |
| **ssc-miR-novel-chr10_6327** | **1** |  |  |  |  |  |  |  |  |  |
| **ssc-miR-novel-chr11_6456** |  |  |  |  |  |  |  |  | **1** |  |
| **ssc-miR-novel-chr11_6577** |  | **10** | **10** |  |  | **1** | **1** | **1** | **3** | **2** |
| **ssc-miR-novel-chr11_6826** |  |  |  |  |  |  |  |  | **1** |  |
| **ssc-miR-novel-chr11_6942** |  |  |  |  | **1** |  |  |  | **1** | **2** |
| **ssc-miR-novel-chr11_7116** |  | **10** | **10** |  |  | **1** | **1** | **1** | **3** | **2** |
| **ssc-miR-novel-chr12_7407** | **1** |  |  |  | **2** | **1** |  |  |  |  |
| **ssc-miR-novel-chr12_7490** |  |  |  |  | **5** |  | **2** |  |  | **2** |
| **ssc-miR-novel-chr12_7511** |  |  |  |  |  |  |  |  | **1** |  |
| **ssc-miR-novel-chr12_7711** | **1** |  |  |  |  |  |  |  |  |  |
| **ssc-miR-novel-chr12_7775** |  |  |  |  | **2** |  |  |  |  |  |
| **ssc-miR-novel-chr12_7779** |  |  |  |  | **2** |  |  |  |  |  |
| **ssc-miR-novel-chr12_7811** | **1** |  |  |  |  |  |  |  |  |  |
| **ssc-miR-novel-chr12_7955** | **1** |  |  |  |  |  |  |  |  |  |
| **ssc-miR-novel-chr12_7961** | **358** | **116** | **42** | **23** | **149** | **67** | **281** | **129** | **137** | **325** |
| **ssc-miR-novel-chr12_7963** | **18** | **8** | **5** | **3** | **21** | **3** | **13** | **6** | **17** | **23** |
| **ssc-miR-novel-chr12_7964** | **18** | **8** | **5** | **3** | **21** | **3** | **13** | **6** | **17** | **23** |
| **ssc-miR-novel-chr12_8144** | **1** |  |  |  |  |  |  |  |  |  |
| **ssc-miR-novel-chr12_8161** |  | **2** |  |  |  | **1** | **1** | **1** | **2** | **2** |
| **ssc-miR-novel-chr12_8265** |  | **1** |  |  |  |  |  | **1** |  | **1** |
| **ssc-miR-novel-chr12_8282** |  | **4** | **1** |  |  |  |  |  |  |  |
| **ssc-miR-novel-chr12_8290** |  | **4** | **1** |  |  |  |  |  |  |  |
| **ssc-miR-novel-chr12_8420** |  |  |  |  | **4** |  | **10** |  |  | **6** |
| **ssc-miR-novel-chr12_8591** |  |  |  |  |  |  |  |  | **1** |  |
| **ssc-miR-novel-chr12_8618** |  | **1** |  |  |  |  |  |  |  |  |
| **ssc-miR-novel-chr12_8771** | **37** | **40** | **16** | **5** | **7** | **6** | **11** | **21** | **68** | **18** |
| **ssc-miR-novel-chr12_8979** |  |  |  |  |  |  |  |  |  | **2** |
| **ssc-miR-novel-chr12_8980** |  |  |  |  |  |  |  |  |  | **2** |
| **ssc-miR-novel-chr12_9188** |  | **1** |  |  |  |  |  |  |  |  |
| **ssc-miR-novel-chr13_10019** |  | **13** | **1** | **1** |  |  | **2** | **2** | **1** |  |
| **ssc-miR-novel-chr13_10170** | **27** | **10** | **1** | **1** | **2** |  | **5** | **2** | **3** | **8** |
| **ssc-miR-novel-chr13_10484** |  | **10** | **2** |  |  | **1** | **1** | **2** | **2** | **1** |
| **ssc-miR-novel-chr13_10658** |  |  |  |  | **51** | **2** |  |  |  | **2** |
| **ssc-miR-novel-chr13_10858** |  |  |  |  |  |  | **1** |  |  |  |
| **ssc-miR-novel-chr13_10861** | **38** | **352** | **159** | **71** | **95** | **52** | **118** | **127** | **125** | **96** |
| **ssc-miR-novel-chr13_10965** | **1** |  |  |  |  |  |  |  |  |  |
| **ssc-miR-novel-chr13_11226** | **2** |  |  |  |  |  |  |  |  |  |
| **ssc-miR-novel-chr13_11289** | **1** | **1** | **1** |  | **2** |  | **1** | **1** | **2** | **4** |
| **ssc-miR-novel-chr13_11481** |  | **1** |  |  |  |  | **1** |  | **1** | **1** |
| **ssc-miR-novel-chr13_11522** | **2** |  |  |  |  |  |  |  |  |  |
| **ssc-miR-novel-chr13_11601** |  | **1** |  |  |  | **1** |  | **1** | **2** | **1** |
| **ssc-miR-novel-chr13_11602** |  | **1** |  |  |  | **1** |  | **1** | **2** | **1** |
| **ssc-miR-novel-chr13_11871** |  |  |  |  |  |  | **1** |  |  |  |
| **ssc-miR-novel-chr13_11899** | **2** |  |  | **1** | **2** |  |  |  |  |  |
| **ssc-miR-novel-chr13_11944** | **1** |  |  |  |  |  |  |  |  |  |
| **ssc-miR-novel-chr13_12020** |  | **1** |  |  |  |  |  |  |  |  |
| **ssc-miR-novel-chr13_12101** | **1** |  |  |  |  | **1** |  |  | **2** | **1** |
| **ssc-miR-novel-chr13_9348** |  | **1** |  |  |  |  |  |  | **1** |  |
| **ssc-miR-novel-chr13_9430** |  |  |  | **1** |  |  | **1** |  | **1** |  |
| **ssc-miR-novel-chr13_9467** | **1** | **1** |  |  |  |  | **1** |  | **1** |  |
| **ssc-miR-novel-chr13_9841** | **1** |  |  |  |  |  |  |  |  |  |
| **ssc-miR-novel-chr13_9887** |  |  |  |  |  |  |  |  |  | **2** |
| **ssc-miR-novel-chr13_9996** | **1** |  |  |  |  |  |  |  |  |  |
| **ssc-miR-novel-chr14_12274** |  | **1** | **1** |  | **4** |  |  |  |  | **3** |
| **ssc-miR-novel-chr14_12484** |  |  |  |  |  |  |  |  | **1** |  |
| **ssc-miR-novel-chr14_12542** |  | **1** |  |  |  |  |  |  |  |  |
| **ssc-miR-novel-chr14_12801** | **1** |  |  |  | **2** |  |  |  |  |  |
| **ssc-miR-novel-chr14_12851** | **1** |  |  |  |  |  |  |  |  |  |
| **ssc-miR-novel-chr14_12918** | **1** |  |  |  |  |  | **1** |  |  |  |
| **ssc-miR-novel-chr14_13301** |  |  |  |  | **43** | **98** |  |  | **1** |  |
| **ssc-miR-novel-chr14_13321** |  |  |  |  |  |  |  |  | **2** | **1** |
| **ssc-miR-novel-chr14_13435** |  | **1** |  |  |  |  |  |  |  |  |
| **ssc-miR-novel-chr14_13772** | **1** |  |  |  |  |  |  |  |  |  |
| **ssc-miR-novel-chr14_13888** | **105** | **19** | **6** | **4** | **21** | **11** | **33** | **20** | **57** | **55** |
| **ssc-miR-novel-chr14_14614** | **1** | **1** |  |  |  |  |  |  |  |  |
| **ssc-miR-novel-chr15_14859** |  |  |  |  |  |  |  |  | **1** |  |
| **ssc-miR-novel-chr15_15025** | **1** |  |  |  |  |  |  |  |  |  |
| **ssc-miR-novel-chr15_15523** | **32** | **381** | **40** | **8** | **229** | **183** | **366** | **355** | **34** | **9** |
| **ssc-miR-novel-chr15_15531** |  |  |  |  |  |  | **1** |  |  |  |
| **ssc-miR-novel-chr15_15633** |  |  |  |  |  |  | **5** |  |  | **2** |
| **ssc-miR-novel-chr15_15673** | **9** | **26** | **4** |  | **1** | **2** | **2** | **4** | **2** |  |
| **ssc-miR-novel-chr15_16730** |  |  |  |  |  |  | **5** |  |  | **2** |
| **ssc-miR-novel-chr16_17181** |  | **1** |  |  |  |  |  | **1** |  |  |
| **ssc-miR-novel-chr16_17182** |  | **1** |  |  |  |  |  | **1** |  |  |
| **ssc-miR-novel-chr16_17391** | **68** | **197** | **33** | **11** | **170** | **54** | **449** | **248** | **43** | **182** |
| **ssc-miR-novel-chr16_17392** | **193** | **288** | **65** | **18** | **287** | **110** | **659** | **398** | **125** | **302** |
| **ssc-miR-novel-chr16_17559** | **292** | **2814** | **234** | **30** | **15** | **37** | **1151** | **2895** | **294** | **42** |
| **ssc-miR-novel-chr16_17561** | **63** | **202** | **38** | **4** | **1** | **5** | **54** | **710** | **22** | **7** |
| **ssc-miR-novel-chr16_17722** |  |  |  |  |  |  | **1** |  |  |  |
| **ssc-miR-novel-chr16_17741** |  |  |  |  |  |  | **1** |  |  |  |
| **ssc-miR-novel-chr17_18148** |  | **10** | **2** |  |  | **1** | **1** | **2** | **2** | **1** |
| **ssc-miR-novel-chr17_18195** |  | **1** |  |  |  | **1** | **1** |  |  |  |
| **ssc-miR-novel-chr17_18987** | **9** | **21** | **8** | **3** | **47** | **4** | **35** | **5** | **13** | **48** |
| **ssc-miR-novel-chr18_19238** |  | **1** |  |  | **1** |  | **2** |  |  |  |
| **ssc-miR-novel-chr18_19266** |  | **3** | **1** |  |  |  | **2** | **2** |  |  |
| **ssc-miR-novel-chr18_19420** |  | **5** |  |  |  |  | **4** | **1** |  |  |
| **ssc-miR-novel-chr18_19647** | **4** | **2** | **1** | **2** | **3** | **3** | **6** | **1** | **1** | **4** |
| **ssc-miR-novel-chr18_19745** |  |  |  |  | **2** | **1** |  |  |  |  |
| **ssc-miR-novel-chr18_19783** |  | **3** | **1** |  |  |  | **2** | **2** |  |  |
| **ssc-miR-novel-chr18_20001** | **1** |  |  |  |  |  |  |  |  |  |
| **ssc-miR-novel-chr18_20013** | **1** | **2** |  |  |  |  | **1** | **3** | **1** |  |
| **ssc-miR-novel-chr1_1012** |  |  |  |  |  |  | **1** |  |  |  |
| **ssc-miR-novel-chr1_1152** | **1** |  |  |  |  |  |  |  |  |  |
| **ssc-miR-novel-chr1_1175** | **1** |  |  |  |  | **1** |  |  | **1** |  |
| **ssc-miR-novel-chr1_1345** | **1** |  |  |  |  |  |  |  | **1** |  |
| **ssc-miR-novel-chr1_1497** | **3** | **7** | **1** | **1** | **2** | **2** | **1** | **2** | **3** | **5** |
| **ssc-miR-novel-chr1_1613** | **2** |  |  |  |  |  | **1** |  |  |  |
| **ssc-miR-novel-chr1_1798** | **25** | **3** |  |  | **7** | **4** | **6** | **3** | **13** | **12** |
| **ssc-miR-novel-chr1_1972** | **1** | **3** | **15** | **2** | **4** |  | **4** | **3** | **1** |  |
| **ssc-miR-novel-chr1_2074** | **24** |  |  |  | **2** | **1** |  | **2** | **2** | **4** |
| **ssc-miR-novel-chr1_2132** | **1** |  |  |  |  |  |  |  |  |  |
| **ssc-miR-novel-chr1_2311** | **1** |  |  |  |  |  |  |  |  |  |
| **ssc-miR-novel-chr1_2748** |  |  |  |  |  |  |  |  |  | **2** |
| **ssc-miR-novel-chr1_3279** |  |  |  |  | **2** |  |  |  |  |  |
| **ssc-miR-novel-chr1_3280** |  |  |  |  | **2** |  |  |  |  |  |
| **ssc-miR-novel-chr1_3515** |  |  |  |  |  |  |  |  |  | **1** |
| **ssc-miR-novel-chr1_3810** | **2** |  |  |  |  |  | **1** |  |  |  |
| **ssc-miR-novel-chr1_3896** | **1** |  |  |  |  |  |  |  |  |  |
| **ssc-miR-novel-chr1_3924** | **1** |  |  |  |  |  |  |  |  |  |
| **ssc-miR-novel-chr1_4339** | **1** |  |  |  |  |  |  |  |  |  |
| **ssc-miR-novel-chr1_4762** |  |  |  |  |  |  | **1** |  |  |  |
| **ssc-miR-novel-chr1_5092** |  | **1** |  |  |  |  | **1** | **1** |  |  |
| **ssc-miR-novel-chr1_5102** | **1** |  |  |  |  | **1** |  |  |  |  |
| **ssc-miR-novel-chr2_20089** | **1** | **1** |  |  |  |  |  |  |  |  |
| **ssc-miR-novel-chr2_20240** | **1** | **1** |  |  |  |  |  |  |  |  |
| **ssc-miR-novel-chr2_20299** | **1** |  |  |  |  |  |  |  |  |  |
| **ssc-miR-novel-chr2_20315** | **1** |  |  |  |  |  |  |  |  |  |
| **ssc-miR-novel-chr2_20345** | **1** |  |  |  |  |  |  |  |  |  |
| **ssc-miR-novel-chr2_20662** | **1** |  |  |  | **2** |  |  |  |  |  |
| **ssc-miR-novel-chr2_20965** | **4** | **1** |  | **2** | **4** |  | **9** | **1** | **5** | **4** |
| **ssc-miR-novel-chr2_21472** | **9** | **1** | **1** |  | **1** | **3** |  | **2** |  |  |
| **ssc-miR-novel-chr2_21617** | **2** |  |  |  |  |  |  |  | **1** |  |
| **ssc-miR-novel-chr2_21624** | **20224** | **135219** | **14104** | **3843** | **29398** | **12500** | **76752** | **48819** | **56605** | **74621** |
| **ssc-miR-novel-chr2_21774** | **3** |  |  |  |  |  | **1** |  | **2** |  |
| **ssc-miR-novel-chr2_21820** | **621** | **250** | **71** | **60** | **256** | **166** | **174** | **273** | **532** | **442** |
| **ssc-miR-novel-chr2_21982** |  |  |  |  |  |  | **1** |  | **1** |  |
| **ssc-miR-novel-chr2_22156** | **3** |  |  |  | **1** |  |  |  |  |  |
| **ssc-miR-novel-chr2_22194** |  |  |  |  |  |  | **1** |  |  |  |
| **ssc-miR-novel-chr2_22420** |  |  |  |  |  |  |  | **1** |  |  |
| **ssc-miR-novel-chr2_22453** |  |  |  |  |  |  |  |  |  | **2** |
| **ssc-miR-novel-chr2_22525** | **1** |  |  |  |  |  |  |  |  |  |
| **ssc-miR-novel-chr2_22652** |  |  |  |  |  | **1** |  |  |  |  |
| **ssc-miR-novel-chr2_22879** | **9** | **1** | **1** |  | **1** | **3** |  | **2** |  |  |
| **ssc-miR-novel-chr2_23068** | **3** |  |  |  |  |  |  |  |  |  |
| **ssc-miR-novel-chr3_23111** |  |  |  |  |  |  | **1** |  |  | **5** |
| **ssc-miR-novel-chr3_23263** |  |  |  |  |  |  |  |  | **1** |  |
| **ssc-miR-novel-chr3_23271** | **76** | **102** | **30** | **23** | **64** | **19** | **28** | **26** | **88** | **59** |
| **ssc-miR-novel-chr3_23361** |  |  |  |  | **1** |  | **1** | **1** |  |  |
| **ssc-miR-novel-chr3_23411** |  |  | **1** |  |  |  |  |  |  |  |
| **ssc-miR-novel-chr3_23465** |  |  |  |  |  |  |  | **1** |  |  |
| **ssc-miR-novel-chr3_24093** |  |  |  |  |  |  |  | **1** |  |  |
| **ssc-miR-novel-chr3_24244** |  | **2** | **8** | **6** | **4** |  |  | **3** | **1** | **3** |
| **ssc-miR-novel-chr3_24308** | **2** | **2** |  |  |  |  | **1** |  | **1** |  |
| **ssc-miR-novel-chr3_24388** |  |  |  |  |  |  | **1** |  |  | **5** |
| **ssc-miR-novel-chr3_24518** |  |  |  |  |  |  | **1** |  | **1** |  |
| **ssc-miR-novel-chr3_24548** |  |  | **1** |  |  | **1** |  | **1** |  |  |
| **ssc-miR-novel-chr3_24599** | **1** |  |  |  |  |  |  |  |  |  |
| **ssc-miR-novel-chr3_24739** | **2** |  |  |  |  |  |  |  |  |  |
| **ssc-miR-novel-chr3_24811** | **9** | **7** | **1** | **1** | **3** | **2** | **7** | **3** | **9** | **11** |
| **ssc-miR-novel-chr3_24812** | **9** | **7** | **1** | **1** | **3** | **2** | **7** | **3** | **9** | **11** |
| **ssc-miR-novel-chr3_24974** |  |  |  |  |  |  |  | **1** |  |  |
| **ssc-miR-novel-chr3_25115** |  |  |  |  |  |  |  | **1** |  |  |
| **ssc-miR-novel-chr3_25222** |  |  |  |  |  |  |  |  | **1** |  |
| **ssc-miR-novel-chr3_25240** | **2** | **5** | **3** | **1** | **2** | **2** | **5** | **8** | **3** |  |
| **ssc-miR-novel-chr3_25267** |  |  |  |  | **1** |  |  |  |  |  |
| **ssc-miR-novel-chr3_25350** | **1** | **6** |  | **1** | **2** | **1** |  | **2** | **2** |  |
| **ssc-miR-novel-chr3_25488** | **6** |  |  |  |  |  |  |  | **1** |  |
| **ssc-miR-novel-chr4_25600** |  |  |  |  |  |  | **2** |  |  |  |
| **ssc-miR-novel-chr4_25615** | **1** |  |  |  |  |  |  |  |  |  |
| **ssc-miR-novel-chr4_25914** |  |  |  |  |  |  |  |  |  | **2** |
| **ssc-miR-novel-chr4_25916** |  | **1** |  |  |  |  |  |  | **2** | **3** |
| **ssc-miR-novel-chr4_26000** | **1** | **1** |  |  |  |  |  |  |  | **3** |
| **ssc-miR-novel-chr4_26125** |  |  |  |  | **2** |  |  |  |  |  |
| **ssc-miR-novel-chr4_26129** |  |  |  |  |  |  |  |  |  | **1** |
| **ssc-miR-novel-chr4_26369** |  |  |  |  |  |  |  |  | **1** | **4** |
| **ssc-miR-novel-chr4_26691** | **1** |  |  |  |  |  |  |  |  |  |
| **ssc-miR-novel-chr4_26743** | **3** | **2** | **1** |  | **3** |  | **5** | **2** | **5** | **4** |
| **ssc-miR-novel-chr4_26821** |  |  | **2** |  | **2** |  |  | **1** |  | **1** |
| **ssc-miR-novel-chr4_26898** |  | **2** |  |  |  |  | **1** | **1** | **1** | **1** |
| **ssc-miR-novel-chr4_26969** |  | **1** |  |  |  |  |  |  |  |  |
| **ssc-miR-novel-chr4_27402** |  |  |  |  | **2** |  |  |  |  |  |
| **ssc-miR-novel-chr4_27574** |  |  |  |  |  | **1** |  |  | **1** |  |
| **ssc-miR-novel-chr4_27704** |  |  |  |  |  |  |  |  | **1** |  |
| **ssc-miR-novel-chr4_27733** | **1** |  |  |  |  |  |  |  |  |  |
| **ssc-miR-novel-chr4_27870** |  |  |  |  |  |  |  | **1** |  |  |
| **ssc-miR-novel-chr4_27873** |  |  |  |  |  |  | **1** |  |  |  |
| **ssc-miR-novel-chr4_27885** |  |  |  |  |  |  | **1** |  |  |  |
| **ssc-miR-novel-chr4_27887** |  |  |  |  |  |  | **1** |  |  |  |
| **ssc-miR-novel-chr4_27893** | **5** | **1** |  | **1** | **5** |  | **2** |  | **6** | **9** |
| **ssc-miR-novel-chr4_27930** | **1** |  |  |  |  |  |  |  | **1** |  |
| **ssc-miR-novel-chr4_28122** |  | **3** |  |  |  |  | **2** |  |  |  |
| **ssc-miR-novel-chr5_28279** | **358** | **251** | **60** | **27** | **73** | **96** | **60** | **208** | **195** | **118** |
| **ssc-miR-novel-chr5_28736** |  | **2** |  |  |  |  |  |  |  |  |
| **ssc-miR-novel-chr5_28846** |  |  |  |  | **2** |  | **1** |  |  |  |
| **ssc-miR-novel-chr5_29128** | **9** | **4** |  |  |  | **1** | **5** | **2** | **6** | **8** |
| **ssc-miR-novel-chr5_29391** | **1** | **2** | **1** |  |  |  | **1** |  |  | **3** |
| **ssc-miR-novel-chr5_29480** |  |  |  |  |  | **1** |  |  |  |  |
| **ssc-miR-novel-chr5_29627** | **2** | **1** |  |  |  |  |  | **1** |  |  |
| **ssc-miR-novel-chr5_29674** | **4** | **79** | **89** | **156** | **12** | **10** | **35** | **45** | **27** | **30** |
| **ssc-miR-novel-chr5_29676** | **4** | **125** | **19** | **4** |  | **2** | **49** | **30** | **18** | **2** |
| **ssc-miR-novel-chr5_29774** |  |  |  |  |  |  |  |  | **1** |  |
| **ssc-miR-novel-chr5_29781** |  |  |  |  |  |  |  | **1** |  |  |
| **ssc-miR-novel-chr5_29857** |  |  |  |  |  |  | **1** |  |  | **1** |
| **ssc-miR-novel-chr5_29868** |  |  |  |  | **2** |  |  |  |  |  |
| **ssc-miR-novel-chr6_30243** |  | **1** |  |  | **1** | **1** |  |  | **3** | **4** |
| **ssc-miR-novel-chr6_30244** |  | **1** |  |  | **1** | **1** |  |  | **3** | **4** |
| **ssc-miR-novel-chr6_30281** | **3** |  |  |  |  |  |  |  |  | **2** |
| **ssc-miR-novel-chr6_30729** |  | **4** | **11** | **17** |  | **1** | **6** | **4** | **2** | **3** |
| **ssc-miR-novel-chr6_30857** | **428** | **136** | **26** | **4** | **18** | **30** | **30** | **107** | **45** | **38** |
| **ssc-miR-novel-chr6_30922** |  | **2** |  |  |  |  |  |  |  |  |
| **ssc-miR-novel-chr6_31079** | **2** | **2** | **1** |  | **3** | **1** | **6** | **1** | **3** | **6** |
| **ssc-miR-novel-chr6_31604** |  | **2** |  |  | **2** | **1** | **1** | **1** |  | **3** |
| **ssc-miR-novel-chr6_31692** | **6** | **7** | **1** |  | **2** | **2** | **3** | **2** | **9** | **6** |
| **ssc-miR-novel-chr6_31759** | **1** |  |  |  |  |  |  |  | **1** |  |
| **ssc-miR-novel-chr6_32385** | **1** | **3** | **15** | **2** | **4** |  | **4** | **3** | **1** |  |
| **ssc-miR-novel-chr6_32446** |  |  |  |  |  |  |  |  | **2** |  |
| **ssc-miR-novel-chr6_32552** |  | **1** |  |  |  |  |  |  | **1** |  |
| **ssc-miR-novel-chr6_32557** | **5** | **1** | **1** |  |  | **1** | **2** | **6** | **1** | **2** |
| **ssc-miR-novel-chr6_32953** | **1** |  |  |  |  |  |  |  |  |  |
| **ssc-miR-novel-chr7_33108** |  | **2** | **2** |  |  |  |  | **1** |  |  |
| **ssc-miR-novel-chr7_33433** |  | **1** |  |  | **2** | **2** |  | **1** |  |  |
| **ssc-miR-novel-chr7_33502** | **1** | **1** |  |  |  |  | **1** |  | **1** |  |
| **ssc-miR-novel-chr7_33539** |  |  |  |  |  |  |  |  | **1** |  |
| **ssc-miR-novel-chr7_33656** | **1** |  |  |  |  |  |  |  |  |  |
| **ssc-miR-novel-chr7_33696** | **68** | **25** | **1** | **1** | **6** | **3** | **9** | **8** | **42** | **23** |
| **ssc-miR-novel-chr7_33821** |  |  | **1** | **1** | **3** | **4** |  |  | **1** | **3** |
| **ssc-miR-novel-chr7_34314** | **1** |  |  |  |  |  |  |  |  |  |
| **ssc-miR-novel-chr7_34318** | **1** |  |  |  |  | **1** |  |  |  |  |
| **ssc-miR-novel-chr7_34332** |  | **2** |  |  | **7** |  | **5** | **1** | **4** | **10** |
| **ssc-miR-novel-chr7_34819** |  |  |  |  | **1** |  |  |  |  |  |
| **ssc-miR-novel-chr7_34976** | **1** | **3** |  |  |  |  |  |  | **1** |  |
| **ssc-miR-novel-chr7_35053** |  |  |  |  |  |  |  | **1** |  |  |
| **ssc-miR-novel-chr7_35084** |  |  | **1** | **1** | **3** | **4** |  |  | **1** | **3** |
| **ssc-miR-novel-chr7_35271** |  |  |  |  |  |  | **1** |  |  |  |
| **ssc-miR-novel-chr7_35375** | **25** | **4** | **2** | **1** | **1** | **2** | **5** | **11** | **8** | **5** |
| **ssc-miR-novel-chr7_35579** |  |  |  |  |  |  |  |  |  | **2** |
| **ssc-miR-novel-chr8_35968** |  | **1** |  |  | **4** |  | **3** | **1** | **1** | **3** |
| **ssc-miR-novel-chr8_36342** |  |  |  |  |  |  |  |  |  | **3** |
| **ssc-miR-novel-chr8_36469** |  |  |  |  |  |  | **1** |  |  |  |
| **ssc-miR-novel-chr8_36601** | **1** | **1** |  |  |  |  |  |  |  |  |
| **ssc-miR-novel-chr8_36650** | **1** |  |  |  |  |  |  |  |  |  |
| **ssc-miR-novel-chr8_37190** | **10** | **25** | **11** | **17** | **7** | **5** | **12** | **11** | **15** | **8** |
| **ssc-miR-novel-chr8_37196** |  | **1** |  |  |  |  | **1** |  | **1** |  |
| **ssc-miR-novel-chr9_37440** |  | **1** |  |  |  |  | **1** |  | **1** |  |
| **ssc-miR-novel-chr9_37672** |  | **2** |  |  |  |  |  | **1** |  |  |
| **ssc-miR-novel-chr9_37686** | **3** | **2** | **1** |  | **3** |  | **2** | **1** | **5** | **3** |
| **ssc-miR-novel-chr9_37990** | **7** |  |  |  |  | **1** |  |  | **2** | **3** |
| **ssc-miR-novel-chr9_38471** |  |  |  |  | **1** |  |  |  |  |  |
| **ssc-miR-novel-chr9_38736** |  |  |  |  |  |  |  | **1** |  |  |
| **ssc-miR-novel-chr9_38827** | **64** | **53** | **26** | **2** | **1** | **3** | **130** | **546** | **8** | **6** |
| **ssc-miR-novel-chr9_38871** | **1** |  |  |  |  |  |  |  | **1** |  |
| **ssc-miR-novel-chr9_38959** | **1** |  |  |  |  | **1** |  |  |  |  |
| **ssc-miR-novel-chr9_39012** | **1** |  |  |  |  |  |  |  |  |  |
| **ssc-miR-novel-chr9_39041** | **7** |  |  |  |  | **1** |  |  | **2** | **3** |
| **ssc-miR-novel-chr9_39325** |  |  |  |  |  |  |  |  | **1** |  |
| **ssc-miR-novel-chrX_39845** |  | **6** |  | **1** | **6** | **2** | **2** | **1** | **2** | **8** |
| **ssc-miR-novel-chrX_39950** | **1** | **4** | **3** | **1** | **2** | **1** | **1** |  | **4** | **3** |
| **ssc-miR-novel-chrX_39952** | **251** | **212** | **141** | **98** | **184** | **44** | **356** | **136** | **223** | **499** |
| **ssc-miR-novel-chrX_39953** |  | **2** |  |  |  |  |  |  |  |  |
| **ssc-miR-novel-chrX_40068** |  |  |  |  | **6** |  | **4** |  |  | **6** |
| **ssc-miR-novel-chrX_40069** |  |  |  |  | **6** |  | **4** |  |  | **6** |
| **ssc-miR-novel-chrX_40461** |  |  |  |  | **2** |  |  |  |  |  |
| **ssc-miR-novel-chrX_40477** |  |  |  |  |  |  |  | **1** |  |  |
| **ssc-miR-novel-chrX_40484** | **1** | **2** |  |  |  |  | **1** | **1** |  |  |
| **ssc-miR-novel-chrX_40522** | **20** | **23** | **18** | **7** | **10** | **5** | **14** | **12** | **24** | **29** |
| **ssc-miR-novel-chrX_40528** |  |  |  |  |  |  | **1** |  |  | **3** |
| **ssc-miR-novel-chrX_40705** | **235** | **296** | **74** | **153** | **408** | **224** | **107** | **115** | **578** | **124** |
| **ssc-miR-novel-chrX_40912** |  |  |  |  |  |  |  |  |  | **1** |
| **ssc-miR-novel-chrX_41000** | **2** |  |  |  |  |  |  |  |  |  |
| **ssc-miR-novel-chrX_41061** |  |  |  |  | **1** |  | **3** |  |  | **6** |
| **ssc-miR-novel-GL892871-2_41708** | **11654** | **7559** | **2140** | **1203** | **4143** | **10317** | **2457** | **10524** | **5459** | **2347** |
| **ssc-miR-novel-GL893173-1_42029** | **1** | **3** | **1** |  | **3** | **2** |  | **1** |  |  |
| **ssc-miR-novel-GL893233-1_42087** |  |  |  |  | **4** |  |  |  |  |  |
| **ssc-miR-novel-GL893334-2_42199** |  | **1** |  |  |  |  | **1** | **1** |  |  |
| **ssc-miR-novel-GL894094-2_42949** | **9** | **26** | **4** |  | **1** | **2** | **2** | **4** | **2** |  |
| **ssc-miR-novel-GL894224-1_43070** |  | **1** |  |  |  |  |  |  |  |  |
| **ssc-miR-novel-GL894231-1_43077** | **3** | **1** |  |  | **2** | **2** |  | **1** |  | **1** |
| **ssc-miR-novel-GL894231-1_43090** | **1** | **2** | **1** | **3** |  |  | **3** |  | **14** | **4** |
| **ssc-miR-novel-GL894231-1_43098** | **2** | **1** |  |  |  |  |  | **1** |  | **2** |
| **ssc-miR-novel-GL894231-1_43100** | **2** |  |  |  |  |  |  |  |  |  |
| **ssc-miR-novel-GL894430-2_43273** | **2** |  |  |  |  |  | **1** |  |  |  |
| **ssc-miR-novel-GL894430-2_43278** | **2** |  |  |  |  |  | **1** |  |  |  |
| **ssc-miR-novel-GL894520-2_43333** | **1** |  |  |  |  |  |  |  |  |  |
| **ssc-miR-novel-GL894542-2_43355** |  |  |  | **1** |  |  | **1** |  | **1** |  |
| **ssc-miR-novel-GL894875-2_43662** | **1** |  |  |  | **1** | **1** | **2** | **1** |  | **2** |
| **ssc-miR-novel-GL894932-2_43717** |  | **1** |  |  |  |  |  |  |  |  |
| **ssc-miR-novel-GL895030-2_43791** |  |  |  |  |  |  | **1** |  |  | **5** |
| **ssc-miR-novel-GL895143-1_43893** | **1** |  |  |  |  |  |  |  |  |  |
| **ssc-miR-novel-GL895351-2_44043** |  |  |  |  |  |  | **1** |  |  |  |
| **ssc-miR-novel-GL895485-2_44130** |  |  |  |  |  | **1** |  |  |  |  |
| **ssc-miR-novel-GL895563-1_44191** |  |  |  |  |  |  | **1** |  |  |  |
| **ssc-miR-novel-GL896241-2_44636** |  | **5** |  |  |  |  | **4** | **1** |  |  |
| **ssc-miR-novel-GL896292-1_44714** |  | **1** |  | **1** |  | **1** | **3** |  |  |  |
| **ssc-miR-novel-GL896302-1_44731** |  | **1** |  |  | **2** |  | **5** | **1** |  | **6** |
| **ssc-miR-novel-GL896425-1_44856** | **3** | **8** | **13** | **91** | **1582** | **187** | **24** | **7** | **121** | **174** |
| **ssc-miR-novel-GL896485-1_44921** |  |  |  |  |  |  |  |  | **1** |  |
| **ssc-miR-novel-JH118484-1_41578** |  |  |  |  |  |  | **1** |  |  |  |
| **ssc-miR-novel-JH118486-1_41595** | **3** |  |  |  |  |  | **1** |  | **2** |  |
| **ssc-miR-novel-JH118494-1_41628** |  |  |  |  |  | **1** |  |  |  |  |
| **ssc-miR-novel-JH118570-1_42024** |  |  |  | **1** |  |  |  |  |  |  |
| **ssc-miR-novel-JH118585-1_42104** |  | **2** | **2** |  |  |  |  | **1** |  |  |
| **ssc-miR-novel-JH118644-1_42413** |  |  |  |  |  |  |  | **1** |  |  |
| **ssc-miR-novel-JH118654-1_42487** | **292** | **2814** | **234** | **30** | **15** | **37** | **1151** | **2895** | **294** | **42** |
| **ssc-miR-novel-JH118656-1_42504** | **6** | **11** | **3** | **4** | **20** | **3** | **55** | **11** | **10** | **37** |
| **ssc-miR-novel-JH118676-1_42617** | **1** |  |  |  |  | **1** |  |  | **1** |  |
| **ssc-miR-novel-JH118774-1_43297** | **1** |  |  |  |  |  |  |  | **1** |  |
| **ssc-miR-novel-JH118806-1_43515** |  |  |  |  | **3** |  |  |  |  |  |
| **ssc-miR-novel-JH118806-1_43518** |  |  |  |  | **3** |  |  |  |  |  |
| **ssc-miR-novel-JH118928-1_44242** | **1** |  |  |  |  |  |  | **1** | **2** |  |
| **ssc-miR-novel-JH118951-1_44333** |  |  |  |  |  |  |  |  |  | **1** |
| **ssc-miR-novel-JH118993-1_44650** |  | **1** |  |  |  |  |  |  | **1** |  |
| **ssc-miR-novel-JH118993-1_44655** | **5** | **1** | **1** |  |  | **1** | **2** | **6** | **1** | **2** |

**Table S3. Differentially expressed miRNAs.**

**Up-regulated miRNAs in PCMV infected lung**

| **MATURE-ID** | **pre-miRNA arm (5p or 3p)** | **Lung-PCMV** | **Lung-Control** | **Lung-PCMV vs Lung-Control** |
| --- | --- | --- | --- | --- |
| ssc-miR-101 | 3p | 1127 | 262 | 4.1801471 |
| ssc-miR-101 | 3p | 1127 | 262 | 4.1801471 |
| ssc-miR-103 | 3p | 543 | 186 | 2.8214286 |
| ssc-miR-103 | 3p | 543 | 186 | 2.8214286 |
| ssc-miR-107 | 3p | 45 | 9 | 2.8947368 |
| ssc-miR-10a-3p | 3p | 58 | 8 | 3.7777778 |
| ssc-miR-10b | 5p | 106052 | 13186 | 8.0374356 |
| ssc-miR-122 | 5p | 363 | 133 | 2.6083916 |
| ssc-miR-126-3p | 3p | 22568 | 2288 | 9.8250653 |
| ssc-miR-128 | 3p | 812 | 96 | 7.754717 |
| ssc-miR-128 | 3p | 812 | 96 | 7.754717 |
| ssc-miR-138 | 5p | 68 | 5 | 5.2 |
| ssc-miR-139-5p | 5p | 22 | 3 | 2.4615385 |
| ssc-miR-140-5p | 5p | 12 |  | 2.2 |
| ssc-miR-151-3p | 3p | 491 | 223 | 2.1502146 |
| ssc-miR-155-5p | 5p | 347 | 47 | 6.2631579 |
| ssc-miR-17-5p | 5p | 1363 | 627 | 2.155416 |
| ssc-miR-18b | 5p | 13 |  | 2.3 |
| ssc-miR-192 | 5p | 4686 | 435 | 10.552809 |
| ssc-miR-199a-5p | 5p | 440 | 9 | 23.684211 |
| ssc-miR-199a-5p | 5p | 440 | 9 | 23.684211 |
| ssc-miR-199b-5p | 5p | 88 | 5 | 6.5333333 |
| ssc-miR-20b | 5p | 82 | 34 | 2.0909091 |
| ssc-miR-20b | 5p | 82 | 34 | 2.0909091 |
| ssc-miR-210 | 3p | 85 | 32 | 2.2619048 |
| ssc-miR-218-5p | 5p | 1314 | 595 | 2.1884298 |
| ssc-miR-218 | 5p | 1314 | 595 | 2.1884298 |
| ssc-miR-218b | 5p | 1314 | 595 | 2.1884298 |
| ssc-miR-26a | 5p | 68431 | 9285 | 7.363206 |
| ssc-miR-27a | 3p | 1389 | 219 | 6.1091703 |
| ssc-miR-27b-3p | 3p | 11381 | 1819 | 6.2279934 |
| ssc-miR-34c | 5p | 1512 | 93 | 14.776699 |
| ssc-miR-34c | 5p | 1512 | 93 | 14.776699 |
| ssc-miR-361-3p | 3p | 32 | 2 | 3.5 |
| ssc-miR-424-3p | 3p | 51 | 3 | 4.6923077 |
| ssc-miR-450c-5p | 5p | 86 | 17 | 3.5555556 |
| ssc-miR-455-3p | 3p | 65 | 15 | 3 |
| ssc-miR-486 | 3p | 298 | 118 | 2.40625 |
| ssc-miR-486 | 5p | 298 | 118 | 2.40625 |
| ssc-miR-497 | 5p | 413 | 74 | 5.0357143 |
| ssc-miR-7 | 5p | 663 | 242 | 2.6706349 |
| ssc-miR-7 | 5p | 663 | 242 | 2.6706349 |
| ssc-miR-novel-chr10_5602 | 5p | 13 | 1 | 2.0909091 |
| ssc-miR-novel-chr16_17559 | 5p | 2895 | 1151 | 2.5021533 |
| ssc-miR-novel-chr16_17561 | 5p | 710 | 54 | 11.25 |
| ssc-miR-novel-chr5_28279 | 3p | 208 | 60 | 3.1142857 |
| ssc-miR-novel-chr6_30857 | 3p | 107 | 30 | 2.925 |
| ssc-miR-novel-chr9_38827 | 5p | 546 | 130 | 3.9714286 |
| ssc-miR-novel-GL892871-2_41708 | 5p | 10524 | 2457 | 4.2699635 |
| ssc-miR-novel-JH118654-1_42487 | 5p | 2895 | 1151 | 2.5021533 |

**Down-regulated miRNAs in PCMV infected lung**

| **MATURE-ID** | **pre-miRNA arm (5p or 3p)** | **Lung-PCMV** | **Lung-Control** | **Lung-PCMVvs Lung-Control** |
| --- | --- | --- | --- | --- |
| ssc-let-7d-5p | 5p | 118 | 1084 | 0.1170018 |
| ssc-let-7f | 5p | 15185 | 32534 | 0.4669063 |
| ssc-let-7f | 5p | 15185 | 32534 | 0.4669063 |
| ssc-miR-100 | 5p | 549 | 1109 | 0.4995532 |
| ssc-miR-145-5p | 5p | 247 | 541 | 0.4664247 |
| ssc-miR-146a-5p | 5p | 114 | 369 | 0.3271768 |
| ssc-miR-15a | 5p | 88 | 264 | 0.3576642 |
| ssc-miR-15b | 5p | 53 | 318 | 0.1920732 |
| ssc-miR-181a | 5p | 7119 | 15412 | 0.4622617 |
| ssc-miR-181a | 5p | 7119 | 15412 | 0.4622617 |
| ssc-miR-185 | 5p | 4 | 20 | 0.4666667 |
| ssc-miR-186 | 5p | 187 | 404 | 0.4758454 |
| ssc-miR-195 | 5p | 149 | 7627 | 0.0208197 |
| ssc-miR-19a | 3p | 10 | 149 | 0.1257862 |
| ssc-miR-19b | 3p | 44 | 587 | 0.0904523 |
| ssc-miR-19b | 3p | 44 | 587 | 0.0904523 |
| ssc-miR-214 | 3p | 20 | 1842 | 0.0161987 |
| ssc-miR-22-3p | 3p | 546 | 1519 | 0.3636364 |
| ssc-miR-23a | 3p | 103 | 393 | 0.280397 |
| ssc-miR-23b | 3p | 170 | 511 | 0.3454894 |
| ssc-miR-24-3p | 3p | 355 | 23461 | 0.0155511 |
| ssc-miR-24-3p | 3p | 355 | 23461 | 0.0155511 |
| ssc-miR-27b-5p | 5p | 7 | 38 | 0.3541667 |
| ssc-miR-28-5p | 5p | 48 | 431 | 0.1315193 |
| ssc-miR-296-3p | 3p | 5 | 35 | 0.3333333 |
| ssc-miR-29c | 3p | 9 | 31 | 0.4634146 |
| ssc-miR-301 | 3p |  | 18 | 0.3571429 |
| ssc-miR-30e-3p | 3p | 293 | 604 | 0.4934853 |
| ssc-miR-335 | 5p | 38 | 218 | 0.2105263 |
| ssc-miR-361-5p | 5p | 2 | 23 | 0.3636364 |
| ssc-miR-374b-3p | 3p | 5 | 20 | 0.5 |
| ssc-miR-421-3p | 3p | 1 | 47 | 0.1929825 |
| ssc-miR-423-5p | 5p | 95 | 231 | 0.4356846 |
| ssc-miR-450c-3p | 3p | 2 | 14 | 0.5 |
| ssc-miR-542-3p | 3p | 88 | 294 | 0.3223684 |
| ssc-miR-92b-3p | 3p | 167 | 621 | 0.2805071 |
| ssc-miR-novel-chr12_7961 | 3p | 129 | 281 | 0.4776632 |
| ssc-miR-novel-chr12_8420 | 3p |  | 10 | 0.5 |
| ssc-miR-novel-chr17_18987 | 5p | 5 | 35 | 0.3333333 |
| ssc-miR-novel-chrX_39952 | 5p | 136 | 356 | 0.3989071 |
| ssc-miR-novel-GL896425-1_44856 | 3p | 7 | 24 | 0.5 |
| ssc-miR-novel-JH118656-1_42504 | 5p | 11 | 55 | 0.3230769 |

**Up-regulated miRNAs in PCMV infected thymus**

| **MATURE-ID** | **pre-miRNA arm (5p or 3p)** | **Thymus-PCMV** | **Thymus-Control** | **Thymus-PCMV vs Thymus-Control** |
| --- | --- | --- | --- | --- |
| ssc-let-7e | 5p | 769 | 315 | 2.3969231 |
| ssc-miR-1 | 3p | 256 | 48 | 4.5862069 |
| ssc-miR-10a-5p | 5p | 14652 | 5778 | 2.5331721 |
| ssc-miR-125a | 5p | 333 | 107 | 2.9316239 |
| ssc-miR-130a | 3p | 202 | 84 | 2.2553191 |
| ssc-miR-140-5p | 5p | 124 | 8 | 7.4444444 |
| ssc-miR-143-3p | 3p | 135219 | 20224 | 6.6832559 |
| ssc-miR-144 | 3p | 73 | 21 | 2.6774194 |
| ssc-miR-145-3p | 3p | 134 | 6 | 9 |
| ssc-miR-1468 | 5p | 319 | 27 | 8.8918919 |
| ssc-miR-148a-3p | 3p | 4046 | 1637 | 2.4626594 |
| ssc-miR-148b-3p | 3p | 872 | 297 | 2.8729642 |
| ssc-miR-152 | 3p | 330 | 115 | 2.72 |
| ssc-miR-15a | 5p | 3358 | 108 | 28.542373 |
| ssc-miR-181c | 5p | 422 | 197 | 2.0869565 |
| ssc-miR-181d-5p | 5p | 278 | 100 | 2.6181818 |
| ssc-miR-182 | 5p | 529 | 108 | 4.5677966 |
| ssc-miR-183 | 5p | 17 | 3 | 2.0769231 |
| ssc-miR-190b | 5p | 20 | 5 | 2 |
| ssc-miR-192 | 5p | 2720 | 593 | 4.5273632 |
| ssc-miR-204 | 5p | 411 | 51 | 6.9016393 |
| ssc-miR-205 | 5p | 17 | 2 | 2.25 |
| ssc-miR-218-3p | 3p | 125 | 1 | 12.272727 |
| ssc-miR-23b | 3p | 305 | 47 | 5.5263158 |
| ssc-miR-24-3p | 3p | 909 | 327 | 2.727003 |
| ssc-miR-24-3p | 3p | 909 | 327 | 2.727003 |
| ssc-miR-27b-3p | 3p | 12089 | 2491 | 4.8376649 |
| ssc-miR-28-5p | 5p | 89 | 35 | 2.2 |
| ssc-miR-30a-5p | 5p | 59694 | 14956 | 3.9893091 |
| ssc-miR-30a-3p | 3p | 624 | 96 | 5.9811321 |
| ssc-miR-30b-3p | 3p | 15 | 2 | 2.0833333 |
| ssc-miR-320 | 3p | 987 | 103 | 8.8230088 |
| ssc-miR-335 | 5p | 48 | 7 | 3.4117647 |
| ssc-miR-339-5p | 5p | 98 | 39 | 2.2040816 |
| ssc-miR-339 | 5p | 98 | 39 | 2.2040816 |
| ssc-miR-3613 | 5p | 116 | 47 | 2.2105263 |
| ssc-miR-374b-5p | 5p | 65 | 19 | 2.5862069 |
| ssc-miR-424-5p | 5p | 35 | 8 | 2.5 |
| ssc-miR-4334-3p | 3p | 98 | 39 | 2.2040816 |
| ssc-miR-451 | 5p | 360 | 63 | 5.0684932 |
| ssc-miR-486 | 3p | 138 | 44 | 2.7407407 |
| ssc-miR-486 | 5p | 138 | 44 | 2.7407407 |
| ssc-miR-490-5p | 5p | 42 |  | 5.2 |
| ssc-miR-582 | 3p | 13 | 1 | 2.0909091 |
| ssc-miR-676-3p | 3p | 222 | 37 | 4.9361702 |
| ssc-miR-676-3p | 3p | 222 | 37 | 4.9361702 |
| ssc-miR-95 | 3p | 152 | 24 | 4.7647059 |
| ssc-miR-novel-chr11_6577 | 3p | 10 |  | 2 |
| ssc-miR-novel-chr11_7116 | 3p | 10 |  | 2 |
| ssc-miR-novel-chr13_10019 | 3p | 13 |  | 2.3 |
| ssc-miR-novel-chr13_10484 | 5p | 10 |  | 2 |
| ssc-miR-novel-chr13_10861 | 5p | 352 | 38 | 7.5416667 |
| ssc-miR-novel-chr15_15523 | 3p | 381 | 32 | 9.3095238 |
| ssc-miR-novel-chr16_17391 | 5p | 197 | 68 | 2.6538462 |
| ssc-miR-novel-chr16_17559 | 5p | 2814 | 292 | 9.3509934 |
| ssc-miR-novel-chr16_17561 | 5p | 202 | 63 | 2.9041096 |
| ssc-miR-novel-chr17_18148 | 5p | 10 |  | 2 |
| ssc-miR-novel-chr2_21624 | 5p | 135219 | 20224 | 6.6832559 |
| ssc-miR-novel-chr5_29674 | 3p | 79 | 4 | 6.3571429 |
| ssc-miR-novel-chr5_29676 | 3p | 125 | 4 | 9.6428571 |
| ssc-miR-novel-JH118654-1_42487 | 5p | 2814 | 292 | 9.3509934 |

**Down-regulated miRNAs in PCMV infected thymus**

| **MATURE-ID** | **pre-miRNA arm (5p or 3p)** | **Thymus-PCMV** | **Thymus-Control** | **Thymus-PCMV vs Thymus-Control** |
| --- | --- | --- | --- | --- |
| ssc-let-7g | 5p | 3861 | 10112 | 0.3824343 |
| ssc-miR-100 | 5p | 68 | 703 | 0.1093969 |
| ssc-miR-106a | 5p | 14 | 814 | 0.0291262 |
| ssc-miR-10b | 5p | 78293 | 159484 | 0.4909464 |
| ssc-miR-128 | 3p | 77 | 886 | 0.0970982 |
| ssc-miR-128 | 3p | 77 | 886 | 0.0970982 |
| ssc-miR-1306-3p | 3p | 4 | 19 | 0.4827586 |
| ssc-miR-130b | 3p | 7 | 43 | 0.3207547 |
| ssc-miR-138 | 5p | 5 | 71 | 0.1851852 |
| ssc-miR-139-5p | 5p | 4 | 64 | 0.1891892 |
| ssc-miR-142-5p | 5p | 472 | 2281 | 0.2103885 |
| ssc-miR-146a-5p | 5p | 232 | 718 | 0.3324176 |
| ssc-miR-150 | 5p | 18 | 102 | 0.25 |
| ssc-miR-150 | 5p | 18 | 102 | 0.25 |
| ssc-miR-155-5p | 5p | 51 | 6344 | 0.0096003 |
| ssc-miR-16 | 5p | 2159 | 13522 | 0.1602867 |
| ssc-miR-16 | 5p | 2159 | 13522 | 0.1602867 |
| ssc-miR-17-5p | 5p | 263 | 3086 | 0.0881783 |
| ssc-miR-17-3p | 3p | 1 | 12 | 0.5 |
| ssc-miR-184 | 3p | 2 | 43 | 0.2264151 |
| ssc-miR-18a | 5p | 7 | 140 | 0.1133333 |
| ssc-miR-18b | 5p | 5 | 153 | 0.0920245 |
| ssc-miR-199a-5p | 5p | 32 | 414 | 0.0990566 |
| ssc-miR-199a-5p | 5p | 32 | 414 | 0.0990566 |
| ssc-miR-199b-5p | 5p | 13 | 70 | 0.2875 |
| ssc-miR-19a | 3p | 8 | 54 | 0.28125 |
| ssc-miR-19b | 3p | 29 | 205 | 0.1813953 |
| ssc-miR-19b | 3p | 29 | 205 | 0.1813953 |
| ssc-miR-20a | 5p | 298 | 2534 | 0.1210692 |
| ssc-miR-20b | 5p | 16 | 986 | 0.0261044 |
| ssc-miR-20b | 5p | 16 | 986 | 0.0261044 |
| ssc-miR-29a | 3p | 42 | 124 | 0.3880597 |
| ssc-miR-342 | 3p | 30 | 245 | 0.1568627 |
| ssc-miR-361-3p | 3p | 5 | 72 | 0.1829268 |
| ssc-miR-363 | 3p | 8 | 35 | 0.4 |
| ssc-miR-363 | 3p | 8 | 35 | 0.4 |
| ssc-miR-369 | 3p | 20 | 54 | 0.46875 |
| ssc-miR-382 | 5p | 6 | 29 | 0.4102564 |
| ssc-miR-424-3p | 3p | 5 | 35 | 0.3333333 |
| ssc-miR-7 | 5p | 466 | 2303 | 0.2057933 |
| ssc-miR-7 | 5p | 466 | 2303 | 0.2057933 |
| ssc-miR-99a | 5p | 97 | 1796 | 0.059247 |
| ssc-miR-novel-chr12_7961 | 3p | 116 | 358 | 0.3423913 |
| ssc-miR-novel-chr14_13888 | 3p | 19 | 105 | 0.2521739 |
| ssc-miR-novel-chr1_1798 | 5p | 3 | 25 | 0.3714286 |
| ssc-miR-novel-chr1_2074 | 5p |  | 24 | 0.2941176 |
| ssc-miR-novel-chr2_21820 | 3p | 250 | 621 | 0.4120444 |
| ssc-miR-novel-chr6_30857 | 3p | 136 | 428 | 0.3333333 |
| ssc-miR-novel-chr7_33696 | 5p | 25 | 68 | 0.4487179 |
| ssc-miR-novel-chr7_35375 | 5p | 4 | 25 | 0.4 |

**Up-regulated miRNAs in PCMV infected kidney**

| **MATURE-ID** | **pre-miRNA arm (5p or 3p)** | **Kidney-Virus** | **Kidney-Control** | **Kidney-Virus vs Kidney-Control** |
| --- | --- | --- | --- | --- |
| ssc-miR-10a-3p | 3p | 38 | 9 | 2.5263158 |
| ssc-miR-126-5p | 5p | 1536 | 684 | 2.2276657 |
| ssc-miR-16 | 5p | 1178 | 530 | 2.2 |
| ssc-miR-16 | 5p | 1178 | 530 | 2.2 |
| ssc-miR-181a | 5p | 7673 | 3390 | 2.2597059 |
| ssc-miR-181a | 5p | 7673 | 3390 | 2.2597059 |
| ssc-miR-186 | 5p | 432 | 103 | 3.9115044 |
| ssc-miR-194a | 5p | 119 | 47 | 2.2631579 |
| ssc-miR-29a | 3p | 56 | 13 | 2.8695652 |
| ssc-miR-30e-5p | 5p | 2219 | 939 | 2.3487882 |
| ssc-miR-425-5p | 5p | 286 | 6 | 18.5 |
| ssc-miR-450a | 5p | 517 | 17 | 19.518519 |
| ssc-miR-450c-5p | 5p | 1417 | 148 | 9.0316456 |
| ssc-miR-486 | 3p | 96 | 31 | 2.5853659 |
| ssc-miR-486 | 5p | 96 | 31 | 2.5853659 |
| ssc-miR-novel-GL896425-1_44856 | 3p | 91 | 13 | 4.3913043 |

**Down-regulated miRNAs in PCMV infected kidney**

| **MATURE-ID** | **pre-miRNA arm (5p or 3p)** | | **Kidney-Virus** | **Kidney-Control** | **Kidney-Virus vs Kidney-Control** |
| --- | --- | --- | --- | --- | --- |
| ssc-let-7a | 5p | 1495 | | 4242 | 0.3539511 |
| ssc-let-7a | 5p | 1495 | | 4242 | 0.3539511 |
| ssc-let-7c | 5p | 144 | | 489 | 0.3086172 |
| ssc-let-7e | 5p | 96 | | 346 | 0.2977528 |
| ssc-let-7f | 5p | 3457 | | 7432 | 0.4658694 |
| ssc-let-7f | 5p | 3457 | | 7432 | 0.4658694 |
| ssc-miR-126-3p | 3p | 256 | | 1727 | 0.1531376 |
| ssc-miR-128 | 3p | 5 | | 47 | 0.2631579 |
| ssc-miR-128 | 3p | 5 | | 47 | 0.2631579 |
| ssc-miR-140-5p | 5p | 3 | | 62 | 0.1805556 |
| ssc-miR-142-3p | 3p | 2 | | 20 | 0.4 |
| ssc-miR-143-3p | 3p | 3843 | | 14104 | 0.2729914 |
| ssc-miR-145-3p | 3p | 3 | | 19 | 0.4482759 |
| ssc-miR-148b-3p | 3p | 77 | | 206 | 0.4027778 |
| ssc-miR-155-5p | 5p | 7 | | 33 | 0.3953488 |
| ssc-miR-15a | 5p | 51 | | 389 | 0.1528822 |
| ssc-miR-17-5p | 5p | 54 | | 133 | 0.4475524 |
| ssc-miR-196a | 5p | 12 | | 34 | 0.5 |
| ssc-miR-196a | 5p | 12 | | 34 | 0.5 |
| ssc-miR-196b-5p | 5p | 39 | | 93 | 0.4757282 |
| ssc-miR-196b | 5p | 39 | | 93 | 0.4757282 |
| ssc-miR-199a-5p | 5p | 3 | | 27 | 0.3513514 |
| ssc-miR-199a-5p | 5p | 3 | | 27 | 0.3513514 |
| ssc-miR-204 | 5p | 751 | | 1855 | 0.4080429 |
| ssc-miR-21 | 5p | 1614 | | 6856 | 0.2365278 |
| ssc-miR-218-5p | 5p | 17 | | 80 | 0.3 |
| ssc-miR-218-3p | 3p | 2 | | 18 | 0.4285714 |
| ssc-miR-218 | 5p | 17 | | 80 | 0.3 |
| ssc-miR-218b | 5p | 17 | | 80 | 0.3 |
| ssc-miR-23b | 3p | 40 | | 111 | 0.4132231 |
| ssc-miR-26a | 5p | 3456 | | 11328 | 0.3056977 |
| ssc-miR-27a | 3p | 30 | | 217 | 0.1762115 |
| ssc-miR-30a-3p | 3p | 146 | | 391 | 0.3890274 |
| ssc-miR-30e-3p | 3p | 92 | | 288 | 0.3422819 |
| ssc-miR-320 | 3p | 22 | | 155 | 0.1939394 |
| ssc-miR-34a | 5p | 19 | | 82 | 0.3152174 |
| ssc-miR-34c | 5p | 4 | | 63 | 0.1917808 |
| ssc-miR-34c | 5p | 4 | | 63 | 0.1917808 |
| ssc-miR-374b-5p | 5p | 9 | | 29 | 0.4871795 |
| ssc-miR-423-5p | 5p | 30 | | 79 | 0.4494382 |
| ssc-miR-425-3p | 3p | 2 | | 19 | 0.4137931 |
| ssc-miR-497 | 5p | 5 | | 39 | 0.3061224 |
| ssc-miR-532-5p | 5p | 73 | | 189 | 0.4170854 |
| ssc-miR-9-1 | 5p | 72 | | 196 | 0.3980583 |
| ssc-miR-9-2 | 5p | 72 | | 196 | 0.3980583 |
| ssc-miR-9 | 5p | 72 | | 196 | 0.3980583 |
| ssc-miR-novel-chr11_6577 | 3p |  | | 10 | 0.5 |
| ssc-miR-novel-chr11_7116 | 3p |  | | 10 | 0.5 |
| ssc-miR-novel-chr13_10861 | 5p | 71 | | 159 | 0.4792899 |
| ssc-miR-novel-chr15_15523 | 3p | 8 | | 40 | 0.36 |
| ssc-miR-novel-chr16_17391 | 5p | 11 | | 33 | 0.4883721 |
| ssc-miR-novel-chr16_17392 | 3p | 18 | | 65 | 0.3733333 |
| ssc-miR-novel-chr16_17559 | 5p | 30 | | 234 | 0.1639344 |
| ssc-miR-novel-chr16_17561 | 5p | 4 | | 38 | 0.2916667 |
| ssc-miR-novel-chr1_1972 | 5p | 2 | | 15 | 0.48 |
| ssc-miR-novel-chr2_21624 | 5p | 3843 | | 14104 | 0.2729914 |
| ssc-miR-novel-chr5_29676 | 3p | 4 | | 19 | 0.4827586 |
| ssc-miR-novel-chr6_30857 | 3p | 4 | | 26 | 0.3888889 |
| ssc-miR-novel-chr6_32385 | 5p | 2 | | 15 | 0.48 |
| ssc-miR-novel-chr9_38827 | 5p | 2 | | 26 | 0.3333333 |
| ssc-miR-novel-JH118654-1_42487 | 5p | 30 | | 234 | 0.1639344 |

**Up-regulated miRNAs in PCMV infected spleen**

| **MATURE-ID** | **pre-miRNA arm (5p or 3p)** | **Spleen-Virus** | **Spleen-Control** | **Spleen-Virus vs Spleen-Control** |
| --- | --- | --- | --- | --- |
| ssc-let-7a | 5p | 17627 | 6553 | 2.6873381 |
| ssc-let-7a | 5p | 17627 | 6553 | 2.6873381 |
| ssc-let-7c | 5p | 2388 | 918 | 2.5840517 |
| ssc-let-7d-5p | 5p | 885 | 141 | 5.9271523 |
| ssc-let-7f | 5p | 38083 | 13734 | 2.7716094 |
| ssc-let-7f | 5p | 38083 | 13734 | 2.7716094 |
| ssc-miR-100 | 5p | 476 | 71 | 6 |
| ssc-miR-106a | 5p | 276 | 49 | 4.8474576 |
| ssc-miR-122 | 5p | 865 | 207 | 4.0322581 |
| ssc-miR-127 | 3p | 26 | 3 | 2.7692308 |
| ssc-miR-15b | 5p | 481 | 99 | 4.5045872 |
| ssc-miR-17-5p | 5p | 905 | 301 | 2.9421222 |
| ssc-miR-17-3p | 3p | 38 | 2 | 4 |
| ssc-miR-18a | 5p | 22 | 4 | 2.2857143 |
| ssc-miR-195 | 5p | 1101 | 86 | 11.572917 |
| ssc-miR-19a | 3p | 233 | 33 | 5.6511628 |
| ssc-miR-19b | 3p | 807 | 142 | 5.375 |
| ssc-miR-19b | 3p | 807 | 142 | 5.375 |
| ssc-miR-202-5p | 5p | 74 |  | 8.4 |
| ssc-miR-20a | 5p | 1493 | 372 | 3.934555 |
| ssc-miR-20b | 5p | 169 | 68 | 2.2948718 |
| ssc-miR-20b | 5p | 169 | 68 | 2.2948718 |
| ssc-miR-214 | 3p | 1715 | 33 | 40.116279 |
| ssc-miR-218-5p | 5p | 96 | 39 | 2.1632653 |
| ssc-miR-218 | 5p | 96 | 39 | 2.1632653 |
| ssc-miR-218b | 5p | 96 | 39 | 2.1632653 |
| ssc-miR-23a | 3p | 431 | 137 | 3 |
| ssc-miR-23b | 3p | 243 | 94 | 2.4326923 |
| ssc-miR-24-3p | 3p | 12094 | 671 | 17.773862 |
| ssc-miR-24-3p | 3p | 12094 | 671 | 17.773862 |
| ssc-miR-28-5p | 5p | 233 | 80 | 2.7 |
| ssc-miR-296-3p | 3p | 48 | 13 | 2.5217391 |
| ssc-miR-29c | 3p | 56 | 12 | 3 |
| ssc-miR-301 | 3p | 12 | 1 | 2 |
| ssc-miR-30e-3p | 3p | 709 | 250 | 2.7653846 |
| ssc-miR-361-5p | 5p | 38 | 3 | 3.6923077 |
| ssc-miR-3613 | 5p | 102 | 41 | 2.1960784 |
| ssc-miR-369 | 3p | 14 |  | 2.4 |
| ssc-miR-374b-3p | 3p | 33 | 8 | 2.3888889 |
| ssc-miR-421-3p | 3p | 63 | 8 | 4.0555556 |
| ssc-miR-423-5p | 5p | 259 | 120 | 2.0692308 |
| ssc-miR-92b-3p | 3p | 327 | 129 | 2.4244604 |
| ssc-miR-99a | 5p | 1037 | 180 | 5.5105263 |
| ssc-miR-novel-chr12_7961 | 3p | 325 | 137 | 2.2789116 |
| ssc-miR-novel-chr16_17391 | 5p | 182 | 43 | 3.6226415 |
| ssc-miR-novel-chr16_17392 | 3p | 302 | 125 | 2.3111111 |
| ssc-miR-novel-chr17_18987 | 5p | 48 | 13 | 2.5217391 |
| ssc-miR-novel-chrX_39952 | 5p | 499 | 223 | 2.1845494 |
| ssc-miR-novel-JH118656-1_42504 | 5p | 37 | 10 | 2.35 |

**Down-regulated miRNAs in PCMV infected spleen**

| **MATURE-ID** | **pre-miRNA arm (5p or 3p)** | **Spleen-Virus** | **Spleen-Control** | **Spleen-Virus vs Spleen-Control** |
| --- | --- | --- | --- | --- |
| ssc-miR-1 | 3p | 126 | 2225 | 0.0608501 |
| ssc-miR-103 | 3p | 210 | 890 | 0.2444444 |
| ssc-miR-103 | 3p | 210 | 890 | 0.2444444 |
| ssc-miR-107 | 3p | 13 | 108 | 0.1949153 |
| ssc-miR-125a | 5p | 132 | 313 | 0.4396285 |
| ssc-miR-125b | 5p | 31 | 113 | 0.3333333 |
| ssc-miR-125b | 5p | 31 | 113 | 0.3333333 |
| ssc-miR-126-5p | 5p | 1614 | 5018 | 0.3229912 |
| ssc-miR-140-5p | 5p | 4 | 37 | 0.2978723 |
| ssc-miR-142-5p | 5p | 1304 | 2857 | 0.4583188 |
| ssc-miR-142-3p | 3p | 22 | 66 | 0.4210526 |
| ssc-miR-146b | 5p | 4802 | 12746 | 0.3772342 |
| ssc-miR-148a-5p | 5p | 56 | 135 | 0.4551724 |
| ssc-miR-150 | 5p | 58 | 201 | 0.3222749 |
| ssc-miR-150 | 5p | 58 | 201 | 0.3222749 |
| ssc-miR-151-3p | 3p | 216 | 583 | 0.381113 |
| ssc-miR-186 | 5p | 653 | 2238 | 0.2949288 |
| ssc-miR-191 | 5p | 2265 | 7015 | 0.3238434 |
| ssc-miR-199a-3p | 3p | 432 | 1911 | 0.2300885 |
| ssc-miR-199a-3p | 3p | 432 | 1911 | 0.2300885 |
| ssc-miR-199b-3p | 3p | 432 | 1911 | 0.2300885 |
| ssc-miR-206 | 3p | 4 | 21 | 0.4516129 |
| ssc-miR-218-3p | 3p |  | 14 | 0.4166667 |
| ssc-miR-221-3p | 3p | 124 | 578 | 0.2278912 |
| ssc-miR-222 | 3p | 17 | 52 | 0.4354839 |
| ssc-miR-27b-3p | 3p | 1070 | 4563 | 0.2361688 |
| ssc-miR-29a | 3p | 156 | 423 | 0.3833718 |
| ssc-miR-30d | 5p | 2427 | 6036 | 0.4030764 |
| ssc-miR-30e-5p | 5p | 2284 | 6821 | 0.335822 |
| ssc-miR-378 | 3p | 1141 | 2473 | 0.4635522 |
| ssc-miR-378 | 3p | 1141 | 2473 | 0.4635522 |
| ssc-miR-411 | 3p | 3 | 40 | 0.26 |
| ssc-miR-425-5p | 5p | 151 | 4221 | 0.0380525 |
| ssc-miR-486 | 3p | 218 | 450 | 0.4956522 |
| ssc-miR-486 | 5p | 218 | 450 | 0.4956522 |
| ssc-miR-497 | 5p | 12 | 38 | 0.4583333 |
| ssc-miR-92a | 3p | 1979 | 3991 | 0.4971257 |
| ssc-miR-92a | 3p | 1979 | 3991 | 0.4971257 |
| ssc-miR-99b | 5p | 68 | 279 | 0.2698962 |
| ssc-miR-novel-chr12_8771 | 5p | 18 | 68 | 0.3589744 |
| ssc-miR-novel-chr15_15523 | 3p | 9 | 34 | 0.4318182 |
| ssc-miR-novel-chr16_17559 | 5p | 42 | 294 | 0.1710526 |
| ssc-miR-novel-chr5_29676 | 3p | 2 | 18 | 0.4285714 |
| ssc-miR-novel-chrX_40705 | 5p | 124 | 578 | 0.2278912 |
| ssc-miR-novel-GL892871-2_41708 | 5p | 2347 | 5459 | 0.4309746 |
| ssc-miR-novel-JH118654-1_42487 | 5p | 42 | 294 | 0.1710526 |

**Up-regulated miRNAs in PCMV infected liver**

| **MATURE-ID** | **pre-miRNA arm (5p or 3p)** | **Liver-PCMV** | **Liver-Control** | **Liver-PCMV vs Liver-Control** |
| --- | --- | --- | --- | --- |
| ssc-miR-101 | 3p | 3967 | 1307 | 3.0197418 |
| ssc-miR-101 | 3p | 3967 | 1307 | 3.0197418 |
| ssc-miR-107 | 3p | 42 | 15 | 2.08 |
| ssc-miR-10a-3p | 3p | 20 |  | 3 |
| ssc-miR-122 | 5p | 182882 | 25749 | 7.1001203 |
| ssc-miR-126-3p | 3p | 5953 | 1225 | 4.8283401 |
| ssc-miR-128 | 3p | 134 | 20 | 4.8 |
| ssc-miR-128 | 3p | 134 | 20 | 4.8 |
| ssc-miR-139-5p | 5p | 168 | 29 | 4.5641026 |
| ssc-miR-155-5p | 5p | 441 | 123 | 3.3909774 |
| ssc-miR-199a-5p | 5p | 397 | 27 | 11 |
| ssc-miR-199a-5p | 5p | 397 | 27 | 11 |
| ssc-miR-199b-5p | 5p | 33 | 2 | 3.5833333 |
| ssc-miR-26a | 5p | 38895 | 11065 | 3.5128668 |
| ssc-miR-27a | 3p | 306 | 115 | 2.528 |
| ssc-miR-27b-3p | 3p | 5155 | 1127 | 4.5426561 |
| ssc-miR-361-3p | 3p | 18 | 4 | 2 |
| ssc-miR-424-3p | 3p | 30 | 4 | 2.8571429 |
| ssc-miR-497 | 5p | 81 | 20 | 3.0333333 |
| ssc-miR-novel-chr10_5602 | 5p | 28 |  | 3.8 |
| ssc-miR-novel-chr14_13301 | 3p | 98 | 43 | 2.0377358 |
| ssc-miR-novel-GL892871-2_41708 | 5p | 10317 | 4143 | 2.4866362 |

**Down-regulated miRNAs in PCMV infected liver**

| **MATURE-ID** | **pre-miRNA arm (5p or 3p)** | **Liver-PCMV** | **Liver-Control** | **Liver-PCMV vs Liver-Control** |
| --- | --- | --- | --- | --- |
| ssc-let-7a | 5p | 7513 | 29274 | 0.256898 |
| ssc-let-7a | 5p | 7513 | 29274 | 0.256898 |
| ssc-let-7c | 5p | 942 | 2226 | 0.4257603 |
| ssc-let-7d-5p | 5p | 56 | 1194 | 0.0548173 |
| ssc-let-7e | 5p | 207 | 527 | 0.4040968 |
| ssc-let-7f | 5p | 11968 | 47250 | 0.253449 |
| ssc-let-7f | 5p | 11968 | 47250 | 0.253449 |
| ssc-let-7g | 5p | 4722 | 18351 | 0.2577202 |
| ssc-miR-106a | 5p | 23 | 62 | 0.4583333 |
| ssc-miR-142-5p | 5p | 128 | 286 | 0.4662162 |
| ssc-miR-143-3p | 3p | 12500 | 29398 | 0.4253945 |
| ssc-miR-145-5p | 5p | 87 | 336 | 0.2803468 |
| ssc-miR-146a-5p | 5p | 44 | 279 | 0.1868512 |
| ssc-miR-146b | 5p | 281 | 880 | 0.3269663 |
| ssc-miR-150 | 5p | 2 | 14 | 0.5 |
| ssc-miR-150 | 5p | 2 | 14 | 0.5 |
| ssc-miR-151-5p | 5p | 185 | 425 | 0.4482759 |
| ssc-miR-15a | 5p | 66 | 195 | 0.3707317 |
| ssc-miR-15b | 5p | 28 | 200 | 0.1809524 |
| ssc-miR-16 | 5p | 3209 | 7253 | 0.4432053 |
| ssc-miR-16 | 5p | 3209 | 7253 | 0.4432053 |
| ssc-miR-17-3p | 3p | 10 | 33 | 0.4651163 |
| ssc-miR-181a | 5p | 2402 | 8675 | 0.2777202 |
| ssc-miR-181a | 5p | 2402 | 8675 | 0.2777202 |
| ssc-miR-181b | 5p | 207 | 601 | 0.3551555 |
| ssc-miR-181b | 5p | 207 | 601 | 0.3551555 |
| ssc-miR-181c | 5p | 69 | 166 | 0.4488636 |
| ssc-miR-181d-5p | 5p | 34 | 92 | 0.4313725 |
| ssc-miR-182 | 5p | 88 | 260 | 0.362963 |
| ssc-miR-186 | 5p | 189 | 644 | 0.3042813 |
| ssc-miR-194a | 5p | 101 | 660 | 0.1656716 |
| ssc-miR-194b-5p | 5p |  | 10 | 0.5 |
| ssc-miR-195 | 5p | 53 | 2000 | 0.0313433 |
| ssc-miR-19a | 3p | 6 | 271 | 0.0569395 |
| ssc-miR-19b | 3p | 55 | 1471 | 0.0438893 |
| ssc-miR-19b | 3p | 55 | 1471 | 0.0438893 |
| ssc-miR-202-5p | 5p |  | 10 | 0.5 |
| ssc-miR-204 | 5p | 71 | 398 | 0.1985294 |
| ssc-miR-21 | 5p | 10140 | 21162 | 0.4794068 |
| ssc-miR-214 | 3p | 52 | 2786 | 0.0221745 |
| ssc-miR-22-3p | 3p | 1420 | 3388 | 0.4208358 |
| ssc-miR-222 | 3p | 14 | 75 | 0.2823529 |
| ssc-miR-23a | 3p | 28 | 201 | 0.1800948 |
| ssc-miR-23b | 3p | 39 | 190 | 0.245 |
| ssc-miR-24-3p | 3p | 335 | 11321 | 0.0304474 |
| ssc-miR-24-3p | 3p | 335 | 11321 | 0.0304474 |
| ssc-miR-28-5p | 5p | 22 | 234 | 0.1311475 |
| ssc-miR-296-3p | 3p | 4 | 47 | 0.245614 |
| ssc-miR-29a | 3p | 81 | 312 | 0.2826087 |
| ssc-miR-301 | 3p |  | 10 | 0.5 |
| ssc-miR-30a-5p | 5p | 31475 | 70405 | 0.4471348 |
| ssc-miR-30a-3p | 3p | 146 | 347 | 0.4369748 |
| ssc-miR-30b-5p | 5p | 250 | 1208 | 0.2134647 |
| ssc-miR-30c-5p | 5p | 279 | 1005 | 0.2847291 |
| ssc-miR-30c-5p | 5p | 279 | 1005 | 0.2847291 |
| ssc-miR-30e-3p | 3p | 183 | 586 | 0.3238255 |
| ssc-miR-335 | 5p | 6 | 35 | 0.3555556 |
| ssc-miR-340 | 5p | 117 | 246 | 0.4960938 |
| ssc-miR-340 | 5p | 117 | 246 | 0.4960938 |
| ssc-miR-342 | 3p | 11 | 49 | 0.3559322 |
| ssc-miR-361-5p | 5p |  | 33 | 0.2325581 |
| ssc-miR-3613 | 5p | 21 | 63 | 0.4246575 |
| ssc-miR-374a-5p | 5p | 100 | 291 | 0.3654485 |
| ssc-miR-374b-3p | 3p | 2 | 29 | 0.3076923 |
| ssc-miR-421-3p | 3p |  | 30 | 0.25 |
| ssc-miR-423-5p | 5p | 88 | 288 | 0.3288591 |
| ssc-miR-423-3p | 3p | 51 | 152 | 0.3765432 |
| ssc-miR-424-5p | 5p | 16 | 69 | 0.3291139 |
| ssc-miR-451 | 5p | 185 | 454 | 0.4202586 |
| ssc-miR-542-3p | 3p | 61 | 300 | 0.2290323 |
| ssc-miR-874 | 3p | 11 | 37 | 0.4468085 |
| ssc-miR-885-3p | 3p | 35 | 81 | 0.4945055 |
| ssc-miR-92b-3p | 3p | 20 | 153 | 0.1840491 |
| ssc-miR-novel-chr12_7961 | 3p | 67 | 149 | 0.4842767 |
| ssc-miR-novel-chr12_7963 | 3p | 3 | 21 | 0.4193548 |
| ssc-miR-novel-chr12_7964 | 5p | 3 | 21 | 0.4193548 |
| ssc-miR-novel-chr13_10658 | 5p | 2 | 51 | 0.1967213 |
| ssc-miR-novel-chr16_17391 | 5p | 54 | 170 | 0.3555556 |
| ssc-miR-novel-chr16_17392 | 3p | 110 | 287 | 0.4040404 |
| ssc-miR-novel-chr17_18987 | 5p | 4 | 47 | 0.245614 |
| ssc-miR-novel-chr2_21624 | 5p | 12500 | 29398 | 0.4253945 |
| ssc-miR-novel-chr3_23271 | 3p | 19 | 64 | 0.3918919 |
| ssc-miR-novel-chrX_39952 | 5p | 44 | 184 | 0.2783505 |
| ssc-miR-novel-GL896425-1_44856 | 3p | 187 | 1582 | 0.1237437 |
| ssc-miR-novel-JH118656-1_42504 | 5p | 3 | 20 | 0.4333333 |

**Table S4. Target prediction for DE miRNAs.**

**Target prediction of miR-148a-3p**

**Target prediction of miR-27b-3p**

**Target prediction of miR-10a-5p**

**Target prediction of miR-143-3p**

**Target prediction of miR-30a-5p**

**Target prediction of miR-novel-chr2-21624**

**Target prediction of let-7g**

**Target prediction of miR-10b**

**Target prediction of miR-155-5p**

**Target prediction of miR-16**

**Target prediction of miR-17-5p**

**Target prediction of miR-20a**

**Table S5. Integrated expression analysis of DE miRNAs and their target mRNA**

| M miRNAs | DE miRNA Fold change value | Target mRNAs | Target mRNA Fold change value |
| --- | --- | --- | --- |
| ssc-miR-148a-3p | 2.462659381 | STAT5B | -2.5524285 |
| ssc-miR-148a-3p | 2.462659381 | DCT | -2.157282 |
| ssc-miR-148a-3p | 2.462659381 | CD1D | -2.7104983 |
| ssc-miR-148a-3p | 2.462659381 | NPEPL1 | -3.5366943 |
| ssc-miR-148a-3p | 2.462659381 | SRSF2 | -3.7033532 |
| ssc-miR-148a-3p | 2.462659381 | ZNF76 | -2.7170002 |
| ssc-miR-148a-3p | 2.462659381 | TXNRD1 | -2.9226952 |
| ssc-miR-148a-3p | 2.462659381 | POLR2B | -2.5943034 |
| ssc-miR-148a-3p | 2.462659381 | SRSF2 | -2.8172517 |
| ssc-miR-148a-3p | 2.462659381 | POLR2B | -2.452575 |
| ssc-miR-148a-3p | 2.462659381 | NFYB | -2.2796273 |
| ssc-miR-148a-3p | 2.462659381 | IREB2 | -4.057979 |
| ssc-miR-148a-3p | 2.462659381 | CPT1A | -2.307164 |
| ssc-miR-148a-3p | 2.462659381 | USP7 | -3.1586738 |
| ssc-miR-148a-3p | 2.462659381 | NFATC2 | -2.687189 |
| ssc-miR-148a-3p | 2.462659381 | STAT5B | -3.8813813 |
| ssc-miR-148a-3p | 2.462659381 | IDE | -2.164203 |
| ssc-miR-148a-3p | 2.462659381 | CLK1 | -2.1953208 |
| ssc-miR-148a-3p | 2.462659381 | SELL | -5.146434 |
| ssc-miR-148a-3p | 2.462659381 | USF1 | -2.006216 |
| ssc-miR-148a-3p | 2.462659381 | PARP11 | -3.0731509 |
| ssc-miR-148a-3p | 2.462659381 | TRIM26 | -2.0870156 |
| ssc-miR-148a-3p | 2.462659381 | PLAT | -2.1457462 |
| ssc-miR-148a-3p | 2.462659381 | PN-1 | -2.255461 |
| ssc-miR-148a-3p | 2.462659381 | OGT | -2.2657173 |
| ssc-miR-148a-3p | 2.462659381 | MKNK1 | -3.1399946 |
| ssc-miR-148a-3p | 2.462659381 | SRSF2 | -3.3484426 |
| ssc-miR-148a-3p | 2.462659381 | MMD | -5.547919 |
| ssc-miR-148a-3p | 2.462659381 | OGT | -3.4658635 |
| ssc-miR-148a-3p | 2.462659381 | CD1D | -6.473435 |
| ssc-miR-148a-3p | 2.462659381 | TRIM26 | -2.2679245 |
| ssc-miR-148a-3p | 2.462659381 | APEX1 | -3.620095 |
| ssc-miR-148a-3p | 2.462659381 | USP18 | -2.800909 |
| ssc-miR-148a-3p | 2.462659381 | ACO2 | -3.947841 |
| ssc-miR-148a-3p | 2.462659381 | CCND3 | -3.4445536 |
| ssc-miR-148a-3p | 2.462659381 | KLF13 | -2.8979645 |
| ssc-miR-148a-3p | 2.462659381 | POT1 | -2.0162158 |
| ssc-miR-148a-3p | 2.462659381 | SELL | -7.825198 |
| ssc-miR-148a-3p | 2.462659381 | CLK1 | -2.1448402 |
| ssc-miR-148a-3p | 2.462659381 | EPS15 | -2.600187 |
| ssc-miR-148a-3p | 2.462659381 | SRSF2 | -3.353023 |
| ssc-miR-148a-3p | 2.462659381 | PPP1CB | -2.1159654 |
| ssc-miR-148a-3p | 2.462659381 | USP47 | -3.5484018 |
| ssc-miR-148a-3p | 2.462659381 | CD1D | -2.2370274 |
| ssc-miR-148a-3p | 2.462659381 | PPP1CB | -2.0893292 |
| ssc-miR-148a-3p | 2.462659381 | EPS15 | -2.5533533 |
| ssc-miR-148a-3p | 2.462659381 | DNMT1 | -3.2535224 |
| ssc-miR-148a-3p | 2.462659381 | CD4 | -7.3410873 |
| ssc-miR-148a-3p | 2.462659381 | DNMT1 | -2.4039989 |
| ssc-miR-148a-3p | 2.462659381 | APEX1 | -3.6698933 |
| ssc-miR-148a-3p | 2.462659381 | OGT | -4.6648674 |
| ssc-miR-148a-3p | 2.462659381 | USF1 | -5.137944 |
| ssc-miR-148a-3p | 2.462659381 | ZNF217 | -5.367244 |
| ssc-miR-148a-3p | 2.462659381 | RNF114 | -2.999979 |
| ssc-miR-148a-3p | 2.462659381 | TXNRD2 | -3.0435395 |
| ssc-miR-148a-3p | 2.462659381 | PPP1CB | -3.6685545 |
| ssc-miR-148a-3p | 2.462659381 | CXCL12 | -4.46094 |
| ssc-miR-148a-3p | 2.462659381 | NFATC2 | -3.1316254 |
| ssc-miR-148a-3p | 2.462659381 | PTPN1 | -2.4280972 |
| ssc-miR-148a-3p | 2.462659381 | SH2B3 | -2.0666575 |
| ssc-miR-148a-3p | 2.462659381 | ARHGAP25 | -2.224952 |
| ssc-miR-148a-3p | 2.462659381 | Scarb2 | -2.121959 |
| ssc-miR-148a-3p | 2.462659381 | STAT1 | -2.6724877 |
| ssc-miR-148a-3p | 2.462659381 | XPO7 | -2.1687493 |
| ssc-miR-148a-3p | 2.462659381 | LEF1 | -14.021283 |
| ssc-miR-148a-3p | 2.462659381 | Scarb2 | -2.126361 |
| ssc-miR-148a-3p | 2.462659381 | TRMT6 | -2.7288475 |
| ssc-miR-148a-3p | 2.462659381 | MTF2 | -3.5220673 |
| ssc-miR-148a-3p | 2.462659381 | DCT | -2.534571 |
| ssc-miR-148a-3p | 2.462659381 | PN-1 | -2.4372778 |
| ssc-miR-148a-3p | 2.462659381 | STAT1 | -2.5751772 |
| ssc-miR-148a-3p | 2.462659381 | SRSF2 | -2.0046167 |
| ssc-miR-148a-3p | 2.462659381 | ATG4D | -3.3044126 |
| ssc-miR-148a-3p | 2.462659381 | SELE | -7.275607 |
| ssc-miR-148a-3p | 2.462659381 | LY75 | -3.0788105 |
| ssc-miR-148a-3p | 2.462659381 | ITPK1 | -2.6786532 |
| ssc-miR-148a-3p | 2.462659381 | ATL3 | -3.9312913 |
| ssc-miR-148a-3p | 2.462659381 | SLC29A1 | -2.810364 |
| ssc-miR-148a-3p | 2.462659381 | XIAP | -2.8055277 |
| ssc-miR-148a-3p | 2.462659381 | PIKFYVE | -2.862979 |
| ssc-miR-148a-3p | 2.462659381 | CXCL12 | -4.690473 |
| ssc-miR-148a-3p | 2.462659381 | OGT | -6.4931555 |
| ssc-miR-148a-3p | 2.462659381 | APEX1 | -2.301916 |
| ssc-miR-148a-3p | 2.462659381 | LEF1 | -13.521802 |
| ssc-miR-148a-3p | 2.462659381 | PLAT | -2.096734 |
| ssc-miR-148a-3p | 2.462659381 | NPEPL1 | -2.8677049 |
| ssc-miR-148a-3p | 2.462659381 | STAT5B | -2.5524285 |
| ssc-miR-148a-3p | 2.462659381 | CORO1C | -2.9706523 |
| ssc-miR-148a-3p | 2.462659381 | DNMT1 | -3.1524034 |
| ssc-miR-148a-3p | 2.462659381 | DNMT1 | -3.2025714 |
| ssc-miR-148a-3p | 2.462659381 | MMD | -3.0891557 |
| ssc-miR-148a-3p | 2.462659381 | XIAP | -2.120111 |
| ssc-miR-148a-3p | 2.462659381 | BCL9 | BCL9 |
| ssc-miR-148a-3p | 2.462659381 | WBP11 | -2.375831 |
| ssc-miR-148a-3p | 2.462659381 | TSPO | -2.0712407 |
| ssc-miR-148a-3p | 2.462659381 | PPP1CB | -2.4047458 |
| ssc-miR-148a-3p | 2.462659381 | KDM2A | -2.2741494 |
| ssc-miR-148a-3p | 2.462659381 | CD8A | -2.7839508 |
| ssc-miR-148a-3p | 2.462659381 | STAT1 | -3.216691 |
| ssc-miR-148a-3p | 2.462659381 | SRSF2 | -4.9495416 |
| ssc-miR-148a-3p | 2.462659381 | APEX1 | -3.3851306 |
| ssc-miR-148a-3p | 2.462659381 | CD8A | -5.032429 |
| ssc-miR-148a-3p | 2.462659381 | SRSF2 | -3.1398275 |
| ssc-miR-148a-3p | 2.462659381 | RPL30 | -2.3963418 |
| ssc-miR-148a-3p | 2.462659381 | SELL | -3.6556106 |
| ssc-miR-148a-3p | 2.462659381 | TXNRD1 | -2.0018742 |
| ssc-miR-148a-3p | 2.462659381 | MEOX2 | -3.7749765 |
|  |  |  |  |
| ssc-miR-27b-3p | 4.837664934 | JAK2 | -2.1547365 |
| ssc-miR-27b-3p | 4.837664934 | CCL8 | -3.1992505 |
| ssc-miR-27b-3p | 4.837664934 | CSN2 | -2.4015813 |
| ssc-miR-27b-3p | 4.837664934 | DCT | -2.157282 |
| ssc-miR-27b-3p | 4.837664934 | NOC3L | -3.3583956 |
| ssc-miR-27b-3p | 4.837664934 | TUBA1B | -2.2096076 |
| ssc-miR-27b-3p | 4.837664934 | SRSF2 | -3.7033532 |
| ssc-miR-27b-3p | 4.837664934 | PRPF40A | -3.7033532 |
| ssc-miR-27b-3p | 4.837664934 | PRPF40A | -2.0117452 |
| ssc-miR-27b-3p | 4.837664934 | NME1 | -5.8544197 |
| ssc-miR-27b-3p | 4.837664934 | NIP7 | -2.8710716 |
| ssc-miR-27b-3p | 4.837664934 | SRSF2 | -2.8172517 |
| ssc-miR-27b-3p | 4.837664934 | LPIN1 | -2.1717892 |
| ssc-miR-27b-3p | 4.837664934 | AKAP13 | -2.0148356 |
| ssc-miR-27b-3p | 4.837664934 | ITGA4 | -2.4564707 |
| ssc-miR-27b-3p | 4.837664934 | IREB2 | -4.057979 |
| ssc-miR-27b-3p | 4.837664934 | CD2 | -6.0158634 |
| ssc-miR-27b-3p | 4.837664934 | MCM3 | -3.3112478 |
| ssc-miR-27b-3p | 4.837664934 | SLAMF6 | -9.264798 |
| ssc-miR-27b-3p | 4.837664934 | NLN | -2.160635 |
| ssc-miR-27b-3p | 4.837664934 | IDE | -2.164203 |
| ssc-miR-27b-3p | 4.837664934 | TCF19 | -7.779283 |
| ssc-miR-27b-3p | 4.837664934 | CLK1 | -2.1953208 |
| ssc-miR-27b-3p | 4.837664934 | WNK1 | -2.4279263 |
| ssc-miR-27b-3p | 4.837664934 | NOL9 | -2.7997262 |
| ssc-miR-27b-3p | 4.837664934 | CD19 | -8.638682 |
| ssc-miR-27b-3p | 4.837664934 | B3GALNT1 | -5.3493724 |
| ssc-miR-27b-3p | 4.837664934 | USF1 | -2.006216 |
| ssc-miR-27b-3p | 4.837664934 | NCK1 | -2.0576825 |
| ssc-miR-27b-3p | 4.837664934 | NCK1-1123行 | -7.2229953 |
| ssc-miR-27b-3p | 4.837664934 | FOXO1 | -4.3853016 |
| ssc-miR-27b-3p | 4.837664934 | SLA | -3.10224 |
| ssc-miR-27b-3p | 4.837664934 | MKNK1 | -3.1399946 |
| ssc-miR-27b-3p | 4.837664934 | SRSF2 | -3.3484426 |
| ssc-miR-27b-3p | 4.837664934 | MMD | -5.547919 |
| ssc-miR-27b-3p | 4.837664934 | GNAS | -2.8643892 |
| ssc-miR-27b-3p | 4.837664934 | ST6GALNAC2 | -6.3674126 |
| ssc-miR-27b-3p | 4.837664934 | STMN2 | -17.846478 |
| ssc-miR-27b-3p | 4.837664934 | LPIN1 | -2.419083 |
| ssc-miR-27b-3p | 4.837664934 | STMN2 | -5.056418 |
| ssc-miR-27b-3p | 4.837664934 | TCF19 | -4.051245 |
| ssc-miR-27b-3p | 4.837664934 | PPARG | -2.8084548 |
| ssc-miR-27b-3p | 4.837664934 | CLK1 | -2.1448402 |
| ssc-miR-27b-3p | 4.837664934 | SRSF2 | -3.353023 |
| ssc-miR-27b-3p | 4.837664934 | C-FLIP | -2.59968 |
| ssc-miR-27b-3p | 4.837664934 | DGCR8 | -3.5259063 |
| ssc-miR-27b-3p | 4.837664934 | NIP7 | -3.2928646 |
| ssc-miR-27b-3p | 4.837664934 | SRSF1 | -2.6913757 |
| ssc-miR-27b-3p | 4.837664934 | OVGP1 | -3.0902452 |
| ssc-miR-27b-3p | 4.837664934 | PIK3CG | -2.769445 |
| ssc-miR-27b-3p | 4.837664934 | USF1 | -5.137944 |
| ssc-miR-27b-3p | 4.837664934 | CDC42 | -2.1404748 |
| ssc-miR-27b-3p | 4.837664934 | GLTP | -2.5359585 |
| ssc-miR-27b-3p | 4.837664934 | MX1 | -2.3029873 |
| ssc-miR-27b-3p | 4.837664934 | WNK1 | -3.6181035 |
| ssc-miR-27b-3p | 4.837664934 | DPYD | -2.1470616 |
| ssc-miR-27b-3p | 4.837664934 | CXCL12 | -4.46094 |
| ssc-miR-27b-3p | 4.837664934 | B3GALNT1 | -2.8551302 |
| ssc-miR-27b-3p | 4.837664934 | Scarb2 | -2.121959 |
| ssc-miR-27b-3p | 4.837664934 | GLS | -3.1439273 |
| ssc-miR-27b-3p | 4.837664934 | Scarb2 | -2.126361 |
| ssc-miR-27b-3p | 4.837664934 | MX1 | -2.941669 |
| ssc-miR-27b-3p | 4.837664934 | DIABLO | -2.8235626 |
| ssc-miR-27b-3p | 4.837664934 | SFXN1 | -2.7520895 |
| ssc-miR-27b-3p | 4.837664934 | NR2F2 | -2.2847369 |
| ssc-miR-27b-3p | 4.837664934 | MTF2 | -3.5220673 |
| ssc-miR-27b-3p | 4.837664934 | DCT | -2.534571 |
| ssc-miR-27b-3p | 4.837664934 | DHX58 | -4.9549723 |
| ssc-miR-27b-3p | 4.837664934 | SRSF1 | -2.7404566 |
| ssc-miR-27b-3p | 4.837664934 | C-FLIP | -2.0778258 |
| ssc-miR-27b-3p | 4.837664934 | SRSF2 | -2.0046167 |
| ssc-miR-27b-3p | 4.837664934 | GNAS | -2.043067 |
| ssc-miR-27b-3p | 4.837664934 | ARHGAP30 | -2.1464188 |
| ssc-miR-27b-3p | 4.837664934 | ARID4A | -2.4397392 |
| ssc-miR-27b-3p | 4.837664934 | SELE | -7.275607 |
| ssc-miR-27b-3p | 4.837664934 | PSEN1 | -2.1231768 |
| ssc-miR-27b-3p | 4.837664934 | JAK2 | -2.1547365 |
| ssc-miR-27b-3p | 4.837664934 | LPIN1 | -3.5409367 |
| ssc-miR-27b-3p | 4.837664934 | ATL3 | -3.9312913 |
| ssc-miR-27b-3p | 4.837664934 | CD2 | -5.586634 |
| ssc-miR-27b-3p | 4.837664934 | SLA | -3.0471833 |
| ssc-miR-27b-3p | 4.837664934 | MX1 | -6.9861736 |
| ssc-miR-27b-3p | 4.837664934 | CCL8 | -2.548812 |
| ssc-miR-27b-3p | 4.837664934 | CXCL12 | -4.690473 |
| ssc-miR-27b-3p | 4.837664934 | RAI14 | -2.5591626 |
| ssc-miR-27b-3p | 4.837664934 | STMN2 | -11.529461 |
| ssc-miR-27b-3p | 4.837664934 | RPS16 | -2.7945027 |
| ssc-miR-27b-3p | 4.837664934 | SRSF1 | -2.0801952 |
| ssc-miR-27b-3p | 4.837664934 | MMD | -3.0891557 |
| ssc-miR-27b-3p | 4.837664934 | PGK1 | -2.7207043 |
| ssc-miR-27b-3p | 4.837664934 | COPZ1 | -2.0277896 |
| ssc-miR-27b-3p | 4.837664934 | C-FLIP | -2.2538774 |
| ssc-miR-27b-3p | 4.837664934 | FOXO1 | -3.7203667 |
| ssc-miR-27b-3p | 4.837664934 | CCL8 | -3.1992505 |
| ssc-miR-27b-3p | 4.837664934 | MU | -2.0744867 |
| ssc-miR-27b-3p | 4.837664934 | KDM2A | -2.2741494 |
| ssc-miR-27b-3p | 4.837664934 | ORAI1 | -3.7655594 |
| ssc-miR-27b-3p | 4.837664934 | SRSF2 | -4.9495416 |
| ssc-miR-27b-3p | 4.837664934 | TMOD3 | -2.4760551 |
| ssc-miR-27b-3p | 4.837664934 | FOXO1 | -4.668094 |
| ssc-miR-27b-3p | 4.837664934 | C-FLIP | -2.3288872 |
| ssc-miR-27b-3p | 4.837664934 | SRSF2 | -3.1398275 |
| ssc-miR-27b-3p | 4.837664934 | ARID4A | -3.5558572 |
|  |  |  |  |
| ssc-miR-10a-5p | 2.53317208 | TLR7 | -2.0132992 |
| ssc-miR-10a-5p | 2.53317208 | LPCAT4 | -2.5515091 |
| ssc-miR-10a-5p | 2.53317208 | TLR7 | -2.0132992 |
| ssc-miR-10a-5p | 2.53317208 | ABLIM1 | -2.2041342 |
| ssc-miR-10a-5p | 2.53317208 | SLC15A2 | -5.948693 |
| ssc-miR-10a-5p | 2.53317208 | ANGPTL4 | -2.4502156 |
| ssc-miR-10a-5p | 2.53317208 | CYBRD1 | -2.0514493 |
| ssc-miR-10a-5p | 2.53317208 | UBQLN4 | -3.195487 |
| ssc-miR-10a-5p | 2.53317208 | SLC15A2 | -4.0057564 |
| ssc-miR-10a-5p | 2.53317208 | CD2 | -6.0158634 |
| ssc-miR-10a-5p | 2.53317208 | PCK1 | -2.0179653 |
| ssc-miR-10a-5p | 2.53317208 | PPP2R5D | -2.5809543 |
| ssc-miR-10a-5p | 2.53317208 | CAPRIN1 | -2.016781 |
| ssc-miR-10a-5p | 2.53317208 | NOL9 | -2.7997262 |
| ssc-miR-10a-5p | 2.53317208 | MAP3K7 | -3.955688 |
| ssc-miR-10a-5p | 2.53317208 | MMP11 | -2.1907008 |
| ssc-miR-10a-5p | 2.53317208 | ANGPTL4 | -2.108344 |
| ssc-miR-10a-5p | 2.53317208 | PROCR | -3.458278 |
| ssc-miR-10a-5p | 2.53317208 | PROCR | -3.9000366 |
| ssc-miR-10a-5p | 2.53317208 | SLC26A6 | -4.7701306 |
| ssc-miR-10a-5p | 2.53317208 | UHRF1 | -5.3087754 |
| ssc-miR-10a-5p | 2.53317208 | ACO2 | -3.947841 |
| ssc-miR-10a-5p | 2.53317208 | MPI | -2.10982 |
| ssc-miR-10a-5p | 2.53317208 | SLA-DOB | -11.237711 |
| ssc-miR-10a-5p | 2.53317208 | CTSK | -2.4026263 |
| ssc-miR-10a-5p | 2.53317208 | PPP1CB | -2.1159654 |
| ssc-miR-10a-5p | 2.53317208 | CTSK | -2.1884792 |
| ssc-miR-10a-5p | 2.53317208 | PPP1CB | -2.0893292 |
| ssc-miR-10a-5p | 2.53317208 | SRSF1 | -2.6913757 |
| ssc-miR-10a-5p | 2.53317208 | SAMHD1 | -6.521527 |
| ssc-miR-10a-5p | 2.53317208 | MAPRE1 | -2.695608 |
| ssc-miR-10a-5p | 2.53317208 | CTSK | -2.1976178 |
| ssc-miR-10a-5p | 2.53317208 | TLR6 | -2.8793592 |
| ssc-miR-10a-5p | 2.53317208 | PPP1CB | -3.6685545 |
| ssc-miR-10a-5p | 2.53317208 | ARHGAP25 | -2.224952 |
| ssc-miR-10a-5p | 2.53317208 | Scarb2 | -2.121959 |
| ssc-miR-10a-5p | 2.53317208 | PTEN | -2.4168127 |
| ssc-miR-10a-5p | 2.53317208 | Scarb2 | -2.126361 |
| ssc-miR-10a-5p | 2.53317208 | NR2F2 | -2.2847369 |
| ssc-miR-10a-5p | 2.53317208 | MTF2 | -3.5220673 |
| ssc-miR-10a-5p | 2.53317208 | SRSF1 | -2.7404566 |
| ssc-miR-10a-5p | 2.53317208 | CIT | -3.5340838 |
| ssc-miR-10a-5p | 2.53317208 | ADAM17 | -2.3873596 |
| ssc-miR-10a-5p | 2.53317208 | CD2 | -5.586634 |
| ssc-miR-10a-5p | 2.53317208 | ASF1B | -3.822117 |
| ssc-miR-10a-5p | 2.53317208 | PTEN | -2.5441053 |
| ssc-miR-10a-5p | 2.53317208 | PIKFYVE | -2.862979 |
| ssc-miR-10a-5p | 2.53317208 | PROCR | -6.8161187 |
| ssc-miR-10a-5p | 2.53317208 | CTLA4 | -7.234559 |
| ssc-miR-10a-5p | 2.53317208 | CDCA7 | -4.594474 |
| ssc-miR-10a-5p | 2.53317208 | SLC26A6 | -2.3229346 |
| ssc-miR-10a-5p | 2.53317208 | GZMH | -4.6076193 |
| ssc-miR-10a-5p | 2.53317208 | GABBR1 | -3.0064876 |
| ssc-miR-10a-5p | 2.53317208 | SRSF1 | -2.0801952 |
| ssc-miR-10a-5p | 2.53317208 | COPZ1 | -2.0277896 |
| ssc-miR-10a-5p | 2.53317208 | SAMHD1 | -4.093459 |
| ssc-miR-10a-5p | 2.53317208 | MAPRE1 | -3.5609426 |
| ssc-miR-10a-5p | 2.53317208 | ATP5L | -3.3654962 |
| ssc-miR-10a-5p | 2.53317208 | PPP1CB | -2.4047458 |
| ssc-miR-10a-5p | 2.53317208 | GNAI2 | -3.229197 |
| ssc-miR-10a-5p | 2.53317208 | CDCA7 | -6.263909 |
|  |  |  |  |
| ssc-miR-143-3p | 6.683255906 | DDX58 | -4.4436197 |
| ssc-miR-143-3p | 6.683255906 | CD40 | -2.6959412 |
| ssc-miR-143-3p | 6.683255906 | IL2RG | -5.07989 |
| ssc-miR-143-3p | 6.683255906 | CSN2 | -2.4015813 |
| ssc-miR-143-3p | 6.683255906 | FZR1 | -2.0416865 |
| ssc-miR-143-3p | 6.683255906 | NOC3L | -3.3583956 |
| ssc-miR-143-3p | 6.683255906 | SLA-DQA | -2.6939797 |
| ssc-miR-143-3p | 6.683255906 | NCOA3 | -2.3024986 |
| ssc-miR-143-3p | 6.683255906 | HR | -2.116809 |
| ssc-miR-143-3p | 6.683255906 | TXNRD1 | -2.9226952 |
| ssc-miR-143-3p | 6.683255906 | POLR2B | -2.5943034 |
| ssc-miR-143-3p | 6.683255906 | IL2RG | -4.2400565 |
| ssc-miR-143-3p | 6.683255906 | ENTPD1 | -2.0566626 |
| ssc-miR-143-3p | 6.683255906 | GATM | -2.5589132 |
| ssc-miR-143-3p | 6.683255906 | POLR2B | -2.452575 |
| ssc-miR-143-3p | 6.683255906 | USP47 | -3.5484018 |
| ssc-miR-143-3p | 6.683255906 | ATF7IP | -2.1011086 |
| ssc-miR-143-3p | 6.683255906 | ITGA4 | -2.4564707 |
| ssc-miR-143-3p | 6.683255906 | GATM | -2.213037 |
| ssc-miR-143-3p | 6.683255906 | CASQ1 | -4.4726872 |
| ssc-miR-143-3p | 6.683255906 | PBK | -2.210211 |
| ssc-miR-143-3p | 6.683255906 | NFATC2 | -2.687189 |
| ssc-miR-143-3p | 6.683255906 | DDX52 | -3.784038 |
| ssc-miR-143-3p | 6.683255906 | CAPRIN1 | -2.016781 |
| ssc-miR-143-3p | 6.683255906 | MAP3K7 | -3.955688 |
| ssc-miR-143-3p | 6.683255906 | MMP11 | -2.1907008 |
| ssc-miR-143-3p | 6.683255906 | IKBKB | -2.1580052 |
| ssc-miR-143-3p | 6.683255906 | GATM | -2.3272405 |
| ssc-miR-143-3p | 6.683255906 | MMD | -5.547919 |
| ssc-miR-143-3p | 6.683255906 | CD40 | -4.9732995 |
| ssc-miR-143-3p | 6.683255906 | GATM | -2.8943021 |
| ssc-miR-143-3p | 6.683255906 | CTSB | -2.3818676 |
| ssc-miR-143-3p | 6.683255906 | SLA-DOB | -11.237711 |
| ssc-miR-143-3p | 6.683255906 | CDK4 | -2.4161417 |
| ssc-miR-143-3p | 6.683255906 | KLF13 | -2.8979645 |
| ssc-miR-143-3p | 6.683255906 | CDK4 | -2.2926328 |
| ssc-miR-143-3p | 6.683255906 | PPARD | -2.3047228 |
| ssc-miR-143-3p | 6.683255906 | GBP1 | -2.7724655 |
| ssc-miR-143-3p | 6.683255906 | SRSF4 | -2.2871032 |
| ssc-miR-143-3p | 6.683255906 | P4HA1 | -2.01521 |
| ssc-miR-143-3p | 6.683255906 | EGLN1 | -2.59147 |
| ssc-miR-143-3p | 6.683255906 | CD40 | -2.6959412 |
| ssc-miR-143-3p | 6.683255906 | OVGP1 | -3.0902452 |
| ssc-miR-143-3p | 6.683255906 | NOLC1 | -2.3587146 |
| ssc-miR-143-3p | 6.683255906 | SAMHD1 | -6.521527 |
| ssc-miR-143-3p | 6.683255906 | CSN1S1 | -2.205338 |
| ssc-miR-143-3p | 6.683255906 | HPS1 | -2.3365586 |
| ssc-miR-143-3p | 6.683255906 | ATAD1 | -5.1717625 |
| ssc-miR-143-3p | 6.683255906 | IL2RG | -5.07989 |
| ssc-miR-143-3p | 6.683255906 | TLR6 | -2.8793592 |
| ssc-miR-143-3p | 6.683255906 | NFATC2 | -3.1316254 |
| ssc-miR-143-3p | 6.683255906 | PTPN1 | -2.4280972 |
| ssc-miR-143-3p | 6.683255906 | TRA2A | -2.8836782 |
| ssc-miR-143-3p | 6.683255906 | LEF1 | -14.021283 |
| ssc-miR-143-3p | 6.683255906 | PARP1 | -3.1783977 |
| ssc-miR-143-3p | 6.683255906 | BAG3 | -2.502677 |
| ssc-miR-143-3p | 6.683255906 | GATM | -2.5450995 |
| ssc-miR-143-3p | 6.683255906 | HIGD2A | -2.3788168 |
| ssc-miR-143-3p | 6.683255906 | RGS5 | -3.0175507 |
| ssc-miR-143-3p | 6.683255906 | FZR1 | -2.3384545 |
| ssc-miR-143-3p | 6.683255906 | NFATC1 | -3.4223328 |
| ssc-miR-143-3p | 6.683255906 | NFATC1 | -2.4886394 |
| ssc-miR-143-3p | 6.683255906 | MYCBP2 | -4.512695 |
| ssc-miR-143-3p | 6.683255906 | ASF1B | -3.822117 |
| ssc-miR-143-3p | 6.683255906 | NCOA3 | -3.8042808 |
| ssc-miR-143-3p | 6.683255906 | FLOT1 | -2.164263 |
| ssc-miR-143-3p | 6.683255906 | CTLA4 | -7.234559 |
| ssc-miR-143-3p | 6.683255906 | SEMA4F | -2.224211 |
| ssc-miR-143-3p | 6.683255906 | NCOA3 | -5.682131 |
| ssc-miR-143-3p | 6.683255906 | SLC23A2 | -2.8116944 |
| ssc-miR-143-3p | 6.683255906 | LEF1 | -13.521802 |
| ssc-miR-143-3p | 6.683255906 | ZNF558 | -4.5515246 |
| ssc-miR-143-3p | 6.683255906 | GABBR1 | -3.0064876 |
| ssc-miR-143-3p | 6.683255906 | HPSE | -5.4667473 |
| ssc-miR-143-3p | 6.683255906 | SHOC2 | -2.2087739 |
| ssc-miR-143-3p | 6.683255906 | FZR1 | -2.4563067 |
| ssc-miR-143-3p | 6.683255906 | MMD | -3.0891557 |
| ssc-miR-143-3p | 6.683255906 | COPZ1 | -2.0277896 |
| ssc-miR-143-3p | 6.683255906 | CD40 | -2.098875 |
| ssc-miR-143-3p | 6.683255906 | SAMHD1 | -4.093459 |
| ssc-miR-143-3p | 6.683255906 | HMGB1 | -4.439967 |
| ssc-miR-143-3p | 6.683255906 | WBP11 | -2.375831 |
| ssc-miR-143-3p | 6.683255906 | ATP5L | -3.3654962 |
| ssc-miR-143-3p | 6.683255906 | KIAA1967 | -3.913595 |
| ssc-miR-143-3p | 6.683255906 | MU | -2.0744867 |
| ssc-miR-143-3p | 6.683255906 | PPARD | -3.426806 |
| ssc-miR-143-3p | 6.683255906 | ORAI1 | -3.7655594 |
| ssc-miR-143-3p | 6.683255906 | RGS5 | -11.913826 |
| ssc-miR-143-3p | 6.683255906 | DDX58 | -4.4436197 |
| ssc-miR-143-3p | 6.683255906 | HMGB1 | -2.812081 |
| ssc-miR-143-3p | 6.683255906 | TXNRD1 | -2.0018742 |
|  |  |  |  |
| ssc-miR-30a-5p | 3.989309101 | IL12B | -4.19737 |
| ssc-miR-30a-5p | 3.989309101 | CASP3 | -2.3864603 |
| ssc-miR-30a-5p | 3.989309101 | CXCL9 | -3.9283998 |
| ssc-miR-30a-5p | 3.989309101 | DBF4 | -3.8504508 |
| ssc-miR-30a-5p | 3.989309101 | CASP3 | -2.3864603 |
| ssc-miR-30a-5p | 3.989309101 | RBMX | -2.3808525 |
| ssc-miR-30a-5p | 3.989309101 | MST4 | -5.8940544 |
| ssc-miR-30a-5p | 3.989309101 | PAN3 | -4.021991 |
| ssc-miR-30a-5p | 3.989309101 | GATM | -2.5589132 |
| ssc-miR-30a-5p | 3.989309101 | SMNDC1 | -2.052249 |
| ssc-miR-30a-5p | 3.989309101 | NFYB | -2.2796273 |
| ssc-miR-30a-5p | 3.989309101 | ITGA4 | -2.4564707 |
| ssc-miR-30a-5p | 3.989309101 | GATM | -2.213037 |
| ssc-miR-30a-5p | 3.989309101 | PBK | -2.210211 |
| ssc-miR-30a-5p | 3.989309101 | CD2 | -6.0158634 |
| ssc-miR-30a-5p | 3.989309101 | IL12B | -4.19737 |
| ssc-miR-30a-5p | 3.989309101 | TNIP1 | -3.2051425 |
| ssc-miR-30a-5p | 3.989309101 | SLAMF6 | -9.264798 |
| ssc-miR-30a-5p | 3.989309101 | IDE | -2.164203 |
| ssc-miR-30a-5p | 3.989309101 | NOL9 | -2.7997262 |
| ssc-miR-30a-5p | 3.989309101 | AHCY | -2.1658123 |
| ssc-miR-30a-5p | 3.989309101 | ARPC5 | -2.439057 |
| ssc-miR-30a-5p | 3.989309101 | PLAT | -2.1457462 |
| ssc-miR-30a-5p | 3.989309101 | NCK1 | -2.0576825 |
| ssc-miR-30a-5p | 3.989309101 | GATM | -2.3272405 |
| ssc-miR-30a-5p | 3.989309101 | NCK1 | -7.2229953 |
| ssc-miR-30a-5p | 3.989309101 | FOXO1 | -4.3853016 |
| ssc-miR-30a-5p | 3.989309101 | AHCY | -3.3859732 |
| ssc-miR-30a-5p | 3.989309101 | MMD | -5.547919 |
| ssc-miR-30a-5p | 3.989309101 | PNN | -2.1302779 |
| ssc-miR-30a-5p | 3.989309101 | LIPA | -2.1770744 |
| ssc-miR-30a-5p | 3.989309101 | GATM | -2.8943021 |
| ssc-miR-30a-5p | 3.989309101 | KLF13 | -2.8979645 |
| ssc-miR-30a-5p | 3.989309101 | YWHAZ | -2.032778 |
| ssc-miR-30a-5p | 3.989309101 | MDH2 | -2.7435343 |
| ssc-miR-30a-5p | 3.989309101 | P4HA1 | -2.01521 |
| ssc-miR-30a-5p | 3.989309101 | VCP | -2.6963546 |
| ssc-miR-30a-5p | 3.989309101 | ST8SIA4 | -2.2444808 |
| ssc-miR-30a-5p | 3.989309101 | IVD | -2.7258673 |
| ssc-miR-30a-5p | 3.989309101 | RPL32 | -2.4243426 |
| ssc-miR-30a-5p | 3.989309101 | CDC42 | -2.1404748 |
| ssc-miR-30a-5p | 3.989309101 | ARL6IP5 | -2.8254914 |
| ssc-miR-30a-5p | 3.989309101 | SERPINE1 | -2.6649761 |
| ssc-miR-30a-5p | 3.989309101 | ICOS | -10.873959 |
| ssc-miR-30a-5p | 3.989309101 | CXCL9 | -3.9283998 |
| ssc-miR-30a-5p | 3.989309101 | PTPN1 | -2.4280972 |
| ssc-miR-30a-5p | 3.989309101 | TRA2A | -2.8836782 |
| ssc-miR-30a-5p | 3.989309101 | SH2B3 | -2.0666575 |
| ssc-miR-30a-5p | 3.989309101 | GLS | -3.1439273 |
| ssc-miR-30a-5p | 3.989309101 | XPO7 | -2.1687493 |
| ssc-miR-30a-5p | 3.989309101 | VCP | -2.123445 |
| ssc-miR-30a-5p | 3.989309101 | DIABLO | -2.8235626 |
| ssc-miR-30a-5p | 3.989309101 | YWHAZ | -2.8391814 |
| ssc-miR-30a-5p | 3.989309101 | MTF2 | -3.5220673 |
| ssc-miR-30a-5p | 3.989309101 | PITPNB | -4.877218 |
| ssc-miR-30a-5p | 3.989309101 | GATM | -2.5450995 |
| ssc-miR-30a-5p | 3.989309101 | ICOS | -4.3020616 |
| ssc-miR-30a-5p | 3.989309101 | ARHGAP30 | -2.1464188 |
| ssc-miR-30a-5p | 3.989309101 | MDH2 | -2.2445135 |
| ssc-miR-30a-5p | 3.989309101 | PHF6 | -2.1167936 |
| ssc-miR-30a-5p | 3.989309101 | ARID4A | -2.4397392 |
| ssc-miR-30a-5p | 3.989309101 | ARL6IP5 | -2.9738982 |
| ssc-miR-30a-5p | 3.989309101 | YWHAZ | -2.0698745 |
| ssc-miR-30a-5p | 3.989309101 | LY75 | -3.0788105 |
| ssc-miR-30a-5p | 3.989309101 | ITPK1 | -2.6786532 |
| ssc-miR-30a-5p | 3.989309101 | CIT | -3.5340838 |
| ssc-miR-30a-5p | 3.989309101 | CD2 | -5.586634 |
| ssc-miR-30a-5p | 3.989309101 | XIAP | -2.8055277 |
| ssc-miR-30a-5p | 3.989309101 | PIKFYVE | -2.862979 |
| ssc-miR-30a-5p | 3.989309101 | MST4 | -7.942066 |
| ssc-miR-30a-5p | 3.989309101 | IVD | -4.4385552 |
| ssc-miR-30a-5p | 3.989309101 | RAI14 | -2.5591626 |
| ssc-miR-30a-5p | 3.989309101 | MDH2 | -3.0755162 |
| ssc-miR-30a-5p | 3.989309101 | UBE2J1 | -2.7590678 |
| ssc-miR-30a-5p | 3.989309101 | PLAT | -2.096734 |
| ssc-miR-30a-5p | 3.989309101 | BCLAF1 | -2.4743946 |
| ssc-miR-30a-5p | 3.989309101 | ZPBP2 | -2.4576108 |
| ssc-miR-30a-5p | 3.989309101 | SHOC2 | -2.2087739 |
| ssc-miR-30a-5p | 3.989309101 | MMD | -3.0891557 |
| ssc-miR-30a-5p | 3.989309101 | XIAP | -2.120111 |
| ssc-miR-30a-5p | 3.989309101 | BCL9 | BCL9 |
| ssc-miR-30a-5p | 3.989309101 | PAN3 | -2.9342384 |
| ssc-miR-30a-5p | 3.989309101 | FOXO1 | -3.7203667 |
| ssc-miR-30a-5p | 3.989309101 | BECN1 | -2.0962906 |
| ssc-miR-30a-5p | 3.989309101 | CD8A | -2.7839508 |
| ssc-miR-30a-5p | 3.989309101 | DDX58 | -4.4436197 |
| ssc-miR-30a-5p | 3.989309101 | GNAI2 | -3.229197 |
| ssc-miR-30a-5p | 3.989309101 | CD8A | -5.032429 |
| ssc-miR-30a-5p | 3.989309101 | FOXO1 | -4.668094 |
| ssc-miR-30a-5p | 3.989309101 | GYS2 | -8.879189 |
| ssc-miR-30a-5p | 3.989309101 | ST8SIA4 | -2.1632597 |
| ssc-miR-30a-5p | 3.989309101 | RPL32 | -4.7264094 |
| ssc-miR-30a-5p | 3.989309101 | CASP3 | -2.2549624 |
| ssc-miR-30a-5p | 3.989309101 | ARID4A | -3.5558572 |
|  |  |  |  |
| ssc-miR-novel-chr2_21624 | 6.683255906 | JAK3 | -2.6891918 |
| ssc-miR-novel-chr2_21624 | 6.683255906 | CSN2 | -2.4015813 |
| ssc-miR-novel-chr2_21624 | 6.683255906 | FZR1 | -2.0416865 |
| ssc-miR-novel-chr2_21624 | 6.683255906 | NOC3L | -3.3583956 |
| ssc-miR-novel-chr2_21624 | 6.683255906 | SLA-DQA | -2.6939797 |
| ssc-miR-novel-chr2_21624 | 6.683255906 | NCOA3 | -2.3024986 |
| ssc-miR-novel-chr2_21624 | 6.683255906 | HR | -2.116809 |
| ssc-miR-novel-chr2_21624 | 6.683255906 | TXNRD1 | -2.9226952 |
| ssc-miR-novel-chr2_21624 | 6.683255906 | POLR2B | -2.5943034 |
| ssc-miR-novel-chr2_21624 | 6.683255906 | IL2RG | -4.2400565 |
| ssc-miR-novel-chr2_21624 | 6.683255906 | ENTPD1 | -2.0566626 |
| ssc-miR-novel-chr2_21624 | 6.683255906 | GATM | -2.5589132 |
| ssc-miR-novel-chr2_21624 | 6.683255906 | POLR2B | -2.452575 |
| ssc-miR-novel-chr2_21624 | 6.683255906 | USP47 | -3.5484018 |
| ssc-miR-novel-chr2_21624 | 6.683255906 | ATF7IP | -2.1011086 |
| ssc-miR-novel-chr2_21624 | 6.683255906 | ITGA4 | -2.4564707 |
| ssc-miR-novel-chr2_21624 | 6.683255906 | GATM | -2.213037 |
| ssc-miR-novel-chr2_21624 | 6.683255906 | CASQ1 | -4.4726872 |
| ssc-miR-novel-chr2_21624 | 6.683255906 | PBK | -2.210211 |
| ssc-miR-novel-chr2_21624 | 6.683255906 | NFATC2 | -2.687189 |
| ssc-miR-novel-chr2_21624 | 6.683255906 | DDX52 | -3.784038 |
| ssc-miR-novel-chr2_21624 | 6.683255906 | CAPRIN1 | -2.016781 |
| ssc-miR-novel-chr2_21624 | 6.683255906 | MAP3K7 | -3.955688 |
| ssc-miR-novel-chr2_21624 | 6.683255906 | CD19 | -8.638682 |
| ssc-miR-novel-chr2_21624 | 6.683255906 | MMP11 | -2.1907008 |
| ssc-miR-novel-chr2_21624 | 6.683255906 | IKBKB | -2.1580052 |
| ssc-miR-novel-chr2_21624 | 6.683255906 | GATM | -2.3272405 |
| ssc-miR-novel-chr2_21624 | 6.683255906 | MMD | -5.547919 |
| ssc-miR-novel-chr2_21624 | 6.683255906 | CD40 | -4.9732995 |
| ssc-miR-novel-chr2_21624 | 6.683255906 | GATM | -2.8943021 |
| ssc-miR-novel-chr2_21624 | 6.683255906 | CTSB | -2.3818676 |
| ssc-miR-novel-chr2_21624 | 6.683255906 | SLA-DOB | -11.237711 |
| ssc-miR-novel-chr2_21624 | 6.683255906 | CDK4 |  |
| ssc-miR-novel-chr2_21624 | 6.683255906 | CDK4 | -2.4161417 |
| ssc-miR-novel-chr2_21624 | 6.683255906 | KLF13 | -2.8979645 |
| ssc-miR-novel-chr2_21624 | 6.683255906 | CDK4 | -2.2926328 |
| ssc-miR-novel-chr2_21624 | 6.683255906 | PPARD | -2.3047228 |
| ssc-miR-novel-chr2_21624 | 6.683255906 | GBP1 | -2.7724655 |
| ssc-miR-novel-chr2_21624 | 6.683255906 | SRSF4 | -2.2871032 |
| ssc-miR-novel-chr2_21624 | 6.683255906 | P4HA1 | -2.01521 |
| ssc-miR-novel-chr2_21624 | 6.683255906 | EGLN1 | -2.59147 |
| ssc-miR-novel-chr2_21624 | 6.683255906 | CD40 | -2.6959412 |
| ssc-miR-novel-chr2_21624 | 6.683255906 | OVGP1 | -3.0902452 |
| ssc-miR-novel-chr2_21624 | 6.683255906 | NOLC1 | -2.3587146 |
| ssc-miR-novel-chr2_21624 | 6.683255906 | SAMHD1 | -6.521527 |
| ssc-miR-novel-chr2_21624 | 6.683255906 | CSN1S1 | -2.205338 |
| ssc-miR-novel-chr2_21624 | 6.683255906 | HPS1 | -2.3365586 |
| ssc-miR-novel-chr2_21624 | 6.683255906 | ATAD1 | -5.1717625 |
| ssc-miR-novel-chr2_21624 | 6.683255906 | IL2RG | -5.07989 |
| ssc-miR-novel-chr2_21624 | 6.683255906 | ICOS | -10.873959 |
| ssc-miR-novel-chr2_21624 | 6.683255906 | TLR6 | -2.8793592 |
| ssc-miR-novel-chr2_21624 | 6.683255906 | NFATC2 | -3.1316254 |
| ssc-miR-novel-chr2_21624 | 6.683255906 | PTPN1 | -2.4280972 |
| ssc-miR-novel-chr2_21624 | 6.683255906 | TRA2A | -2.8836782 |
| ssc-miR-novel-chr2_21624 | 6.683255906 | LEF1 | -14.021283 |
| ssc-miR-novel-chr2_21624 | 6.683255906 | PARP1 | -3.1783977 |
| ssc-miR-novel-chr2_21624 | 6.683255906 | BAG3 | -2.502677 |
| ssc-miR-novel-chr2_21624 | 6.683255906 | GATM | -2.5450995 |
| ssc-miR-novel-chr2_21624 | 6.683255906 | HIGD2A | -2.3788168 |
| ssc-miR-novel-chr2_21624 | 6.683255906 | RGS5 | -3.0175507 |
| ssc-miR-novel-chr2_21624 | 6.683255906 | FZR1 | -2.3384545 |
| ssc-miR-novel-chr2_21624 | 6.683255906 | NFATC1 | -3.4223328 |
| ssc-miR-novel-chr2_21624 | 6.683255906 | NFATC1 | -2.4886394 |
| ssc-miR-novel-chr2_21624 | 6.683255906 | MYCBP2 | -4.512695 |
| ssc-miR-novel-chr2_21624 | 6.683255906 | ASF1B | -3.822117 |
| ssc-miR-novel-chr2_21624 | 6.683255906 | NCOA3 | -3.8042808 |
| ssc-miR-novel-chr2_21624 | 6.683255906 | FLOT1 | -2.164263 |
| ssc-miR-novel-chr2_21624 | 6.683255906 | CTLA4 | -7.234559 |
| ssc-miR-novel-chr2_21624 | 6.683255906 | SEMA4F | -2.224211 |
| ssc-miR-novel-chr2_21624 | 6.683255906 | NCOA3 | -5.682131 |
| ssc-miR-novel-chr2_21624 | 6.683255906 | SLC23A2 | -2.8116944 |
| ssc-miR-novel-chr2_21624 | 6.683255906 | LEF1 | -13.521802 |
| ssc-miR-novel-chr2_21624 | 6.683255906 | ZNF558 | -4.5515246 |
| ssc-miR-novel-chr2_21624 | 6.683255906 | GABBR1 | -3.0064876 |
| ssc-miR-novel-chr2_21624 | 6.683255906 | HPSE | -5.4667473 |
| ssc-miR-novel-chr2_21624 | 6.683255906 | SHOC2 | -2.2087739 |
| ssc-miR-novel-chr2_21624 | 6.683255906 | FZR1 | -2.4563067 |
| ssc-miR-novel-chr2_21624 | 6.683255906 | MMD | -3.0891557 |
| ssc-miR-novel-chr2_21624 | 6.683255906 | COPZ1 | -2.0277896 |
| ssc-miR-novel-chr2_21624 | 6.683255906 | CD40 | -2.098875 |
| ssc-miR-novel-chr2_21624 | 6.683255906 | SAMHD1 | -4.093459 |
| ssc-miR-novel-chr2_21624 | 6.683255906 | HMGB1 | -4.439967 |
| ssc-miR-novel-chr2_21624 | 6.683255906 | WBP11 | -2.375831 |
| ssc-miR-novel-chr2_21624 | 6.683255906 | ATP5L | -3.3654962 |
| ssc-miR-novel-chr2_21624 | 6.683255906 | KIAA1967 | -3.913595 |
| ssc-miR-novel-chr2_21624 | 6.683255906 | MU | -2.0744867 |
| ssc-miR-novel-chr2_21624 | 6.683255906 | PPARD | -3.426806 |
| ssc-miR-novel-chr2_21624 | 6.683255906 | ORAI1 | -3.7655594 |
| ssc-miR-novel-chr2_21624 | 6.683255906 | RGS5 | -11.913826 |
| ssc-miR-novel-chr2_21624 | 6.683255906 | DDX58 | -4.4436197 |
| ssc-miR-novel-chr2_21624 | 6.683255906 | HMGB1 | -2.812081 |
| ssc-miR-novel-chr2_21624 | 6.683255906 | TXNRD1 | -2.0018742 |
|  |  |  |  |
| ssc-let-7g | 0.382434302 | ATP1B1 | 3.8346922 |
| ssc-let-7g | 0.382434302 | HOPX | 4.075246 |
| ssc-let-7g | 0.382434302 | MGLL | 2.009778 |
| ssc-let-7g | 0.382434302 | DDIT3 | 2.2071683 |
| ssc-let-7g | 0.382434302 | ANGPTL2 | 4.0596805 |
| ssc-let-7g | 0.382434302 | FABP5 | 3.1219811 |
| ssc-let-7g | 0.382434302 | SCD | 3.746366 |
| ssc-let-7g | 0.382434302 | RIMS1 | 2.6629915 |
| ssc-let-7g | 0.382434302 | CD34 | 3.0409834 |
| ssc-let-7g | 0.382434302 | RNASE4 | 4.3675966 |
| ssc-let-7g | 0.382434302 | MMP14 | 2.1474407 |
| ssc-let-7g | 0.382434302 | DUOX2 | 71.88512 |
| ssc-let-7g | 0.382434302 | BMPR2 | 3.1323707 |
| ssc-let-7g | 0.382434302 | RNASE4 | 5.184753 |
| ssc-let-7g | 0.382434302 | KCNE3 | 2.5129766 |
| ssc-let-7g | 0.382434302 | DKK3 | 5.687311 |
| ssc-let-7g | 0.382434302 | SCD | 5.3195086 |
| ssc-let-7g | 0.382434302 | ITGAV | 2.1254475 |
| ssc-let-7g | 0.382434302 | ITGAV | 2.0350585 |
| ssc-let-7g | 0.382434302 | CES3 | 17.450926 |
| ssc-let-7g | 0.382434302 | CES3 | 4.478221 |
| ssc-let-7g | 0.382434302 | MBP | 3.3519206 |
| ssc-let-7g | 0.382434302 | CD34 | 4.1547093 |
| ssc-let-7g | 0.382434302 | KLF9 | 3.7045405 |
| ssc-let-7g | 0.382434302 | SCD | 3.9770987 |
| ssc-let-7g | 0.382434302 | LAMP2 | 2.1236007 |
| ssc-let-7g | 0.382434302 | ZNF268 | 2.5049593 |
| ssc-let-7g | 0.382434302 | WNT2B | PREDICTED: Sus scrofa wingless-type MMTV integration site family, member 2B (WNT2B), mRNA [XM_003125849] |
| ssc-let-7g | 0.382434302 | ANGPTL2 | 3.7980468 |
| ssc-let-7g | 0.382434302 | KLF9 | 5.3736143 |
| ssc-let-7g | 0.382434302 | SEC14L2 | 4.428872 |
| ssc-let-7g | 0.382434302 | KLF9 | 4.947474 |
| ssc-let-7g | 0.382434302 | EPAS1 | 3.7910812 |
| ssc-let-7g | 0.382434302 | RAB22A | 2.8142457 |
| ssc-let-7g | 0.382434302 | CHSY1 | 6.469561 |
| ssc-let-7g | 0.382434302 | FABP5 | 2.712679 |
| ssc-let-7g | 0.382434302 | DMD | 6.0926504 |
| ssc-let-7g | 0.382434302 | DNAJA4 | 6.9199743 |
| ssc-let-7g | 0.382434302 | KLF9 | 5.9025326 |
| ssc-let-7g | 0.382434302 | WWTR1 | 2.9029868 |
| ssc-let-7g | 0.382434302 | KCNE3 | 2.4654815 |
| ssc-let-7g | 0.382434302 | APP | 2.1256132 |
| ssc-let-7g | 0.382434302 | EPAS1 | 4.391089 |
| ssc-let-7g | 0.382434302 | S100A14 | 8.052408 |
| ssc-let-7g | 0.382434302 | KLF9 | 4.886205 |
| ssc-let-7g | 0.382434302 | SGCD | 2.3677456 |
| ssc-let-7g | 0.382434302 | SNX16 | 2.076848 |
| ssc-let-7g | 0.382434302 | SEC14L2 | 3.2673485 |
| ssc-let-7g | 0.382434302 | MBP | 2.3703585 |
| ssc-let-7g | 0.382434302 | PKIA | 2.6770973 |
| ssc-let-7g | 0.382434302 | APP | 3.000465 |
| ssc-let-7g | 0.382434302 | KLF9 | 4.58542 |
| ssc-let-7g | 0.382434302 | FABP5 | 2.5523407 |
| ssc-let-7g | 0.382434302 | SCD | 3.4657626 |
| ssc-let-7g | 0.382434302 | MYO6 | 4.3761973 |
| ssc-let-7g | 0.382434302 | TNFRSF1B | 4.2128057 |
| ssc-let-7g | 0.382434302 | FABP5 | 4.1442533 |
| ssc-let-7g | 0.382434302 | APP | 2.6229365 |
| ssc-let-7g | 0.382434302 | STC1 | 3.2388 |
| ssc-let-7g | 0.382434302 | NR3C2 | 3.0180695 |
| ssc-let-7g | 0.382434302 | B3GNT5 | 2.965213 |
| ssc-let-7g | 0.382434302 | PTPRU | 2.205255 |
| ssc-let-7g | 0.382434302 | GFPT1 | 3.0540628 |
| ssc-let-7g | 0.382434302 | MAGI3 | 2.390861 |
| ssc-let-7g | 0.382434302 | KRT5 | 2.6214848 |
| ssc-let-7g | 0.382434302 | DKK3 | 6.8756404 |
| ssc-let-7g | 0.382434302 | PKIA | 2.2394006 |
|  |  |  |  |
| ssc-miR-10b | 0.490946368 | PIK3IP1 | 2.3679569 |
| ssc-miR-10b | 0.490946368 | IL15 | 2.3639562 |
| ssc-miR-10b | 0.490946368 | PRDX6 | 2.369106 |
| ssc-miR-10b | 0.490946368 | CD9 | 13.268091 |
| ssc-miR-10b | 0.490946368 | TCN1 | 8.882277 |
| ssc-miR-10b | 0.490946368 | SCD5 | 3.2284265 |
| ssc-miR-10b | 0.490946368 | TEAD4 | 2.688287 |
| ssc-miR-10b | 0.490946368 | RIMS1 | 2.6629915 |
| ssc-miR-10b | 0.490946368 | CD34 | 3.0409834 |
| ssc-miR-10b | 0.490946368 | MMP14 | 2.1474407 |
| ssc-miR-10b | 0.490946368 | DUOX2 | 71.88512 |
| ssc-miR-10b | 0.490946368 | ID4 | 4.893096 |
| ssc-miR-10b | 0.490946368 | PRDX6 | 3.8394175 |
| ssc-miR-10b | 0.490946368 | ADAMTS1 | 14.238098 |
| ssc-miR-10b | 0.490946368 | KCNE3 | 2.5129766 |
| ssc-miR-10b | 0.490946368 | CD34 | 4.1547093 |
| ssc-miR-10b | 0.490946368 | WNT2B | PREDICTED: Sus scrofa wingless-type MMTV integration site family, member 2B (WNT2B), mRNA [XM_003125849] |
| ssc-miR-10b | 0.490946368 | NOR-1 | 3.7146902 |
| ssc-miR-10b | 0.490946368 | SEC14L2 | 4.428872 |
| ssc-miR-10b | 0.490946368 | ITM2A | 3.547098 |
| ssc-miR-10b | 0.490946368 | PRDX6 | 2.467787 |
| ssc-miR-10b | 0.490946368 | KCNE3 | 2.4654815 |
| ssc-miR-10b | 0.490946368 | MS4A2 | 2.0332665 |
| ssc-miR-10b | 0.490946368 | CD9 | 9.383954 |
| ssc-miR-10b | 0.490946368 | SEC14L2 | 3.2673485 |
| ssc-miR-10b | 0.490946368 | UGP2 | 3.809193 |
| ssc-miR-10b | 0.490946368 | VDAC1P5 | 2.6776247 |
| ssc-miR-10b | 0.490946368 | ALOX12 | 11.447397 |
| ssc-miR-10b | 0.490946368 | DES | 2.1293135 |
| ssc-miR-10b | 0.490946368 | CHD6 | 2.2985008 |
| ssc-miR-10b | 0.490946368 | CXCL2 | 16.739788 |
| ssc-miR-10b | 0.490946368 | EMP1 | 2.4735997 |
| ssc-miR-10b | 0.490946368 | KLHL3 | 2.542577 |
| ssc-miR-10b | 0.490946368 | DES | 2.3078039 |
| ssc-miR-10b | 0.490946368 | PRKAA2 | 19.163576 |
| ssc-miR-10b | 0.490946368 | CCR1 | 2.5625281 |
| ssc-miR-10b | 0.490946368 | CD9 | 8.2587595 |
| ssc-miR-10b | 0.490946368 | THBD | 41.88992 |
| ssc-miR-10b | 0.490946368 | CCR1 | 4.615539 |
| ssc-miR-10b | 0.490946368 | PRDX6 | 2.5033536 |
| ssc-miR-10b | 0.490946368 | CXCL2 | 11.182136 |
| ssc-miR-10b | 0.490946368 | CD9 | 13.421032 |
| ssc-miR-10b | 0.490946368 | CNN1 | 3.4723577 |
| ssc-miR-10b | 0.490946368 | NPTN | 2.735852 |
| ssc-miR-10b | 0.490946368 | THBD | 8.153293 |
| ssc-miR-10b | 0.490946368 | CD9 | 13.268091 |
|  |  |  |  |
| ssc-miR-155-5p | 0.009600252 | PKIA | 2.310977 |
| ssc-miR-155-5p | 0.009600252 | CLIP1 | 2.1740522 |
| ssc-miR-155-5p | 0.009600252 | FMO1 | 115.05307 |
| ssc-miR-155-5p | 0.009600252 | PDK4 | 7.5410895 |
| ssc-miR-155-5p | 0.009600252 | ME1 | 2.6170402 |
| ssc-miR-155-5p | 0.009600252 | CLEC7A | 2.8455265 |
| ssc-miR-155-5p | 0.009600252 | SIX1 | 2.3128672 |
| ssc-miR-155-5p | 0.009600252 | ATP1B1 | 5.6342463 |
| ssc-miR-155-5p | 0.009600252 | FAM177A1 | 2.7517347 |
| ssc-miR-155-5p | 0.009600252 | PDK4 | 7.589423 |
| ssc-miR-155-5p | 0.009600252 | CFL2 | 2.8280158 |
| ssc-miR-155-5p | 0.009600252 | PLN | 5.4343724 |
| ssc-miR-155-5p | 0.009600252 | CTSL1 | 3.4175367 |
| ssc-miR-155-5p | 0.009600252 | SCD5 | 3.2284265 |
| ssc-miR-155-5p | 0.009600252 | CLEC7A | 8.99005 |
| ssc-miR-155-5p | 0.009600252 | KLF10 | 2.6501894 |
| ssc-miR-155-5p | 0.009600252 | RIMS1 | 2.6629915 |
| ssc-miR-155-5p | 0.009600252 | PRNP | 2.4658751 |
| ssc-miR-155-5p | 0.009600252 | ANXA1 | 11.260019 |
| ssc-miR-155-5p | 0.009600252 | BMPR2 | 3.1323707 |
| ssc-miR-155-5p | 0.009600252 | FAM177A1 | 2.1352532 |
| ssc-miR-155-5p | 0.009600252 | DDO | 19.63778 |
| ssc-miR-155-5p | 0.009600252 | RDX | 2.7281008 |
| ssc-miR-155-5p | 0.009600252 | OXR1 | 4.7151937 |
| ssc-miR-155-5p | 0.009600252 | CTSL1 | 2.6535535 |
| ssc-miR-155-5p | 0.009600252 | INPP5F | 2.317872 |
| ssc-miR-155-5p | 0.009600252 | RDX | 2.2962925 |
| ssc-miR-155-5p | 0.009600252 | PLN | 4.9842076 |
| ssc-miR-155-5p | 0.009600252 | CTSL1 | 2.5077343 |
| ssc-miR-155-5p | 0.009600252 | PRNP | 3.892647 |
| ssc-miR-155-5p | 0.009600252 | ANXA1 | 9.256069 |
| ssc-miR-155-5p | 0.009600252 | LAMP2 | 2.1236007 |
| ssc-miR-155-5p | 0.009600252 | ZNF268 | 2.5049593 |
| ssc-miR-155-5p | 0.009600252 | PDK4 | 4.2704554 |
| ssc-miR-155-5p | 0.009600252 | NPC2 | 3.755565 |
| ssc-miR-155-5p | 0.009600252 | KLF10 | 2.2958229 |
| ssc-miR-155-5p | 0.009600252 | CAV2 | 31.894093 |
| ssc-miR-155-5p | 0.009600252 | EPAS1 | 3.7910812 |
| ssc-miR-155-5p | 0.009600252 | MS4A2 | 2.0332665 |
| ssc-miR-155-5p | 0.009600252 | EPAS1 | 4.391089 |
| ssc-miR-155-5p | 0.009600252 | PLN | 4.8746552 |
| ssc-miR-155-5p | 0.009600252 | ATP1B1 | 5.595114 |
| ssc-miR-155-5p | 0.009600252 | TNFAIP6 | 2.8435597 |
| ssc-miR-155-5p | 0.009600252 | FMO1 | 16.316187 |
| ssc-miR-155-5p | 0.009600252 | PRNP | 3.8958378 |
| ssc-miR-155-5p | 0.009600252 | PKIA | 2.6770973 |
| ssc-miR-155-5p | 0.009600252 | CRYAB | 5.640287 |
| ssc-miR-155-5p | 0.009600252 | KIT | 2.660936 |
| ssc-miR-155-5p | 0.009600252 | CDO1 | 4.3074837 |
| ssc-miR-155-5p | 0.009600252 | CAV2 | 14.56954 |
| ssc-miR-155-5p | 0.009600252 | ANXA1 | 8.000692 |
| ssc-miR-155-5p | 0.009600252 | CFL2 | 2.513711 |
| ssc-miR-155-5p | 0.009600252 | SOAT1 | 3.0981987 |
| ssc-miR-155-5p | 0.009600252 | CTSL1 | 5.6874614 |
| ssc-miR-155-5p | 0.009600252 | CAT | 2.4278195 |
| ssc-miR-155-5p | 0.009600252 | BTG3 | 5.4815583 |
| ssc-miR-155-5p | 0.009600252 | BTG3 | 3.125916 |
| ssc-miR-155-5p | 0.009600252 | CHD6 | 2.2985008 |
| ssc-miR-155-5p | 0.009600252 | ATP1B1 | 4.1853924 |
| ssc-miR-155-5p | 0.009600252 | ALG10 | 2.121506 |
| ssc-miR-155-5p | 0.009600252 | ST3GAL4 | 3.8444028 |
| ssc-miR-155-5p | 0.009600252 | CLIP1 | 4.0972705 |
| ssc-miR-155-5p | 0.009600252 | CFL2 | 2.3713303 |
| ssc-miR-155-5p | 0.009600252 | ANXA1 | 31.112946 |
| ssc-miR-155-5p | 0.009600252 | PRNP | 4.534527 |
| ssc-miR-155-5p | 0.009600252 | CXCL2 | 16.739788 |
| ssc-miR-155-5p | 0.009600252 | CAT | 2.6168854 |
| ssc-miR-155-5p | 0.009600252 | EMP1 | 2.4735997 |
| ssc-miR-155-5p | 0.009600252 | PDK4 | 2.356212 |
| ssc-miR-155-5p | 0.009600252 | CAT | 2.3428736 |
| ssc-miR-155-5p | 0.009600252 | ANKRD22 | 2.9717975 |
| ssc-miR-155-5p | 0.009600252 | PDK4 | 5.8856487 |
| ssc-miR-155-5p | 0.009600252 | TNFAIP6 | 8.756827 |
| ssc-miR-155-5p | 0.009600252 | ARGLU1 | 2.3496652 |
| ssc-miR-155-5p | 0.009600252 | PDK4 | 4.9515734 |
| ssc-miR-155-5p | 0.009600252 | CAV2 | 18.59619 |
| ssc-miR-155-5p | 0.009600252 | BTG3 | 3.769016 |
| ssc-miR-155-5p | 0.009600252 | CXCL2 | 11.182136 |
| ssc-miR-155-5p | 0.009600252 | PKIA | 2.2394006 |
| ssc-miR-155-5p | 0.009600252 | FOS | 2.1044629 |
| ssc-miR-155-5p | 0.009600252 | CTSL1 | 3.3290515 |
| ssc-miR-155-5p | 0.009600252 | OLR1 | 3.0760937 |
| ssc-miR-155-5p | 0.009600252 | SIX1 | 3.7485037 |
| ssc-miR-155-5p | 0.009600252 | ST3GAL4 | 3.185859 |
|  |  |  |  |
| ssc-miR-16 | 0.160286728 | ATP9A | 2.904007 |
| ssc-miR-16 | 0.160286728 | BTG2 | 2.218317 |
| ssc-miR-16 | 0.160286728 | TUFT1 | 3.118481 |
| ssc-miR-16 | 0.160286728 | SPARCL1 | 10.201537 |
| ssc-miR-16 | 0.160286728 | STEAP4 | 2.5378268 |
| ssc-miR-16 | 0.160286728 | SPARCL1 | 9.67489 |
| ssc-miR-16 | 0.160286728 | PDK4 | 7.589423 |
| ssc-miR-16 | 0.160286728 | ANGPTL2 | 4.0596805 |
| ssc-miR-16 | 0.160286728 | ACTA1 | 5.594617 |
| ssc-miR-16 | 0.160286728 | SCD | 3.746366 |
| ssc-miR-16 | 0.160286728 | DUOX2 | 71.88512 |
| ssc-miR-16 | 0.160286728 | DDO | 19.63778 |
| ssc-miR-16 | 0.160286728 | HEY1 | 4.326854 |
| ssc-miR-16 | 0.160286728 | VCL | 4.3770857 |
| ssc-miR-16 | 0.160286728 | PRDX6 | 3.8394175 |
| ssc-miR-16 | 0.160286728 | DKK3 | 5.687311 |
| ssc-miR-16 | 0.160286728 | SCD | 5.3195086 |
| ssc-miR-16 | 0.160286728 | ITGAV | 2.1254475 |
| ssc-miR-16 | 0.160286728 | ITGAV | 2.0350585 |
| ssc-miR-16 | 0.160286728 | SOD2 | 2.1154256 |
| ssc-miR-16 | 0.160286728 | SCD | 3.9770987 |
| ssc-miR-16 | 0.160286728 | ACTA1 | 4.4613676 |
| ssc-miR-16 | 0.160286728 | C1QTNF3 | 4.5795827 |
| ssc-miR-16 | 0.160286728 | PDK4 | 4.2704554 |
| ssc-miR-16 | 0.160286728 | ANGPTL2 | 3.7980468 |
| ssc-miR-16 | 0.160286728 | SEC14L2 | 4.428872 |
| ssc-miR-16 | 0.160286728 | MEST | 7.9542184 |
| ssc-miR-16 | 0.160286728 | SPARCL1 | 10.548973 |
| ssc-miR-16 | 0.160286728 | NPC2 | 3.755565 |
| ssc-miR-16 | 0.160286728 | RAB22A | 2.8142457 |
| ssc-miR-16 | 0.160286728 | PRDX6 | 2.467787 |
| ssc-miR-16 | 0.160286728 | WWTR1 | 2.9029868 |
| ssc-miR-16 | 0.160286728 | MS4A2 | 2.0332665 |
| ssc-miR-16 | 0.160286728 | APP | 2.1256132 |
| ssc-miR-16 | 0.160286728 | VCL | 3.2573445 |
| ssc-miR-16 | 0.160286728 | SNX16 | 2.076848 |
| ssc-miR-16 | 0.160286728 | SEC14L2 | 3.2673485 |
| ssc-miR-16 | 0.160286728 | APP | 3.000465 |
| ssc-miR-16 | 0.160286728 | UGP2 | 3.809193 |
| ssc-miR-16 | 0.160286728 | FLRT3 | 17.998533 |
| ssc-miR-16 | 0.160286728 | SCD | 3.4657626 |
| ssc-miR-16 | 0.160286728 | MYO6 | 4.3761973 |
| ssc-miR-16 | 0.160286728 | RAB32 | 2.6038885 |
| ssc-miR-16 | 0.160286728 | GRB10 | 3.0650427 |
| ssc-miR-16 | 0.160286728 | VEGFA | 2.4972408 |
| ssc-miR-16 | 0.160286728 | SPARCL1 | 6.670773 |
| ssc-miR-16 | 0.160286728 | NEDD9 | 5.1763387 |
| ssc-miR-16 | 0.160286728 | SOAT1 | 3.0981987 |
| ssc-miR-16 | 0.160286728 | APP | 2.6229365 |
| ssc-miR-16 | 0.160286728 | ALOX12 | 11.447397 |
| ssc-miR-16 | 0.160286728 | CHD6 | 2.2985008 |
| ssc-miR-16 | 0.160286728 | VCL | 2.3374903 |
| ssc-miR-16 | 0.160286728 | MBTPS1 | 2.051728 |
| ssc-miR-16 | 0.160286728 | MAGI3 | 2.390861 |
| ssc-miR-16 | 0.160286728 | SPARCL1 | 15.350687 |
| ssc-miR-16 | 0.160286728 | PDK4 | 2.356212 |
| ssc-miR-16 | 0.160286728 | PDK4 | 5.8856487 |
| ssc-miR-16 | 0.160286728 | ARGLU1 | 2.3496652 |
| ssc-miR-16 | 0.160286728 | PDK4 | 4.9515734 |
| ssc-miR-16 | 0.160286728 | PRDX6 | 2.5033536 |
| ssc-miR-16 | 0.160286728 | TXNIP | 2.4701765 |
| ssc-miR-16 | 0.160286728 | CACNA2D1 | 2.353123 |
| ssc-miR-16 | 0.160286728 | DKK3 | 6.8756404 |
| ssc-miR-16 | 0.160286728 | ACTA1 | 2.011736 |
| ssc-miR-16 | 0.160286728 | SLC9A3R2 | 5.7386975 |
| ssc-miR-16 | 0.160286728 | TXNIP | 2.3313346 |
| ssc-miR-16 | 0.160286728 | TXNIP | 2.7284145 |
| ssc-miR-16 | 0.160286728 | VEGFA | 2.0567462 |
| ssc-miR-16 | 0.160286728 | TXNIP | 2.7425334 |
| ssc-miR-16 | 0.160286728 | ARL3 | 2.4176466 |
| ssc-miR-16 | 0.160286728 | CNN1 | 3.4723577 |
| ssc-miR-16 | 0.160286728 | VCL | 2.7650511 |
| ssc-miR-16 | 0.160286728 | SIX1 | 3.7485037 |
| ssc-miR-16 | 0.160286728 | SPARCL1 | 10.201537 |
|  |  |  |  |
| ssc-miR-17-5p | 0.088178295 | NR4A2 | 2.8954253 |
| ssc-miR-17-5p | 0.088178295 | RBM8A | 2.0222304 |
| ssc-miR-17-5p | 0.088178295 | TBX3 | 25.165472 |
| ssc-miR-17-5p | 0.088178295 | SOAT1 | 2.1717749 |
| ssc-miR-17-5p | 0.088178295 | APP | 2.1658723 |
| ssc-miR-17-5p | 0.088178295 | ST3GAL4 | 3.185859 |
| ssc-miR-17-5p | 0.088178295 | EDNRB | 4.742645 |
| ssc-miR-17-5p | 0.088178295 | OXCT1 | 2.0291076 |
| ssc-miR-17-5p | 0.088178295 | CFL2 | 2.8280158 |
| ssc-miR-17-5p | 0.088178295 | OCLN | 6.9868617 |
| ssc-miR-17-5p | 0.088178295 | MEX3D | 2.4318736 |
| ssc-miR-17-5p | 0.088178295 | SCD5 | 3.2284265 |
| ssc-miR-17-5p | 0.088178295 | TEAD4 | 2.688287 |
| ssc-miR-17-5p | 0.088178295 | KLF10 | 2.6501894 |
| ssc-miR-17-5p | 0.088178295 | PRNP | 2.4658751 |
| ssc-miR-17-5p | 0.088178295 | CAPN2 | 11.183791 |
| ssc-miR-17-5p | 0.088178295 | BMPR2 | 3.1323707 |
| ssc-miR-17-5p | 0.088178295 | LYZ | 18.577944 |
| ssc-miR-17-5p | 0.088178295 | RDX | 2.7281008 |
| ssc-miR-17-5p | 0.088178295 | OXR1 | 4.7151937 |
| ssc-miR-17-5p | 0.088178295 | RAB11FIP1 | 5.0282693 |
| ssc-miR-17-5p | 0.088178295 | ADAMTS1 | 14.238098 |
| ssc-miR-17-5p | 0.088178295 | RDX | 2.2962925 |
| ssc-miR-17-5p | 0.088178295 | TBX3 | 21.275673 |
| ssc-miR-17-5p | 0.088178295 | GUCY2C | 2.614502 |
| ssc-miR-17-5p | 0.088178295 | KLF9 | 3.7045405 |
| ssc-miR-17-5p | 0.088178295 | RHOB | 2.540106 |
| ssc-miR-17-5p | 0.088178295 | PRNP | 3.892647 |
| ssc-miR-17-5p | 0.088178295 | LAMP2 | 2.1236007 |
| ssc-miR-17-5p | 0.088178295 | EDNRA | 3.8113816 |
| ssc-miR-17-5p | 0.088178295 | NOR-1 | 3.7146902 |
| ssc-miR-17-5p | 0.088178295 | KLF9 | 5.3736143 |
| ssc-miR-17-5p | 0.088178295 | LYZ | 11.652282 |
| ssc-miR-17-5p | 0.088178295 | OCLN | 30.06069 |
| ssc-miR-17-5p | 0.088178295 | KLF10 | 2.2958229 |
| ssc-miR-17-5p | 0.088178295 | OCLN | 30.517065 |
| ssc-miR-17-5p | 0.088178295 | KLF9 | 4.947474 |
| ssc-miR-17-5p | 0.088178295 | CAV2 | 31.894093 |
| ssc-miR-17-5p | 0.088178295 | EPAS1 | 3.7910812 |
| ssc-miR-17-5p | 0.088178295 | RAB22A | 2.8142457 |
| ssc-miR-17-5p | 0.088178295 | KLF6 | 2.2367525 |
| ssc-miR-17-5p | 0.088178295 | RHOB | 2.0275912 |
| ssc-miR-17-5p | 0.088178295 | KLF9 | 5.9025326 |
| ssc-miR-17-5p | 0.088178295 | FGL2 | 6.0749955 |
| ssc-miR-17-5p | 0.088178295 | APP | 2.1256132 |
| ssc-miR-17-5p | 0.088178295 | EPAS1 | 4.391089 |
| ssc-miR-17-5p | 0.088178295 | S100A14 | 8.052408 |
| ssc-miR-17-5p | 0.088178295 | KLF9 | 4.886205 |
| ssc-miR-17-5p | 0.088178295 | LAMP-1 | 2.8268065 |
| ssc-miR-17-5p | 0.088178295 | SNX16 | 2.076848 |
| ssc-miR-17-5p | 0.088178295 | PRNP | 3.8958378 |
| ssc-miR-17-5p | 0.088178295 | APP | 3.000465 |
| ssc-miR-17-5p | 0.088178295 | OXCT1 | 2.0265446 |
| ssc-miR-17-5p | 0.088178295 | KLF9 | 4.58542 |
| ssc-miR-17-5p | 0.088178295 | FLRT3 | 17.998533 |
| ssc-miR-17-5p | 0.088178295 | MXI1 | 4.065153 |
| ssc-miR-17-5p | 0.088178295 | CAV2 | 14.56954 |
| ssc-miR-17-5p | 0.088178295 | VEGFA | 2.4972408 |
| ssc-miR-17-5p | 0.088178295 | CFL2 | 2.513711 |
| ssc-miR-17-5p | 0.088178295 | TBX3 | 11.339353 |
| ssc-miR-17-5p | 0.088178295 | SOAT1 | 3.0981987 |
| ssc-miR-17-5p | 0.088178295 | SELT | 7.357921 |
| ssc-miR-17-5p | 0.088178295 | APP | 2.6229365 |
| ssc-miR-17-5p | 0.088178295 | ALOX12 | 11.447397 |
| ssc-miR-17-5p | 0.088178295 | BTG3 | 5.4815583 |
| ssc-miR-17-5p | 0.088178295 | SAV1 | 2.919268 |
| ssc-miR-17-5p | 0.088178295 | BTG3 | 3.125916 |
| ssc-miR-17-5p | 0.088178295 | B3GNT5 | 2.965213 |
| ssc-miR-17-5p | 0.088178295 | PTPRU | 2.205255 |
| ssc-miR-17-5p | 0.088178295 | ST3GAL4 | 3.8444028 |
| ssc-miR-17-5p | 0.088178295 | CFL2 | 2.3713303 |
| ssc-miR-17-5p | 0.088178295 | SAV1 | 2.118612 |
| ssc-miR-17-5p | 0.088178295 | PRNP | 4.534527 |
| ssc-miR-17-5p | 0.088178295 | TBX3 | 27.65082 |
| ssc-miR-17-5p | 0.088178295 | SAR1B | 2.1704874 |
| ssc-miR-17-5p | 0.088178295 | THBD | 41.88992 |
| ssc-miR-17-5p | 0.088178295 | TXNIP | 2.4701765 |
| ssc-miR-17-5p | 0.088178295 | CAV2 | 18.59619 |
| ssc-miR-17-5p | 0.088178295 | BTG3 | 3.769016 |
| ssc-miR-17-5p | 0.088178295 | MXI1 | 2.4388943 |
| ssc-miR-17-5p | 0.088178295 | EDNRB | 2.6405814 |
| ssc-miR-17-5p | 0.088178295 | RHOB | 2.6659222 |
| ssc-miR-17-5p | 0.088178295 | TXNIP | 2.7284145 |
| ssc-miR-17-5p | 0.088178295 | TTC8 | 5.671024 |
| ssc-miR-17-5p | 0.088178295 | VEGFA | 2.0567462 |
| ssc-miR-17-5p | 0.088178295 | TXNIP | 2.7425334 |
| ssc-miR-17-5p | 0.088178295 | OLR1 | 3.0760937 |
| ssc-miR-17-5p | 0.088178295 | CNN1 | 3.4723577 |
| ssc-miR-17-5p | 0.088178295 | NPTN | 2.735852 |
| ssc-miR-17-5p | 0.088178295 | THBD | 8.153293 |
| ssc-miR-17-5p | 0.088178295 | ST3GAL4 | 3.185859 |
|  |  |  |  |
| ssc-miR-20a | 0.121069182 | CYP51 | 2.0309756 |
| ssc-miR-20a | 0.121069182 | HP | 35.080257 |
| ssc-miR-20a | 0.121069182 | KLF6 | 3.4863093 |
| ssc-miR-20a | 0.121069182 | ATP1B1 | 3.8346922 |
| ssc-miR-20a | 0.121069182 | SGCE | 3.6834357 |
| ssc-miR-20a | 0.121069182 | ATP1B1 | 5.6342463 |
| ssc-miR-20a | 0.121069182 | EDNRB | 4.742645 |
| ssc-miR-20a | 0.121069182 | OXCT1 | 2.0291076 |
| ssc-miR-20a | 0.121069182 | CFL2 | 2.8280158 |
| ssc-miR-20a | 0.121069182 | OCLN | 6.9868617 |
| ssc-miR-20a | 0.121069182 | SCD | 3.746366 |
| ssc-miR-20a | 0.121069182 | SCD5 | 3.2284265 |
| ssc-miR-20a | 0.121069182 | TEAD4 | 2.688287 |
| ssc-miR-20a | 0.121069182 | IL1A | 2.0862908 |
| ssc-miR-20a | 0.121069182 | KLF10 | 2.6501894 |
| ssc-miR-20a | 0.121069182 | PRNP | 2.4658751 |
| ssc-miR-20a | 0.121069182 | CAPN2 | 11.183791 |
| ssc-miR-20a | 0.121069182 | DUOX2 | 71.88512 |
| ssc-miR-20a | 0.121069182 | BMPR2 | 3.1323707 |
| ssc-miR-20a | 0.121069182 | LYZ | 18.577944 |
| ssc-miR-20a | 0.121069182 | RDX | 2.7281008 |
| ssc-miR-20a | 0.121069182 | OXR1 | 4.7151937 |
| ssc-miR-20a | 0.121069182 | RAB11FIP1 | 5.0282693 |
| ssc-miR-20a | 0.121069182 | ADAMTS1 | 14.238098 |
| ssc-miR-20a | 0.121069182 | RDX | 2.2962925 |
| ssc-miR-20a | 0.121069182 | SCD | 5.3195086 |
| ssc-miR-20a | 0.121069182 | TBX3 | 21.275673 |
| ssc-miR-20a | 0.121069182 | SOD2 | 2.1154256 |
| ssc-miR-20a | 0.121069182 | GUCY2C | 2.614502 |
| ssc-miR-20a | 0.121069182 | KLF9 | 3.7045405 |
| ssc-miR-20a | 0.121069182 | RHOB | 2.540106 |
| ssc-miR-20a | 0.121069182 | SCD | 3.9770987 |
| ssc-miR-20a | 0.121069182 | PRNP | 3.892647 |
| ssc-miR-20a | 0.121069182 | LAMP2 | 2.1236007 |
| ssc-miR-20a | 0.121069182 | EDNRA | 3.8113816 |
| ssc-miR-20a | 0.121069182 | NOR-1 | 3.7146902 |
| ssc-miR-20a | 0.121069182 | KLF9 | 5.3736143 |
| ssc-miR-20a | 0.121069182 | LYZ | 11.652282 |
| ssc-miR-20a | 0.121069182 | OCLN | 30.06069 |
| ssc-miR-20a | 0.121069182 | KLF10 | 2.2958229 |
| ssc-miR-20a | 0.121069182 | OCLN | 30.517065 |
| ssc-miR-20a | 0.121069182 | IL1A | 4.018293 |
| ssc-miR-20a | 0.121069182 | KLF9 | 4.947474 |
| ssc-miR-20a | 0.121069182 | CAV2 | 31.894093 |
| ssc-miR-20a | 0.121069182 | EPAS1 | 3.7910812 |
| ssc-miR-20a | 0.121069182 | RAB22A | 2.8142457 |
| ssc-miR-20a | 0.121069182 | KLF6 | 2.2367525 |
| ssc-miR-20a | 0.121069182 | RHOB | 2.0275912 |
| ssc-miR-20a | 0.121069182 | KLF9 | 5.9025326 |
| ssc-miR-20a | 0.121069182 | FGL2 | 6.0749955 |
| ssc-miR-20a | 0.121069182 | APP | 2.1256132 |
| ssc-miR-20a | 0.121069182 | EPAS1 | 4.391089 |
| ssc-miR-20a | 0.121069182 | S100A14 | 8.052408 |
| ssc-miR-20a | 0.121069182 | KLF9 | 4.886205 |
| ssc-miR-20a | 0.121069182 | LAMP-1 | 2.8268065 |
| ssc-miR-20a | 0.121069182 | ATP1B1 | 5.595114 |
| ssc-miR-20a | 0.121069182 | SNX16 | 2.076848 |
| ssc-miR-20a | 0.121069182 | PRNP | 3.8958378 |
| ssc-miR-20a | 0.121069182 | APP | 3.000465 |
| ssc-miR-20a | 0.121069182 | OXCT1 | 2.0265446 |
| ssc-miR-20a | 0.121069182 | KLF9 | 4.58542 |
| ssc-miR-20a | 0.121069182 | FLRT3 | 17.998533 |
| ssc-miR-20a | 0.121069182 | SCD | 3.4657626 |
| ssc-miR-20a | 0.121069182 | MXI1 | 4.065153 |
| ssc-miR-20a | 0.121069182 | SGCE | 4.3202634 |
| ssc-miR-20a | 0.121069182 | CAV2 | 14.56954 |
| ssc-miR-20a | 0.121069182 | VEGFA | 2.4972408 |
| ssc-miR-20a | 0.121069182 | CFL2 | 2.513711 |
| ssc-miR-20a | 0.121069182 | TBX3 | 11.339353 |
| ssc-miR-20a | 0.121069182 | SOAT1 | 3.0981987 |
| ssc-miR-20a | 0.121069182 | SELT | 7.357921 |
| ssc-miR-20a | 0.121069182 | APP | 2.6229365 |
| ssc-miR-20a | 0.121069182 | BTG3 | 5.4815583 |
| ssc-miR-20a | 0.121069182 | SAV1 | 2.919268 |
| ssc-miR-20a | 0.121069182 | BTG3 | 3.125916 |
| ssc-miR-20a | 0.121069182 | B3GNT5 | 2.965213 |
| ssc-miR-20a | 0.121069182 | ATP1B1 | 4.1853924 |
| ssc-miR-20a | 0.121069182 | ST3GAL4 | 3.8444028 |
| ssc-miR-20a | 0.121069182 | CFL2 | 2.3713303 |
| ssc-miR-20a | 0.121069182 | SAV1 | 2.118612 |
| ssc-miR-20a | 0.121069182 | PRNP | 4.534527 |
| ssc-miR-20a | 0.121069182 | TBX3 | 27.65082 |
| ssc-miR-20a | 0.121069182 | SAR1B | 2.1704874 |
| ssc-miR-20a | 0.121069182 | THBD | 41.88992 |
| ssc-miR-20a | 0.121069182 | TXNIP | 2.4701765 |
| ssc-miR-20a | 0.121069182 | CAV2 | 18.59619 |
| ssc-miR-20a | 0.121069182 | BTG3 | 3.769016 |
| ssc-miR-20a | 0.121069182 | MXI1 | 2.4388943 |
| ssc-miR-20a | 0.121069182 | EDNRB | 2.6405814 |
| ssc-miR-20a | 0.121069182 | RHOB | 2.6659222 |
| ssc-miR-20a | 0.121069182 | TXNIP | 2.7284145 |
| ssc-miR-20a | 0.121069182 | TTC8 | 5.671024 |
| ssc-miR-20a | 0.121069182 | VEGFA | 2.0567462 |
| ssc-miR-20a | 0.121069182 | TXNIP | 2.7425334 |
| ssc-miR-20a | 0.121069182 | OLR1 | 3.0760937 |
| ssc-miR-20a | 0.121069182 | CNN1 | 3.4723577 |
| ssc-miR-20a | 0.121069182 | NPTN | 2.735852 |
| ssc-miR-20a | 0.121069182 | THBD | 8.153293 |
| ssc-miR-20a | 0.121069182 | ST3GAL4 | 3.185859 |

The fold change cutoffs of the upregulated miRNAs/ mRNAs and the downregulated miRNAs/ mRNAs were 2 and 0.5.

**Table S6. DE miRNAs in vivo and vitro under viral infection**

| **Mature miRNA** | **Type** | **JEV (Japanses Encephalitis virus) infected Porcine kidney epithelial cells** | **PPV (Porcine parvovirus) infected PK-15 cells** | **PCMV infected macrophages**  **Fold change value** | **PCMV infected lung Fold change value** | **PCMV infected**  **Liver Fold change value** | **PCMV infected**  **spleen Fold change value** | **PCMV infected**  **thymus Fold change value** | **PCMV infected**  **kidney Fold change value** |
| --- | --- | --- | --- | --- | --- | --- | --- | --- | --- |
| Ssc-miR-101 | Up-regulated | 5.49 |  | 18.60 | 4.18 |  |  |  |  |
| Ssc-miR-1 | Up-regulated | 8.03 |  | 3.04 | 3.78 |  |  |  |  |
| Ssc-miR-10a | Up-regulated | 4.04 |  | 3 | 3.78 | 3 |  | 2.53 | 2.53 |
| Ssc-miR-128 | Up-regulated |  |  | 4.97 |  |  |  | 2.53 |  |
| Ssc-miR-155-5p | Up-regulated |  | 6.06 | 19.67 | 6.26 | 3.39 | 5.93 |  |  |
| Ssc-miR-192 | Up-regulated | 33.7 |  | 2.45 | 10.55 |  |  |  |  |
| Ssc-miR-450c-5p | Up-regulated | 14.81 |  | 3.13 |  |  |  |  | 9.03 |
| Ssc-miR-7d-5p | Down-regulated | 0.22 |  | 0.10 | 0.12 | 0.05 |  |  |  |
| Ssc-miR-15a | Down-regulated | 0.35 |  | 0.22 |  | 0.18 |  |  | 0.15 |
| Ssc-miR-185 | Down-regulated | 0.23 | 0.04 | 0.07 | 0.47 |  |  |  |  |
| Ssc-miR-18a | Down-regulated | 0.01 | 0.01 | 0.13 |  |  |  | 0.11 |  |
| Ssc-miR-19a | Down-regulated | 0.41 |  | 0.27 |  |  |  | 0.28 |  |
| Ssc-miR-22-3p | Down-regulated | 0.03 |  | 0.44 | 0.36 |  |  |  |  |
| Ssc-miR-27b-5p | Down-regulated | 0.37 |  | 0.34 | 0.35 |  | 0.27 |  |  |
| Ssc-miR-28-5p | Down-regulated | 0.39 |  | 0.22 | 0.13 |  |  |  |  |
| Ssc-miR-301 | Down-regulated | 0.37 |  | 0.37 | 0.36 | 0.5 |  |  |  |
| Ssc-miR-421-3p | Down-regulated | 0.27 |  | 0.29 | 0.19 | 0.25 |  |  |  |

The fold change cutoffs of the upregulated miRNAs and the downregulated miRNAs were 2 and 0.5.

**Table S7. Primers used for stem-loop qRT-PCR.**

| **Genes** | **Primers** | **Products length (bp)** |
| --- | --- | --- |
| U6 | F:5’TCGCTTTGGCAGCACCTAT3’ R:5’AATATGGAACGCTTCGCAAA3’ | 100 |
| ssc-miR-10b | GSP:5’TACCCTGTAGAACCGAATTTGT3’ R:5’GTCGGTGTCGTGGAGTCG3’ | 76 |
| ssc-miR-486 | GSP:5’TCCTGTACTGAGCTGCCCCGAG3’ R:5’GTCGGTGTCGTGGAGTCG3’ | 76 |
| ssc-miR-24-3p | GSP:5’TGGCTCAGTTCAGCAGGAACAG3’ R:5’GTCGGTGTCGTGGAGTCG3’ | 76 |
| ssc-miR-195 | GSP:5’TAGCAGCACAGAAATATTGGC3’ R:5’GTCGGTGTCGTGGAGTCG3’ | 75 |
| ssc-miR-19b | GSP:5’TGTGCAAATCCATGCAAAACTGA3’ R:5’GTCGGTGTCGTGGAGTCG3’ | 77 |
| ssc-let-7f | GSP:5’TGAGGTAGTAGATTGTATAGTT3’ R:5’GTCGGTGTCGTGGAGTCG3’ | 76 |
| ssc-miR-146b | GSP:5’TGAGAACTGAATTCCATAGGC3’ R:5’GTCGGTGTCGTGGAGTCG3’ | 75 |
| ssc-miR-novel-chr16_17559 | GSP:5’GGGTGGCAGTGTATTGTTAGC3’ R:5’GTCGGTGTCGTGGAGTCG3’ | 79 |
| ssc-miR-novel-GL892871-2_41708 | GSP:5’GGGTTCAAGTAATTCAGGATAGGT3’ R:5’GTCGGTGTCGTGGAGTCG3’ | 79 |
| ssc-miR-novel-chr2_21624 | GSP:5’GGGTGAGATGAAGCACTGTAGC3’ R:5’GTCGGTGTCGTGGAGTCG3’ | 77 |
| ssc-miR-novel-chr12_7961 | GSP:5’GCAGGGCAATAGTATTGTCAAAG3’ R:5’GTCGGTGTCGTGGAGTCG3’ | 77 |
| ssc-miR-26a | GSP:5’GTTCAAGTAATCCAGGATAGGC3’ R:5’GTCGGTGTCGTGGAGTCG3’ | 77 |

References:

1. Identification and Analysis of Differentially-Expressed microRNAs in Japanese Encephalitis Virus-Infected PK-15 Cells with Deep Sequencing. Int. J. Mol. Sci. 2015, 16, 2204-2219

2. Differential expression of microRNAs in porcine parvovirus infected porcine cell line. Virology Journal (2015) 12:128

3. Liu X, Liao S, Xu Z, Zhu L, Yang F, Guo W (2016) Identification and Analysis of the Porcine

MicroRNA in Porcine Cytomegalovirus-Infected Macrophages Using Deep Sequencing. PLoS ONE

11(3): e0150971. doi:10.1371/journal.pone.0150971
